# Recapturing Cooperativity of α‑Helix Formation and Packing in Coarse-Grained Protein Structure Modeling with Multitorsional Potentials

**DOI:** 10.1021/acs.jpcb.5c03985

**Published:** 2025-07-03

**Authors:** Elizaveta F. Petrusevich, Adam Liwo

**Affiliations:** Faculty of Chemistry, 49646 University of Gdańsk, Fahrenheit Union of Universities in Gdańsk, Wita Stwosza 63, Gdańsk 80-308 Gdańsk, Poland

## Abstract

The multitorsional potential accounting for cooperativity of local interactions proposed in our previous work (Sikorska & Liwo, *J. Phys. Chem. B*, **2022**, 126, 9493–9505; **2023**, 127, 425–426) has been introduced into the UNRES coarse-grained force field. The parameters of the potential have been found by means of maximum-likelihood principle using the data of 1,092,517 helical segments of protein structures of the Protein Data Bank. The modified UNRES has been tested with a set of 28 α-helical proteins with size from 20 to 126 amino acid residues and various topologies. With the best parametrization, the first-choice models were significantly improved for 10 proteins (ΔGDT_TS > 5) and deteriorated for 3 proteins (ΔGDT_TS < −5). The improvement resulted from strengthening of the helical sections and improving the geometry of the fragments following helix ends, thus enabling correct packing. Overstrengthening the helical sections was the main reason for model deterioration.

## Graphical abstract

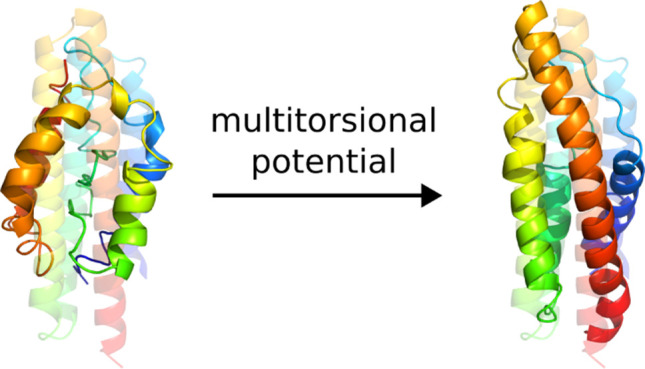

## 1 Introduction

Protein folding is a highly cooperative phenomenon,^1–5^ which involves the interplay of a variety of interactions, including solvent-mediated or hydrophobic interactions, hydrogen-bonding, local interactions, steric repulsion, and electrostatic interactions between charged residues.^4,6^ Of those, hydrophobic interactions are responsible for side-chain packing, leaving polar and charged residues mainly outside the formed structures and forming a hydrophobic core of nonpolar ones, while backbone-hydrogen-bonding and backbone-local interactions are the main driving forces in forming secondary structure.^6^

Because none of the driving forces mentioned above can be considered dominant,^6^ physics-based modeling of protein folding poses a major problem, mainly with regard to the construction of sufficiently accurate force fields that could account for these minute differences. While knowledge-based methods^7–9^ in particular, Deep Mind’s AlphaFold,^10^ AlphaFold2 ^11–13^ and AlphaFold3 ^14^ have effectively solved the problem of protein-structure prediction, the development of physics-based approaches is still needed to model and understand the process of protein folding and association, protein–protein interactions, etc.

Despite the advances in constructing still more powerful supercomputers, including machines dedicated to molecular-dynamics simulations,^15^ all-atom simulations of protein folding still cover only small proteins. Coarse-grained models, in which groups of atoms are merged into extended sites, enable us to extend both time- and size-scale of simulations by orders of magnitude.^16,17^ Both knowledge- and physics-based approaches are implemented in constructing coarse-grained force fields.^16,17^ It should be noted that the knowledge-based (statistical) coarse-grained potentials such as, e.g., CABS^18^ are successful not only in modeling protein structures but also in folding simulations.^19^ Recently, coarse-grained potentials derived by machine learning have been proposed, which are successful in modeling protein structures, folding pathways, and free-energy landscapes.^20,21^

The construction of coarse-grained force fields capable of modeling protein folding is even more difficult than that of all-atom force fields. The main reason for this is the need for the inclusion of multibody or correlation terms.^22–24^ These terms are essential for a force field to be capable of modeling α-helices and β-sheets, which are kept together by highly cooperative interactions.^22–25^ It should be noted that all-atom force fields also benefit from introduction of cooperative (multibody) terms with regard to enhanced cooperativity of α-helix and β-hairpin formation in simulations.^26^ The role of cooperativity in the formation of secondary structure was recognized already in the early Zimm–Bragg theory of helix–coil transition,^27^ which was further developed by Poland and Scheraga.^28^ The cooperativity in the formation of secondary-structure elements is thought to originate primarily in hydrogen-bond formation,^25,29,30^ which can be understood as the alignment of peptide-bond dipoles^31,32^ but at least part of it seems to arise from multibody effects such as electron-density redistribution.^33^

In the UNRES coarse-grained model developed in our laboratory,^34,35^ we initially introduced the terms accounting for the coupling in hydrogen-bond formation,^36^ however, the cooperative terms pertaining to the coupling of backbone-hydrogen-bonding and backbone-local interactions turned out to be essential for correct secondary-structure modeling.^22,23^ These terms have been derived on physics grounds, by means of the recently developed scale-consistent theory of coarse graining.^23,24^ They are third-order correlation terms, each encompassing a pair of interacting peptide groups and the adjacent residues. With these energy terms, UNRES is able to model proteins with relatively simple topologies in the *ab initio* mode, as demonstrated in the Community Wide Experiment on the Critical Assessment of Techniques for Protein Structure Prediction (CASP) exercises.^37–39^ However, more complex folds pose the problem of appropriate packing of secondary-structure elements, which is partially controlled by the direction of the chain immediately preceding or following an α-helix or a β-strand. Without correct modeling of the packing of secondary-structure elements, supersecondary, tertiary, and quaternary structure cannot be modeled correctly.

By analyzing the dependence of the similarity of the models of protein structure from the CASP14 exercise, obtained with all methods, including AlphaFold, quantified as the Global Distance Test Test Score (GDT_TS),^40,41^ we found that GDT_TS drops with chain length.^39^ We concluded that this tendency could be connected with the absence of long-range correlations, both in the bioinformatics and physics-based approaches. For this reason, in our recent work,^42^ we used the scale-consistent theory of coarse graining to derive the cooperative terms pertaining to the coupling of local interactions of the consecutive residues. We found that these terms have the form of multitorsional potentials that involve the consecutive backbone C^α^···C^α^···C^α^···C^α^ virtual-bond dihedral angles γ (cf. Figure 1) along the chain segment under consideration. While the general expression contains a sum of cosines in all possible combinations of the phase-shifted γ angles with plus and minus signs, it gets simplified to a single cosine term in the sum of all phase-shifted γs for the extended and a product of the cosines and sines (the latter at segment ends) of the phase-shifted γs for those that are not extended, which we term folded (F), mostly α-helical (abbreviated with FH), segments.^42^

**Figure 1:**
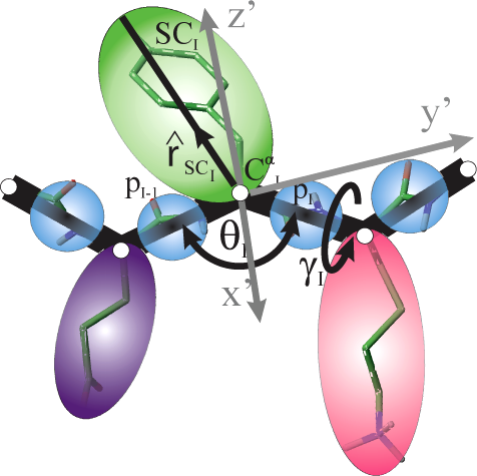
UNRES model of polypeptide chains. The interaction sites are united peptide groups (p; shown as light-blue spheres), each positioned halfway between the two consecutive α-carbon (C^α^) atoms (shown as small white circles), and united side chains (SC; shown as spheroids with different colors) attached to the corresponding C^α^s. The geometry of the chain is defined by the positions of the C^α^ atoms, which are not interaction sites, and those of the side-chain centers. Local backbone geometry is defined by the C^α^···C^α^···C^α^ virtual-bond angles θ and the C^α^···C^α^···C^α^···C^α^ virtual-bond-dihedral angles γ indicated in the Figure. The orientation of a side-chain center with respect to the local backbone fragment composed of three consecutive C^α^ atoms is defined by the unit vector, ${\mathrm{r̂}}_{{\mathrm{SC}}_{i}}$ , expressed in the local coordinates system with origin at $C_{i}^{\alpha }$ , whose *x* axis (*x*′) is the bisector of angle θ_*i*_, the *y* axis (*y*′) runs in the direction of the chain and lies in the plane of the three consecutive C^α^ atoms and the *z* axis (*z′*) forms a right-handed orthogonal coordinate system with the *x′* and *y′* axes. For illustration, atom–atom bonds, except for those that connect the hydrogen atoms the carbon atoms, are superposed on the coarse-grained picture. Adapted with permission from Zaborowski et al., *J. Chem. Inf. Model.,* **2015**, 55, 2050–2070. Copyright 2015 American Chemical Society.

By analysis of 77,950 structures from the Protein Data Bank (PDB),^43^ we found that the multitorsional terms mentioned above are manifested in the distributions of the γ angles of the chain sections preceding and following β-strands and α-helices, respectively.^42^ These results suggest that the coupling between backbone-local interactions is an important factor contributing to the cooperativity in the formation of secondary-structure elements and, thereby, to their stability. Based on these results, we proposed the expressions for multitorsional terms that could stabilize α-helical and β-strand segments and direct chain segments at their ends to promote proper packing of secondary-structure elements.

In this work, we have introduced the multitorsional potentials predicted to promote the F and FH segments^42^ into UNRES, parametrized them based on the FH segments extracted from the protein structures of the PDB, and tested UNRES augmented with the multitorsional potentials with a set of 28 α-helical proteins with different topologies, which were used to evaluate UNRES in our previous work.^44^ It should be noted that these additional potentials are not imposed just on sections predicted by external methods as α-helical but pertain to the whole sequence. We found that the new terms improved the modeled structures.

## 2 Theoretical Methods

### 2.1 UNRES Model of Polypeptide Chains

A polypeptide chain in the UNRES model is defined by the trace of the α-carbon (C^α^) atoms, connected with virtual bonds with equilibrium length of 3.8  Å corresponding to *trans* peptide groups, and united side chains (SC) attached to the respective C^α^s with virtual bonds of equilibrium lengths depending on side-chain type. United peptide groups (p) are located in the middle between the consecutive C^α^s. Only the SCs and the ps are interaction sites,^34,35,45^ (Figure 1). The geometry of the coarse-grained chain is defined by the Cartesian coordinates of the C^α^ atoms and those of SC centers. The C^α^···C^α^···C^α^ backbone-virtual-bond angles θ and the C^α^···C^α^···C^α^···C^α^ backbone-virtual-bond dihedral angles γ are also defined for the calculations of the effective energy terms (Figure 1).

The effective energy function originates from the potential of mean force (PMF) of polypeptide chains in water, in which all degrees of freedom not included in the model are integrated out.^22–24^ It consists of the site–site ( $U_{{\mathrm{SC}}_{i}{\mathrm{SC}}_{j}}$ , $U_{{\mathrm{SC}}_{i}p_{j}}$ , $U_{p_{i}p_{j}}^{\mathrm{VDW}}$ , and $U_{p_{i}p_{j}}^{\mathrm{el}}$ ) terms, the local-interaction terms that, in turn, comprise the C^α^···C^α^ and C^α^···SC virtual-bond potentials (*U*_bond_), the virtual-bond angle potentials (*U*_b_), the virtual-bond-dihedral angle potentials (*U*_tor_), and the potentials dependent on orientation of the united side chains with respect to polypeptide backbone (*U*_rot_), the correlation or multibody terms, $U_{\mathrm{corr}}^{\left(3\right)}$ and $U_{\mathrm{turn}}^{\left(3\right)}$ , and the *U* ^ssbond^ terms that account for the formation and breaking the disulfide bonds.^46^ The energy expression is given by eq 1.

1 U=wSCSC∑i<jUSCiSCj+wSCp∑i≠jUSCipj+wppVDW∑i<j−1UpipjVDW+f2(T)wppel∑i<j−1Upipjel+wssbond∑Cys⁡pairsk,lUk,lssbond+wbond∑kUbond(dk)+wb∑iUb(θi)+wrot∑iUrot(θi,r̂SCi)+f2(T)wtor∑iUtor(γi,θi,θi+1)+f3(T)wcorr(3)∑i<j−2Ucorr;pipj(3)+f3(T)wturn(3)∑iUturn;pipi+2(3)

where the *d*_*k*_ is the *k*th virtual-bond length, θ_*i*_ and γ_*i*_ are the *i*th virtual-bond-angle and virtual-bond-dihedral angle, respectively, and ${\mathrm{r̂}}_{{\mathrm{SC}}_{i}}$ is unit vectors pointing from the *i*th C^α^ atom to the side-chain center expressed in the local coordinate system of residue *i* (Figure 1). The *w*s are the energy-term weights that are determined by force-field calibration.^44^ The multipliers *f*_*n*_(*T*), defined by eq 2,^47^ account for the temperature dependence of the effective energy, which arises from the fact that the this quantity is an approximation to the PMF.

2 $$f_{n}\left(T\right)=\frac{\ln\left[\exp\left(1\right)+\exp\left(-1\right)\right]}{\ln\left\{\exp\left[{\left(\frac{T}{T_{o}}\right)}^{n-1}\right]+\exp\left[-{\left(\frac{T}{T_{o}}\right)}^{n-1}\right]\right\}}$$

where *T*_o_ = 300 K.

The $U_{{\mathrm{SC}}_{i}{\mathrm{SC}}_{j}}$ , and $U_{p_{i}p_{j}}^{\mathrm{el}}$ site–site terms in eq 1 depend on both distance between the site centers and the orientation of the respective virtual-bond axes. This feature and the presence of correlation terms, which account for the stabilization of the regular secondary structures^22–24^ enable us to do *ab initio* modeling of the structures of small and medium-size proteins, as proved in the Community Wide Experiments.^38,39^ It should also be noted that each of the torsional terms, *U*_tor_(γ*_i_,* θ*_i_,* θ*_i_*_+1_), depends not only on the respective virtual-bond dihedral angle γ but also on the virtual-bond dihedral angles θ at the residues adjacent to the axis of this dihedral angle. This feature follows directly from the scale-consistent theory of coarse graining and enables us to model the local-chain geometry of the α-helical and β-strand sections, where the torsional potentials favor open and nearly right θ angles, respectively.^23,44^

In this work, we used the scale-consistent NEWCT-9P variant of UNRES calibrated in our earlier work^44^ with 9 proteins with different type of secondary structure and different folds as the basis. This variant of UNRES is hereafter referred to as the “state-of-the-art” UNRES. The state-of-the-art UNRES was augmented with adding the new multitorsional potentials, $U_{\mathrm{mtor}}^{f}$ , described in Section 2.2 to eq 1.

### 2.2 Multitorsional Potential Energy Terms and Their Implementation in UNRES

The multitorsional potential for the folded chain segment (see Figure 2 for illustration) starting at residue *i* and extending to residue *i* + *m*-1 (with length of *m* residues) proposed in ref 42 is defined by eq 3.

3 Umtor;i;mf=−∑M=1MmaxwM[sin⁡θi+1]M⁡sin[M(γi+1+Ψi+1)]×[∏k=i+2i+m−4[(sin⁡θk)2]M⁡cos[M(γk+Ψk)]]×[(sin⁡θi+m−3)2]M⁡sin[M(γi+m−3+Ψi+m−3)][sin⁡θi+m−2]M

where *M* is the multiplicity of the term (in this work we use the terms of *M* = 1 or *M* = 2 or a combination thereof, thus *M*_max_ = 2), θ_*k*_ is the backbone virtual-bond angle with vertex at $C_{k}^{\alpha }$ , γ_*k*_ is the virtual-bond dihedral angle with axis at the $C_{k}^{\alpha }\cdot \cdot \cdot C_{k+1}^{\alpha }$ virtual bond (cf. Figure 1), *w*_*M*_ is the weight of the multitorsional energy term with multiplicity *M*, and Ψ_*k*_ is the phase angle specific of residue types. The complete multitorsional energy of a polypeptide chain containing *n*_res_ residues is expressed by eq 4. The expressions for the multitorsional energy contribution and their gradients in C^α^ coordinates optimized for computations are presented in Section S1.

4 $$U_{\mathrm{mtor}}^{f}=\overset{m_{\max}}{\underset{m=7}{\sum }}\overset{n_{\mathrm{res}}-m+1}{\underset{i=1}{\sum }}U_{\mathrm{mtor};i;m}^{f}$$

where *m*_max_ is the maximum length of the backbone segment subjected to the multitorsional potential; in this work we set *m*_max_ = 20.

**Figure 2:**
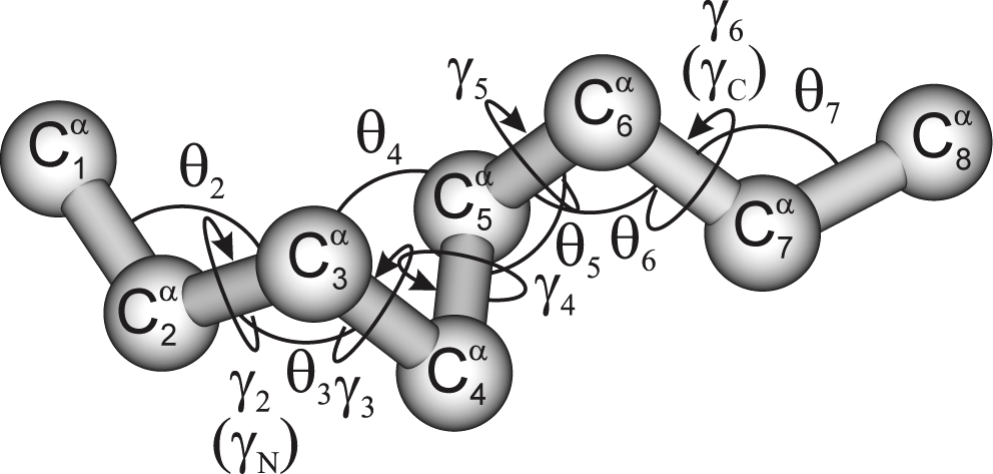
Illustration of folded (F) sections of polypeptide chains with an 8-residue fragment. Gray spheres represent the C^α^ atoms (numbered from 1 to 8), which are linked with virtual bonds. The C^α^···C^α^···C^α^ virtual-bond angles θ and the C^α^···C^α^···C^α^···C^α^ virtual-bond dihedral angles γ are indicated. The angles θ are about 90° except for the ends, hence the segment is termed “folded”. The angles γ are positive and less than 90° except the ends, which is characteristic of α-helical segments. The end γ angles are additionally labeled γ_N_ and γ_C_, respectively. Adapted from Sikorska & Liwo, *J. Phys. Chem. B*, **2022**, 126, 9493–9505, under the Creative Commons (CC-BY 4.0) license.

The factors (sin θ_*i*+2_)^2^, ···, (sin θ_*i*+_ *_m_*_–3_)^2^ take the maximum for θ = 90°, as in the F (mainly FH) segments, and quickly decay with θ angles departing from 90°. Therefore, $U_{\mathrm{mtor};i;m}^{f}$ , become much less important for extended chains. Consequently, augmenting UNRES with $U_{\mathrm{mtor}}^{f}$ does not require including explicit information about secondary structure and can thus be used in *ab initio* simulations of protein folding. Higher than two powers of sin θ or other functions in this variable could be used to restrict the θs inside the folded segment to the neighborhood of 90^◦^ even more. However, we found the current choice sufficient, while steeper functions of sin θ could lead to numerical problems. The angles θ_*i*+1_ and θ_*i*+*m*–2_ at the ends of the folded segment are not restricted to around 90°.

The phase angles Ψ control the stability of helical fragments (the cos­[*M*(γ + Ψ)] factors inside the segment) and the direction of the C^α^···C^α^ virtual bonds immediately preceding and following the F and FH segment (the sin­[*M*(γ + Ψ)] factors at the ends of the segments). These phase angles are the parameters to be determined.

In this work, the multitorsional component is added to the UNRES energy function with a weight, as given by eq 5.

5 $$U=U_{\mathrm{UNRES}}+f_{4}\left(T\right)w_{\mathrm{mtor}}U_{\mathrm{mtor}}^{f}$$

where *U*_UNRES_ is the UNRES energy given by eq 1, $U_{\mathrm{mtor}}^{f}$ is expressed by eq 4, *w*_mtor_ is the weight of the multitorsional terms, and *f*_4_(*T*) is defined by eq 2. We used the scale-consistent NEWCT-9P variant of UNRES calibrated in our earlier work with 9 proteins with different type of secondary structure and different folds.^44^ The weights of the multitorsional terms, *w*_mtor_, ranged from 0 to 0.2 (see the Results and Discussion section).

### 2.3 Parameterization of the Multitorsional Potentials

In this study, we determined the parameters (the weights *w*_*M*_ and the phase angles Ψ) of the multitorsional potentials by means of the maximum-likelihood approach, which was used to parametrize the local potentials of the early UNRES^48^ and to calibrate the recent NEWCT-9P UNRES variant.^44^ This approach is based on maximizing the logarithm of the maximum-likelihood function corresponding to the probability-density function given by the model for the distribution of the experimental points. In this work, the experimental points are the sets of consecutive angles θ and γ calculated from 1,092,517 FH segments of polypeptide chains extracted from 77,950 structures from the PDB, as described in our earlier work.^42^ An *m*-residue segment starting at residue *k* is defined as folded helical if θ*_i_* < 100°, *i* = *k* + 2, *k* + 3, ···, *k* + *m* −3, and γ_*k*+2_ through γ_*k*+*m*–4_ are contained within the interval from 0° to 70° or that the residues with indices from *k* + 1 through *k* + *m* – 2 are in the HELIX records of a respective PDB entry.^42^

Given the fact that local geometry is mainly governed by local interactions and assuming that the multitorsional terms are mainly responsible for the long-range correlations, the multidimensional distribution of consecutive θ and γ angles of the folded-helical fragments with length *n* can be assumed to obey the Boltzmann distribution with energy given by $V_{\mathrm{mtor};n}^{f}\left(\Theta_{j}^{\left(n\right)};\Gamma_{j}^{\left(n\right)}\right)$ , where $\Theta_{j}^{\left(n\right)}$ and $\Gamma_{j}^{\left(n\right)}$ are the shorthands for all θ and γ angles in the *j*th folded-helical fragment with length *n* and $V_{\mathrm{mtor};n}^{f}$ is the sum of all possible $U_{\mathrm{mtor};i;k}^{f},\;7\leq k\leq n$ defined for such a fragment, as given by eq 6, which is akin to $U_{\mathrm{mtor}}^{f}$ of eq 4 except that the latter covers the whole polypeptide chain irrespective of structure.

6 Vmtor;nf=−∑m=720∑i=1nres−m+1∑M=12wM[sin⁡θi+1]Msin[M(γi+1+ΨK(i+1),K(i+2))]×[∏k=i+2i+m−4[(sin⁡θk)2]Mcos[M(γk+ΨK(k),K(k+1))]]×[(sin⁡θi+m−3)2]Msin[M(γi+m−3+ΨK(i+m−3),K(i+m−2))][sin⁡θi+m−2]M

where *K*(*i*) is the kind of residue with index *i*.

For clarity, in eq 6, we reset the index of the first residue of the fragment to 1.

Consequently, the optimization problem is formulated as minimization of *L* defined by eq 7, which is the negative of the logarithm of the maximum-likelihood function.

7 L=−∑n=7nmax∑jln[exp(−Vmtorf(Θj(n),Γj(n);w;Ψ)RT)Qn]=∑n=7nmax∑j[Vmtorf(Θj(n),Γj(n);w;Ψ)RT+ln⁡Qn(w;Ψ)]

where $\Theta_{j}^{\left(n\right)}$ and $\Gamma_{j}^{\left(n\right)}$ are shorthands of all θ and γ angles, respectively, of the FH segment *j* that has length *n*, **w** and **Ψ** are the shorthands of the weights and phase angles to be determined, respectively, *R* is the universal gas constant, *T* is the absolute temperature, and *Q*_*n*_ is the partition function for an FH fragment with length *n*, which is expressed by eq 8.

8 $$Q_{n}\left(w;\Psi \right)=\int \cdots \int \exp\left[-\frac{V_{\mathrm{mtor};n}^{f}\left(\Theta^{\left(n\right)},\Gamma^{\left(n\right)};w;\Psi \right)}{RT}\right]d\Theta^{\left(n\right)}d\Gamma^{\left(n\right)}$$

As indicated, the parameters to be determined are the phase angles Ψ and weights of terms with different multiplicity, *w*_*M*_. When the weights are not optimized, the partition functions *Q*_*n*_ do not change with changing the phase angles and, consequently, minimization of *L* of eq 7 becomes equivalent to finding a set of phase angles that minimizes the multitorsional energies summed over all of the FH fragments in the database. When the weights are to be optimized, the multidimensional integrals required to compute *L* should be evaluated. For this reason, we decided to do a limited scan over a grid of *w*_1_ and *w*_2_ with keeping *w*_1_ + *w*_2_ = 1, optimizing *L* without the ln*Q*_*n*_ components and then selecting the results with low *L*.

The phase angles Ψ depend on the kind of amino-acid residues at the C^α^···C^α^ virtual bond, which is the axis (root) of the respective virtual-bond dihedral angle γ. The local interactions depend primarily on the immediate surrounding of the C^α^ atoms, which leads to three basic types of residues, namely glycine (no side chain), proline (a five-membered ring that severely restricts the conformational space), and alanine, which stands for all remaining residue types.^22,23,49,50^ This division results in 9 different phase angles Ψ.

The selection of three basic residue types is justified on Figure 3, which shows the distributions and the number of the dihedral angles composing the training data set for all 9 pairs. As can be seen, the distributions are remarkably different. The pairs with Ala exhibit the global maximum at γ = 50°, while those containing Pro or Gly exhibit a second pronounced maximum at γ = 240°. For the Gly-Gly pairs, the distribution of γ is more symmetric about 180° because of the symmetry of the Gly energy surface.

**Figure 3:**
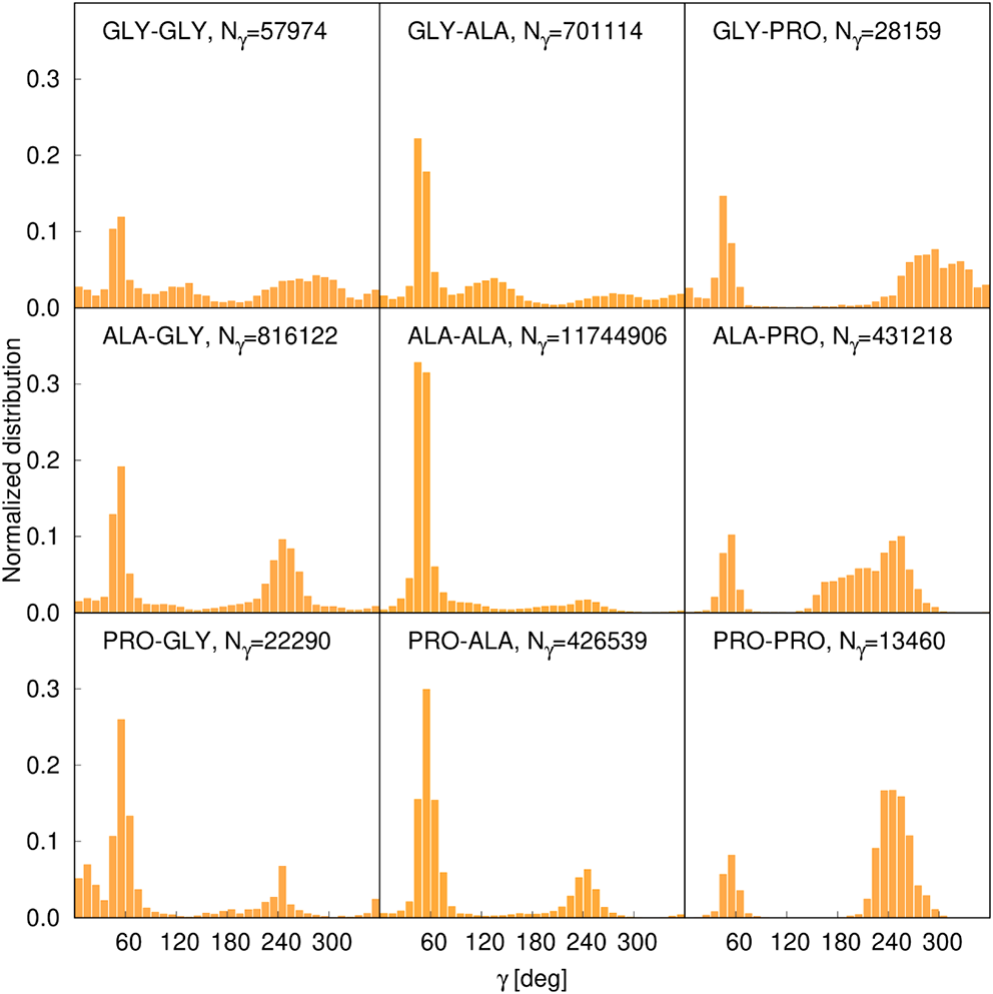
Distributions of virtual-bond-dihedral angles γ for the 9 pairs of basic residue types derived from the 1,092,517 FH fragments extracted from the PDB in our earlier work^42^ and used in this work to parametrize the multitorsional potentials. The plots were made with gnuplot.^51^

To minimize *L* [eq 7], we used the secant unconstrained minimization solver (SUMSL) algorithm with analytical derivatives in optimized parameters.^52^

### 2.4 Evaluation of UNRES with Multitorsional Terms

Because the $U_{\mathrm{mtor};i;m}^{f}$ terms considered in this work pertain to α-helical sections of the structure, we tested UNRES augmented with the multitorsional potentials with a subset of 28 α-helical single-chain proteins used to evaluate UNRES in our earlier work.^44^ The PDB IDs and chain lengths (in parentheses) of these proteins are 1A1W (83), 1A6S (87), 1ACP (77), 1BBL (37), 1BG8 (76), 1CLB (75), 2CRB (97), 1E68 (70), 1ENH (54), 1EO0 (77), 1FEX (59), 1GAB (48), 2HEP (42), 1HYP (75), 1J7O (76), 1K40 (126), 1KOY (62), 2L09 (62), 1L2Y (20), 1LQ7 (67), 1P68 (102), 1POU (71), 1PPT (36), 1PRU (56), 1RES (43), 1RIJ (23), 2YGS (92), and 1YRF (35), respectively.

We tested UNRES with each set $U_{\mathrm{mtor}}^{f}$ parameters obtained in this work by applying our *ab initio* protein-structure modeling protocol^37,38^ to each benchmark protein of the list above. Briefly, in the first stage, unrestricted simulations were carried out by using multiplexed replica-exchange molecular dynamics (MREMD)^53^ adapted to UNRES in our earlier work.^54^ It should be noted that the secondary structure was not predefined in these calculations. In particular, no input from secondary-structure prediction methods such as, e.g., PSIPRED^55^ was used and no use was made from the secondary-structure information in the respective PDB entries. In the second stage, the conformations of the last 1,000 snapshots from each replica trajectory with sampling frequency of 16 (a total of 3,024 conformations) were processed with the weighted histogram analysis method (WHAM),^56^ adapted to the temperature-dependent UNRES.^47^ This step enabled us to determine the weights of the conformations at the desired temperatures. In the third stage, cluster analysis was performed on a subset of conformations that constituted 99% of the ensemble at the desired temperature (300 K in this work) to dissect the set of conformations into 5 clusters, by using Ward’s minimum-variance method.^57^ The clusters were ranked following the cumulative probabilities of the constituent conformations. The conformation closest to the Boltzmann-averaged conformation of a given cluster was selected as the representative of this cluster. Finally, in the fourth stage, the conformations selected in the third stage were converted to the all-atom representation by using the cg2all algorithm,^58,59^ followed by refinement with AMBER^60^ with the ff19SB force field^61^ and implicit-solvent Generalized Born Surface Area (GBSA) model^62,63^ as described in our earlier work.^38^

Each MREMD run consisted of 12 4-plexed replicas (48 replicas/trajectories total) run at temperatures *T* = 260, 272, 279, 284, 288, 291, 294, 298, 308, 322, 341, and 370 K. These temperatures were selected by means of the Hansmann algorithm^64^ to maximize the number of walks of the replicas in the temperature space. Each trajectory consisted of 20,000,000 steps with 4.89 fs step length. We used the UNRES implementation of Langevin dynamics with the variable time step (VTS) velocity-Verlet algorithm to integrate the equations of motion.^65,66^ The calculations were run with the recently optimized UNRES code,^67^ with modifications due to introducing the multitorsional terms. Each trajectory started from a randomly generated conformation, subject to the condition of nonoverlap, as described in our earlier work.^68^ The replicas were exchanged every 10,000 steps and the snapshots from trajectories were collected with the same frequency.

Three measures of the similarity of the models to the respective experimental structures were used for evaluation of MREMD simulations, namely the root-mean-square deviation of the C^α^-atom positions (C^α^-RMSD or, in short, RMSD) at the optimal superposition of the C^α^ atoms of the target structure on those of the reference structure, the GDT_TS,^40,41^ and the template modeling score (TM-score).^69^ GDT_TS is an average of the percentages of the target structure whose C^α^ atoms superpose within 1, 2, 4, and 8 Å, respectively, on those of the reference structure. While RMSD has the explicit sense of distance between two conformations, it becomes a poor measure when the two structures are similar only in part; in this instance GDT_TS enables us much better to judge similarity. TM-score^69^ is a measure of reciprocal square of the distances of the residues in the model and in the reference structure at optimal superposition of the two structures. The TM-score software from Zhang’s lab^69^ was used to calculate similarity measures.

## 3 Results and Discussion

### 3.1 Parameters and Topological Features of Multitorsional Energy Terms

The phase angles Ψ, obtained by minimizing the target function *L* of eq 7 for the grid of weights of terms with multiplicity 1 and 2 [eq 3], keeping the sum of weights at 1 and selecting the sets with the lowest *L*, are summarized in Table 1. As can be seen, three sets of parameters, one corresponding to multiplicity 1, one to mixed multiplicity, and the last one to multiplicity 2 only were obtained. These sets are hereafter referred to as set A, B, and C, respectively.

**Table 1:** Optimized Phase Angles Ψ for All Pairs of Basic Residue Types (Glycine, Alanine, and Proline) Denoted with One-Letter Code

Set	*A*	*B*	*C*
*w*_*M*_	*w*_1_ = 1.0	*w*_1_ = 0.6	*w*_1_ = 0.0
eq 3	*w*_2_ = 0.0	*w*_2_ = 0.4	*w*_2_ = 1.0
Optimized phase angles [deg]			
Ψ_GG_	–12.4	–24.6	–31.6
Ψ_GA_	–8.4	–26.2	–35.1
Ψ_GP_	–19.3	41.0	–27.2
Ψ_AG_	–36.3	–31.5	–30.7
Ψ_AA_	–15.5	–27.1	–33.4
Ψ_AP_	–58.2	–99.0	–11.1
Ψ_PG_	–9.5	7.2	–35.7
Ψ_PA_	–3.4	13.5	–31.2
Ψ_PP_	–79.9	–85.0	–32.1

Except for those corresponding to pairs with proline, the phase angles are small in absolute value. Most of them, in particular Ψ_AA_, which corresponds to the most common pair of reduced residue type, are negative. This is understandable because, except for the shortest FH segments, most of the contributions to the sum of multitorsional terms pertaining to a given, $V_{\mathrm{mtor};n}^{f}$ of eq 6 arise from fragments buried inside an FH segment. Therefore, the component with sin­[*M*(γ + Ψ)] pertains not only to the terminal dihedral angles, but also to those inside an FH segment. The γ angles for an α-helix range from about 30° to about 70° and, therefore, the cosine terms are positive and not too small in absolute value if the Ψ angles are negative and small in absolute value. Positive values of the cosine terms prevent the components of $V_{\mathrm{mtor};n}^{f}$ from changing sign depending on fragment length. They can be regarded as contributing to helix stabilization, which is consistent with the cooperativity of helix formation.^28^ Except for the terms in eq 6 that encompass the full-length FH fragments, the sine terms are small for Ψ angles negative and small in absolute value. (After adding a γ corresponding to the inside of a helical segment the argument of the sine term becomes small.) However, because there are many shorter fragments of a given FH segment, the contributions from the shorter fragments, especially those with one end corresponding to the end of a segment and the other one buried inside the helix, are dominant.

The above considerations are well illustrated with the $V_{\mathrm{mtor};n}^{f}$ maps in γ_N_ and γ_C_ for the three sets of parameters determined by the maximum likelihood method (Table 1) for AA-type pair (the most frequently occurring in proteins) shown in Figure 4A–C. Additionally, the map of the statistical PMF calculated from the distribution of γ_N_ and γ_C_ angles determined from the PDB in our earlier work^42^ is shown in Figure 4D. The plots for the other residue-type pairs are shown in Figures S1–S3. When constructing the $V_{\mathrm{mtor};n}^{f}$ maps, the segment length was set at *n* = 20, all θ angles were set to 90°, and the γ angles inside the helical section of the studied segment (i.e., apart from its ends) were set at 45°. It should be noted that most of the contributions to the maps of $V_{\mathrm{mtor};n}^{f}$ plots shown in Figure 4A–C arise from fragments shorter that 20 residues, with one end exposed and the other one buried inside the helical segment.

**Figure 4:**
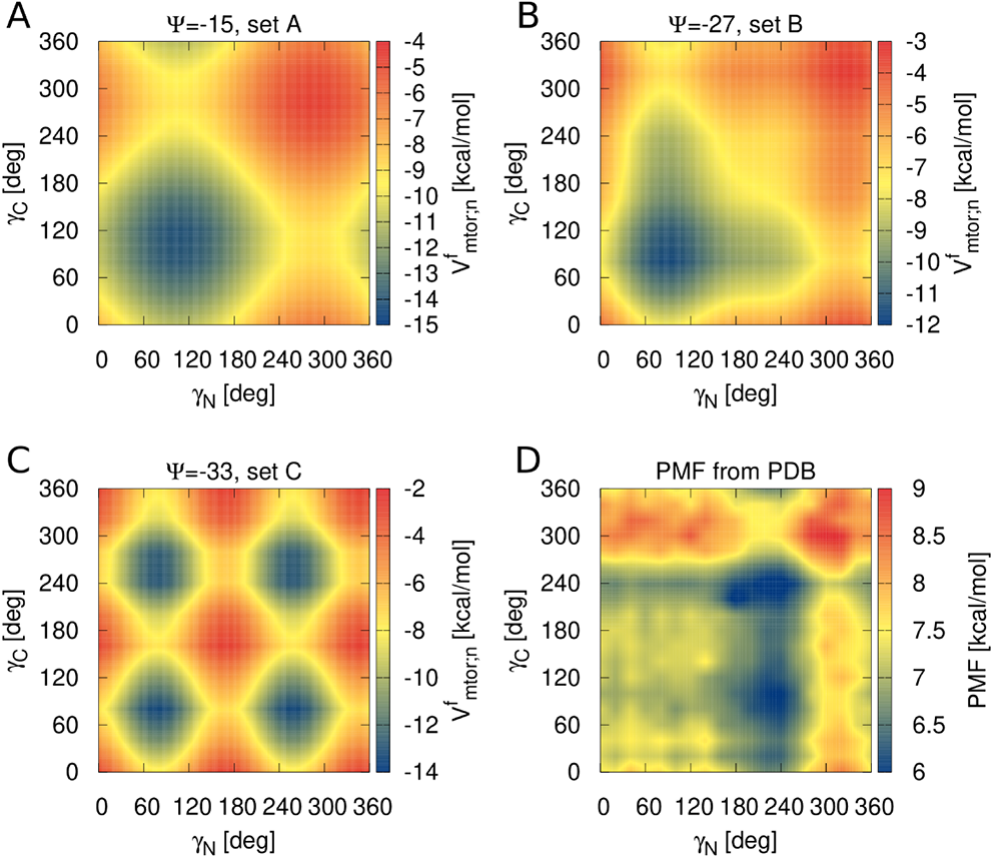
Heat maps of $V_{\mathrm{mtor};n}^{f}$ (eq 6) in the terminal virtual-bond dihedral angles γ_N_ and γ_C_ (see Figure 2 for illustration) of a 20-residue FH segment obtained with set A (A), set B (B) and set C (C) parameters of the multitorsional potentials (Table 1) for the alanine-type sequences and the statistical PMF (D) calculated from the distribution of γ_N_ and γ_C_ determined in our earlier work.^42^ The plots were made with gnuplot.^51^

The PMF determined from the PDB (Figure 4D) has a broad low-energy basin around γ_N_ = 240° with one minimum at around (γ_N_ = 240°, γ_C_ = 240°) and (γ_N_ = 240°, γ_C_ = 90°) , respectively. These minima reflect the maxima in the respective distributions of γ_N_ and γ_C_ reported in our earlier work.^42^ The map of $V_{\mathrm{mtor};20}^{f}$ obtained with set C of parameters (*M* = 2 only; Figure 4C), has the low-energy region at γ_N_=240° with two mimina close to those of those in the PMF; however, this low-energy region is repeated at γ_N_ close to 60^◦^, which is less pronounced in the PMF. For set A (*M* = 1; Figure 4A), the only minimum is located at γ_N_ ≈ 90° and γ_C_ ≈ 90° and its basin extends to γ_N_ = 240°, thus partially capturing the features of the distribution from the PDB.^42^ However, it has a maximum at both γ_N_ and γ_C_ close to 240°. Set B, with both *M* = 1 and *M* = 2 (Figure 4B), has all the minima close to those present in the PMF; however, with the most pronounced one at γ_N_ ≈ 90° and γ_C_ ≈ 90°. It should be noted, though, that the distribution of the (γ_N_, γ_C_) pairs is not the only manifestation of the multitorsional terms, which also account for stabilizing the α-helical portion inside the segment. Thus, comparing the maps of different variants of $V_{\mathrm{mtor};n}^{f}$ in (γ_N_, γ_C_) with the respective statistical PMF determined from the PDB does not enable us to fully judge the quality of these variants. The real test of UNRES with these variants is *ab initio* protein-structure modeling, which is described in Section 3.3.

To get more insight into the behavior of $V_{\mathrm{mtor};20}^{f}$ , we computed the respective surfaces for more values of the phase angle Ψ for both *M* = 1 and *M* = 2. The plots are shown in Figure S4. It can be seen that changing the value of Ψ results in shifting the positions of minima. Some values of Ψ result in nearly flat surfaces. On the other hand, Ψ = 150° seems to yield surfaces with minima in good positions. However, as mentioned above, large values of Ψ make the cosine term inside $V_{\mathrm{mtor};20}^{f}$ change sign to negative and, thus the whole term heavily depends on fragment length. Consequently, the values determined by maximum-likelihood method seem to be the most plausible ones. For this reason, we tried the three sets of parameters collected in Table 1 on the set of 28 benchmark proteins listed in Section 2.4.

### 3.2 Stabilization of folded-Helical Segments by Multitorsional Terms

To find out how do the multitorsional potentials contribute to the stabilization of the FH segments in the context of the entire structure, we calculated all $U_{\mathrm{mtor};i;m}^{f}$ components of these potentials (eq 3) for the experimental structure of 1K40 protein (126 residues). The structure of this protein is a four-helix bundle with long α-helices spreading from N921(1) to Q943(23) (helix I; 23 residues), from Y950(30) to I972(52) (helix II; 23 residues), from T979(59) to Y1007(97) (helix III; 29 residues) and from M1009(89) to K1044(124) (helix IV; 36 residues), respectively, where the number following the one-letter symbol of a residue is its index in the PDB structure and that in parentheses is the index relative to the first residue in the structure (N921). Additionally, small helices composed of approximately a single turn each occur between helix I and helix II from P946(26) to E949(29) (4 residues) and between helix II and helix III from P973(53) to L975(55) (3 residues), respectively.

The heat maps of $U_{\mathrm{mtor};i;m}^{f}$ components for parameter set B, which implies the presence of terms with both *M* = 1 and *M* = 2 (cf. Section 3.1) plotted in the index of the first γ angle (*i*) and segment length (*m*) are shown in Figure 5. The maps obtained with parameter sets A (*M* = 1 only) and C (*M* = 2 only) are similar. As can be seen from the figure, the helical segments are clearly distinguished in the map as regions of low $U_{\mathrm{mtor};i;m}^{f}$ components, without auxiliary information from secondary-structure prediction methods. The two small helices between helix I and helix II and helix II and helix III are also seen in the map. The green vertical patch corresponding to positive (destabilizing) $U_{\mathrm{mtor};i;m}^{f}$ components at helix II corresponds to proline at position 952(32), which agrees with the fact that proline is a helix breaker. The upper-triangular white or lightly green (zero or slightly positive $U_{\mathrm{mtor};i;m}^{f}s$ ) sections of the heat map correspond to those $U_{\mathrm{mtor};i;m}^{f}s$ that encompass a helix boundary inside, thus rendering the respective $U_{\mathrm{mtor};i;m}^{f}s$ negligible or destabilizing. This result conforms with the observation made in Section 3.1 that the $U_{\mathrm{mtor};i;m}^{f}$ terms stabilize α-helices. It can also be seen that the contributions to $U_{\mathrm{mtor};i;m}^{f}s$ decay with segment length (*m*).

**Figure 5:**
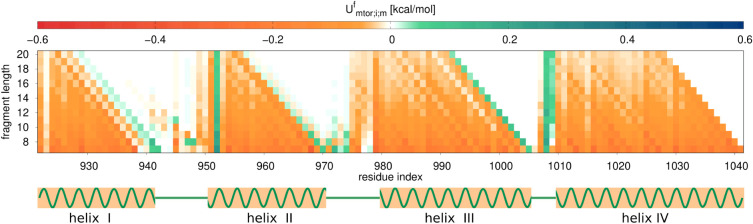
Heat map of $U_{\mathrm{mtor};i;m}^{f}$ (eq 3) for the experimental structure of 1K40 in residue index corresponding to a given backbone-virtual-bond dihedral angle γ (*i*) and fragment length starting at this residue (*m*) for parameter set B (Table 1). The plots were made with gnuplot.^51^

### 3.3 Tests of UNRES with Multitorsional Terms

We have tested the performance of UNRES augmented with the $U_{\mathrm{mtor}}^{f}$ terms (eq 4), terms for the 28 benchmark α-helical proteins, as described in Section 2.4. We tried all three parameter sets A, B, and C (Table 1) of the multitorsional potential. For reference, we also carried out calculations without multitorsional terms, using the NEWCT-9P (state-of-the-art) UNRES,^44^ also referred to as UNRES with *w*_mtor_ = 0.0 (eq 5) calculations. For three multitorsional-potential parameter sets, we tried *w*_mtor_ of 0.10, 0.15, and 0.20. The magnitude of the multitorsional-term weight is small because the multitorsional term was added in this work to the UNRES energy function without changing the other components. A greater contribution from the multitorsional potential would require recalibration of the whole UNRES energy function. However, the purpose of this work was to find out how the multitorsional potential affects the modeled structures without changing the other components of the force field.

Together with those performed using state-of-the-art UNRES, we carried out 10 sets of calculations. The GDT_TS and its differences from the values corresponding to state-of-the-art UNRES for the first models (with top probability) and the best models (models with the highest GDT_TS) are shown in Figures 6 and 7, respectively. The numerical values of GDT_TS as well as those of RMSD and TM-score are collected in Tables S1–S10.

**Figure 6:**
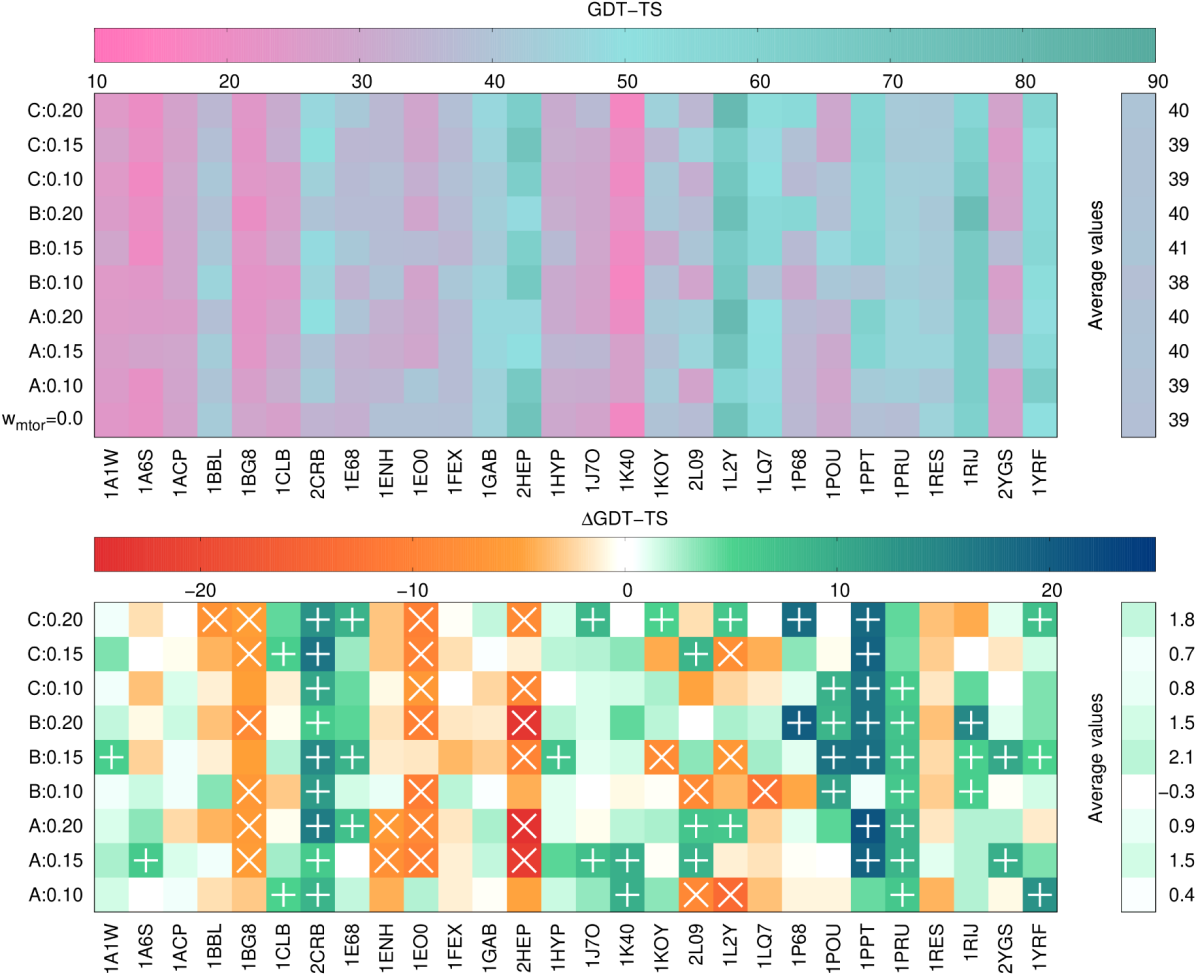
GDT_TS values (top panel) and differences from the GDT_TS corresponding to state-of-the-art UNRES calculations (ΔGDT_TS) of the first models of the 28 benchmark proteins. The GDT_TS and ΔGDT_TS color scales are on the top of each panel. Rows correspond to the type of calculation (state-of-the-art UNRES at the bottom of the top panel), with multitorsional-potential parameter sets A, B, and C, and multitorsional-potential weights indicated. The right bars correspond to values averaged over all benchmark proteins. The “+” marks in the bottom panel indicate ΔGDT_TS higher that 5 units, while the “×” marks indicate ΔGDT_TS lower than −5 units. The plots were made with gnuplot.^51^

**Figure 7:**
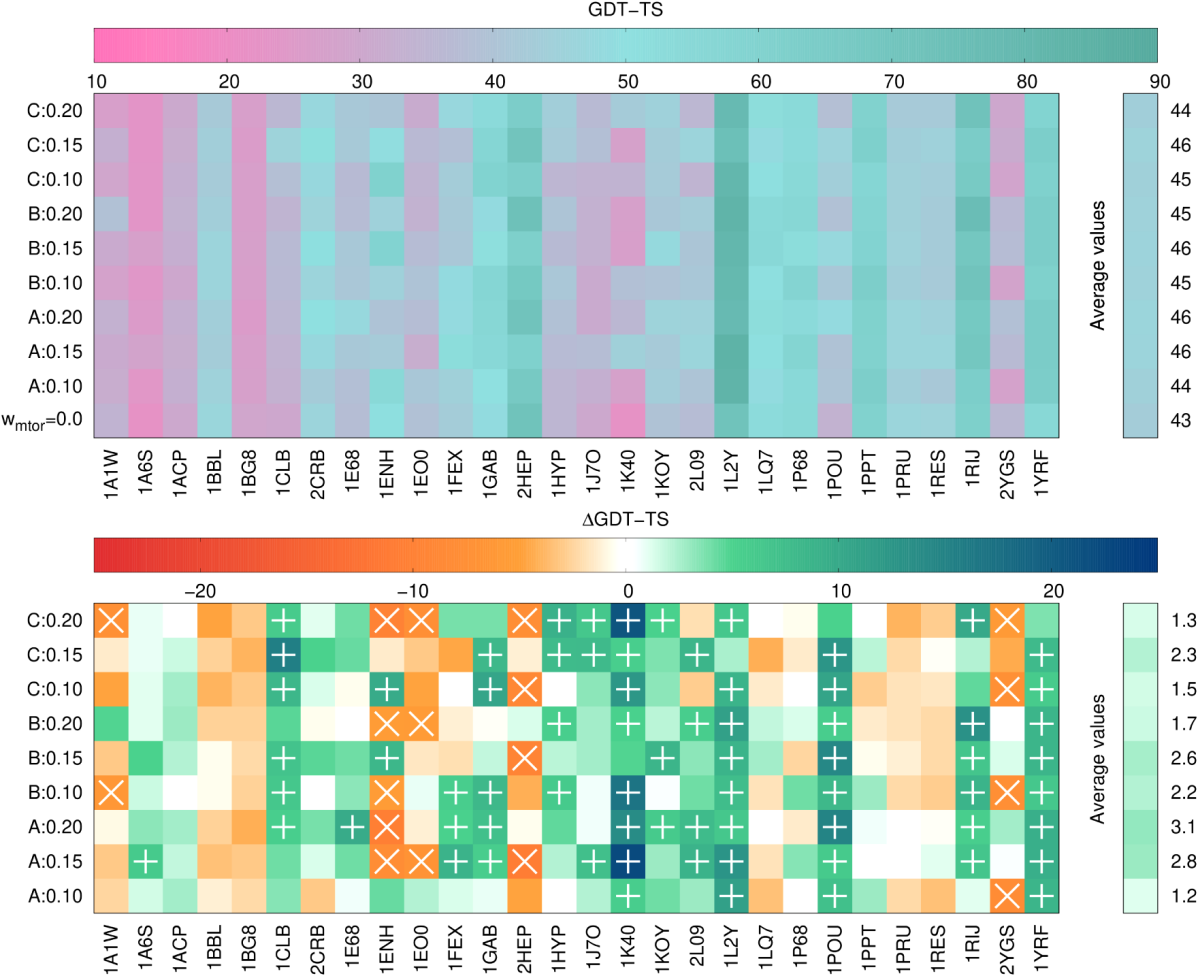
GDT_TS values (top panel) and differences from the GDT_TS corresponding to state-of-the-art UNRES calculations (ΔGDT_TS) of the best models of the 28 benchmark proteins. The GDT_TS and ΔGDT_TS color scales are on the top of each panel. Rows correspond to type of calculation (state-of-the-art UNRES at the bottom of the upper panel), with multitorsional-potential parameter sets A, B, and C, and multitorsional-potential weights indicated. The right bars correspond to values averaged over all benchmark proteins. The “+” marks in the bottom panel indicate ΔGDT_TS higher that 5 units, while the “×” marks indicate ΔGDT_TS lower than −5 units. The plots were made with gnuplot.^51^

As can be seen from Figures 6 and 7, GDT_TS increases on average with respect to state-of-the-art UNRES for both the first and the best model except for parameter set B with *w*_mtor_ = 0.1. This observation strongly suggests that the multitorsional term improves the quality of UNRES models of α-helical proteins. On the other hand, the average increase is rather moderate, which suggests that further tuning of the multitorsional potential is required. It can also be seen that parameter set B with *w*_mtor_ = 0.15 consistently results in the increase of average GDT_TS for both the first and the best model. This parameter set also results in the highest number of first models with ΔGDT_TS > 5 (10) and only 3 first models with ΔGDT_TS < -5. The multitorsional term with parameter set B and weight of 0.15 is, therefore, recommended to be used in the calculations. On the other hand, parameter set A with *w*_mtor_ = 0.2 results in the greatest increase of the GDT_TS on average for the best model and for the largest number of best models (11) with ΔGDT_TS > 5 with only one best model with ΔGDT_TS < -5.

The first and best models of all 28 benchmark proteins superposed on the respective experimental structures, obtained in calculations with state-of-the-art UNRES, with parameter set A, *w*_mtor_ = 0.2, and parameter set B, *w*_mtor_ = 0.15 are shown in Figures S5–S32. Here we discuss in detail three cases: 1K40, 1POU and 1ENH. For 1K40 and 1POU introducing the multitorsional terms results mostly in the improvement of the models while for 1ENH mostly in deterioration (Figures 6 and 7). 1K40 is not modeled well with state-of-the-art UNRES probably because of long helices that are not stable enough. 1POU contains shorter helices but more complex packing topology and is not modeled by state-of-the-art UNRES well, though better than 1K40. 1ENH has a simpler topology and is modeled fairly well with state-of-the-art UNRES.

The best models of 1K40 superposed on the experimental structure are shown in Figure 8A. With state-of-the-art UNRES, the model is of poor quality, with α-helices remarkably shorter than in the experimental structure. Some quantitative improvement is observed for UNRES augmented with multitorsional potentials with parameter set B. Major quantitative and qualitative improvement is observed with parameter set A, with which a slightly distorted 4-helix bundle is formed. The best model, with RMSD of 5.19 Å, is obtained with parameter set A and *w*_mtor_ = 0.15, which generally does not give the best results. It should be noted that with all previous versions of UNRES, including the one that was optimized only with α-helical proteins,^70^ 1K40 was modeled poorly and restraints, such as from simulated cross-linking experiments,^71^ were required to obtain reasonable models.

**Figure 8:**
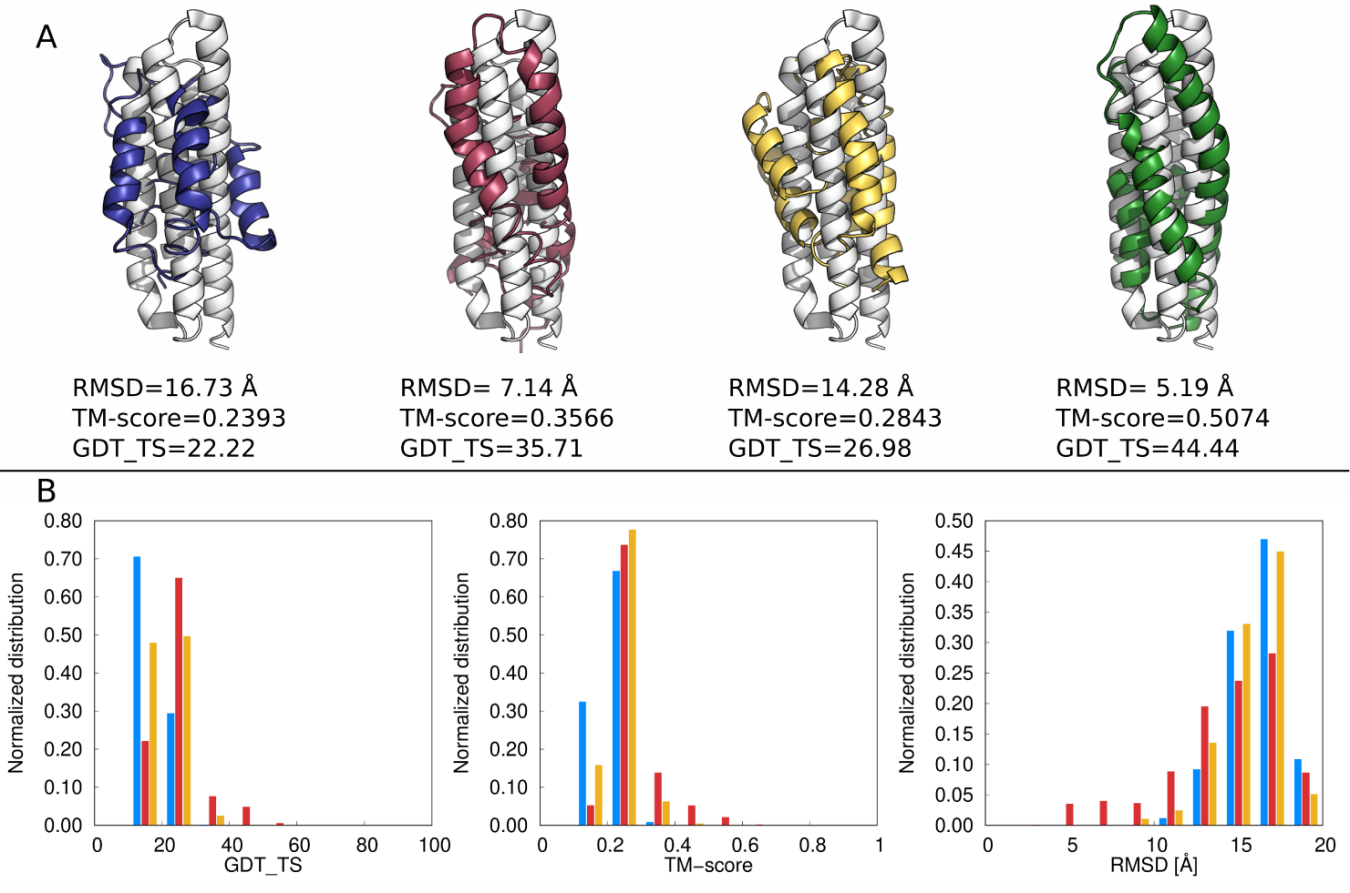
(A) The best models of 1K40 obtained with state-of-the-art UNRES (blue), multitorsional potential with parameter set A, *w*_mtor_ = 0.2 (red),multitorsional potential with parameter set B, *w*_mtor_ = 0.15 (yellow), multitorsional potential with parameter set A, *w*_mtor_ = 0.15 (green) superposed on the experimental structure (white). The GDT_TS, TM-score, and RMSD values are shown. (B) Distributions and GDT_TS, TM-score, and RMSD obtained from the 3,024 frames of a given MREMD simulation after processing with WHAM and using WHAM-calculated probabilities at *T* = 300 K. Blue bars correspond to state-of-the-art UNRES, red solid bars correspond to parameter set A, *w*_mtor_ = 0.2, and yellow bars correspond to parameter set B, *w*_mtor_ = 0.15. The structures were drawn with PyMOL^72^ and the plots were made with gnuplot.^51^

The improvement is not only the feature of the best model but of the whole distribution of modeled structures. The distributions of GDT_TS, TM-score, and RMSD calculated from the frames from MREMD processed with WHAM and using WHAM-determined weights at *T* = 300 K, are shown in Figure 8B. It can be seen that, with the multitorsional term, the maxima of GDT_TS and TM-score distributions shift to the right and the tails extend to the right. The change is more pronounced for parameter set A, *w*_mtor_ = 0.2. The maxima of RMSD distributions do not shift to the left but the tails extend to the left.

To find out the origin of the improvement of the models, we constructed the plots of the distributions of GDT_TS, TM-score, and RMSD for the sections of the protein which correspond to α-helices and the joining loops with the adjacent helix turns in the experimental 1K40 structure. The plots are shown in Figure 9. It can be seen that, for all sections, the GDT_TS and TM-score distributions are shifted to the right. The shift is more pronounced for the α-helical segments but is also remarkable for the loop segments. This observation suggests that the multitorsional term strengthens the helices and improves the geometry of the intervening regions as postulated in our earlier work.^42^ The RMSD distribution is more remarkably left-shifted for α-helical regions but is also left-shifted or at least increased at lower RMSD values for the loop regions. Without the multitorsional term, 4 short helices are formed with large intervening loops, this resulting in incorrect packing (Figure 8A).

**Figure 9:**
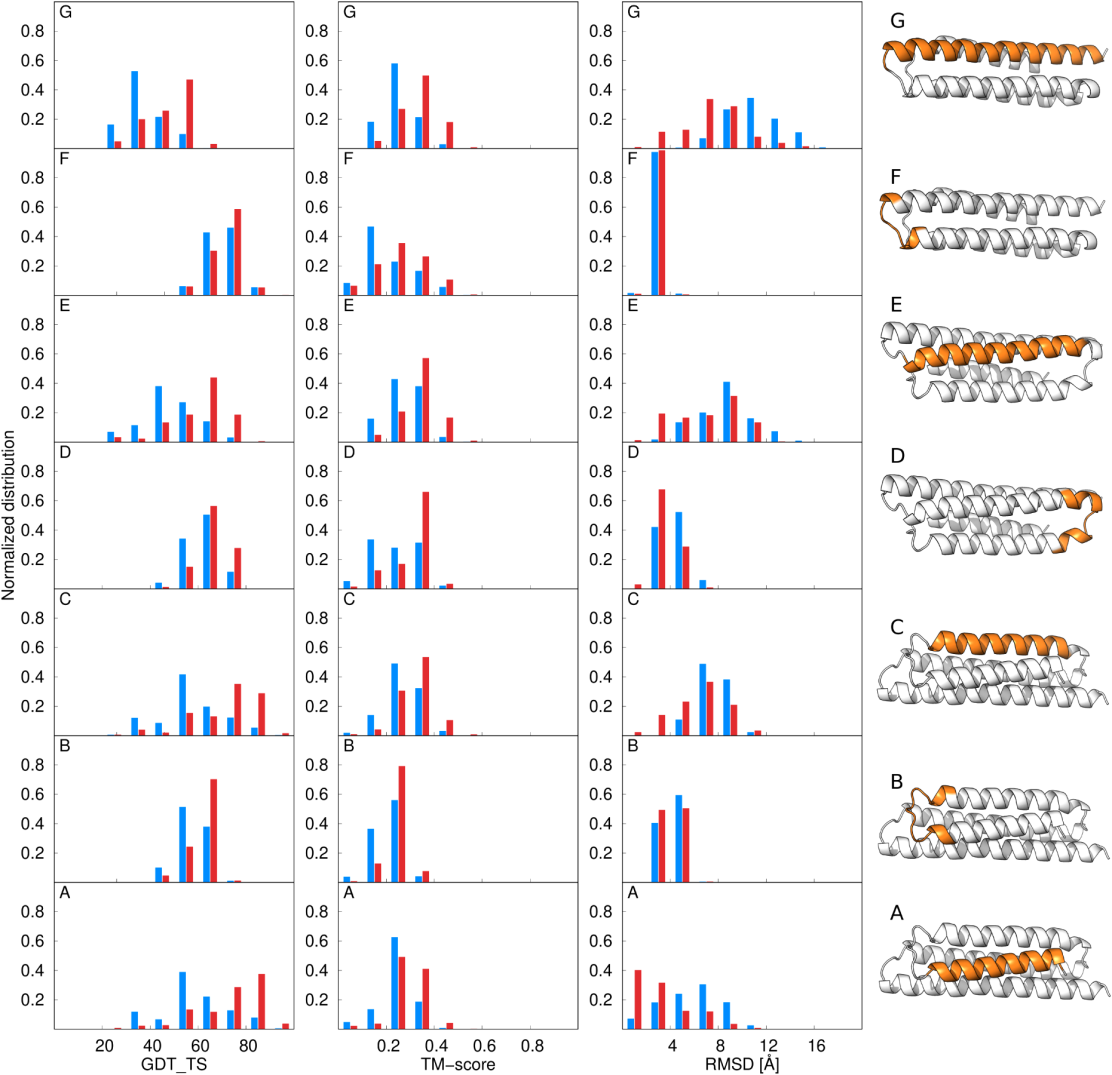
Distributions of GDT_TS, TM-score, and RMSD calculated for the consecutive sections of the models of 1K40 corresponding to α-helical and loop sections of its experimental structure. Blue bars correspond to state-of-the-art UNRES, red bars correspond to parameter set A, *w*_mtor_ = 0.2. The structures were drawn with PyMOL^72^ and the plots were made with gnuplot.^51^

The best models of 1POU superposed on the experimental structure are shown in Figure 10A. Augmentation of UNRES with the multitorsional potential results in the improvement of the best model in terms of RMSD, GDT_TS, and TM-score, for both parameter set A, *w*_mtor_ = 0.2 and set B, *w*_mtor_ = 0.15. The GDT_TS increases by about 15 units and RMSD drops from 9.64  Å to 5.25  Å for parameter set B. In particular, the N-terminal α-helix of the best state-of-the-art UNRES model is misaligned with that of the experimental structure, while it is almost perfectly aligned in both models obtained with augmented UNRES.

**Figure 10:**
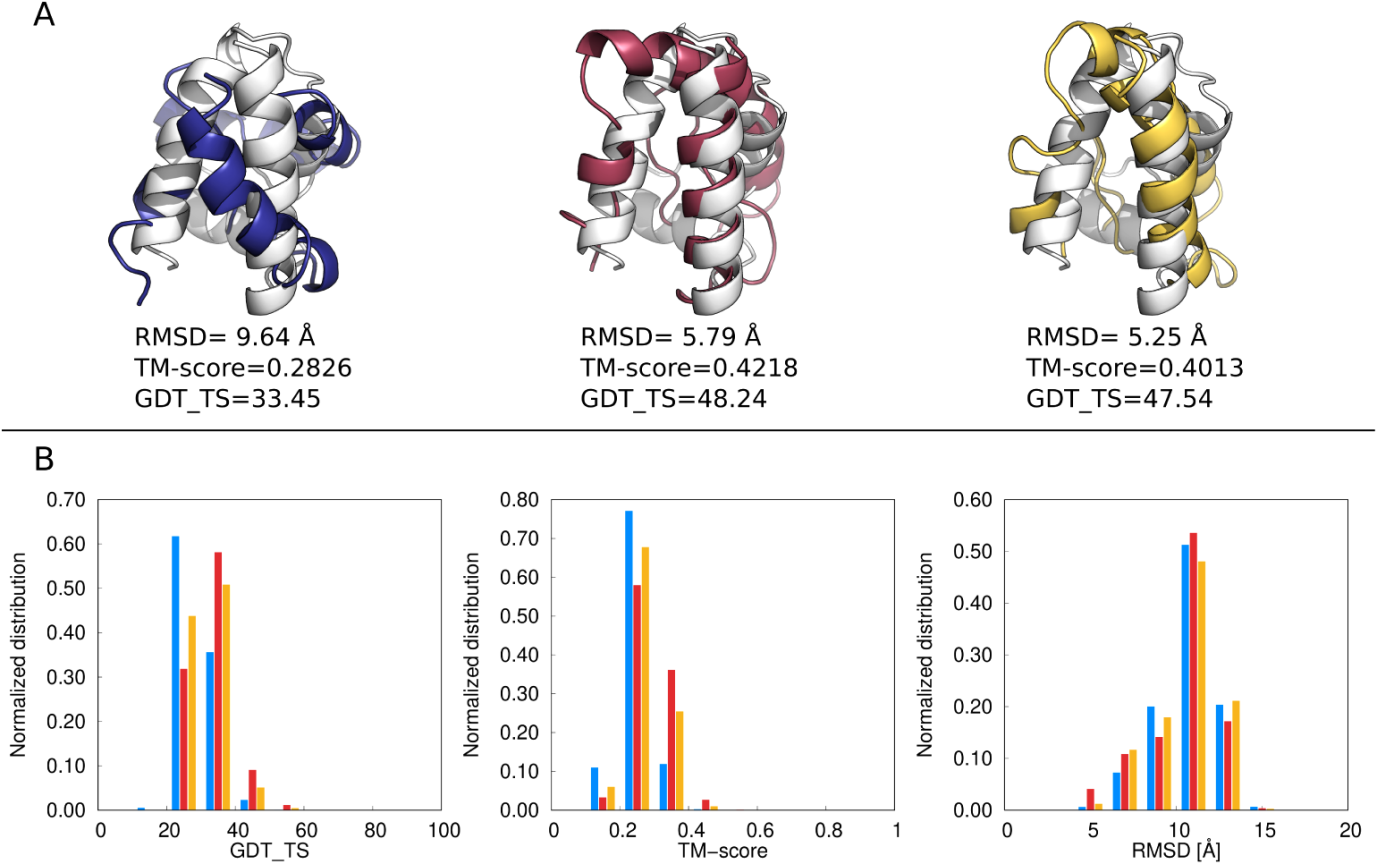
(A) The best models of 1POU obtained with state-of-the-art UNRES (blue), multitorsional potential with parameter set A, *w*_mtor_ = 0.2 (red),multitorsional potential with parameter set B, *w*_mtor_ = 0.15 (yellow) superposed on the experimental structure (white). The GDT_TS, TM-score, and RMSD values are shown. (B) Distributions of GDT_TS, TM-score and RMSD obtained from the 3,024 frames of a given MREMD simulation after processing with WHAM and using WHAM-calculated probabilities at *T* = 300 K. Blue bars correspond to state-of-the-art UNRES, red bars correspond to parameter set A, *w*_mtor_ = 0.2, and yellow bars correspond to parameter set B, *w*_mtor_ = 0.15. The structures were drawn with PyMOL^72^ and the plots were made with gnuplot.^51^

The distributions of GDT_TS, TM-score, and RMSD are presented in Figure 10B. The introduction of the multitorsional potential results in the shift of the GDT_TS maximum to the right, and in the increase of the TM-score distribution at higher values. In addition, there is a slight increase of RMSD distribution at low RMSD values. Thus, structural improvements observed for the best models of 1POU are reflected in the distributions of the three similarity measures.

The best models of 1ENH superposed on the experimental structure are shown in Figure 11A. The best model generated by the state-of-the-art UNRES is in good agreement with the experimental structure in terms of RMSD, TM-score and GDT_TS metrics. The C-terminal α-helix of the model is somehow misaligned with that of the experimental structure. The introduction of multitorsional potential applying parameter set A, *w*_mtor_ = 0.20 leads to merging the N-terminal α-helix with the second one, which results in deterioration of model quality in terms of RMSD, GDT_TS, and TM-score. However, the quality of the best model is improved with respect to state-of-the-art UNRES with parameter set B, *w*_mtor_ = 0.15. On the other hand, the distributions of GDT_TS, TM-score, and RMSD (Figure 11B) indicate that, for this protein, UNRES with multitorsional terms performs worse than state-of-the-art UNRES for both parameter sets. In particular, RMSD distributions are remarkably shifted to the right. The RMSD distribution at lower RMSD values is more pronounced for parameter set B, which could result in surfacing of the model with a relatively low RMSD in cluster analysis.

**Figure 11:**
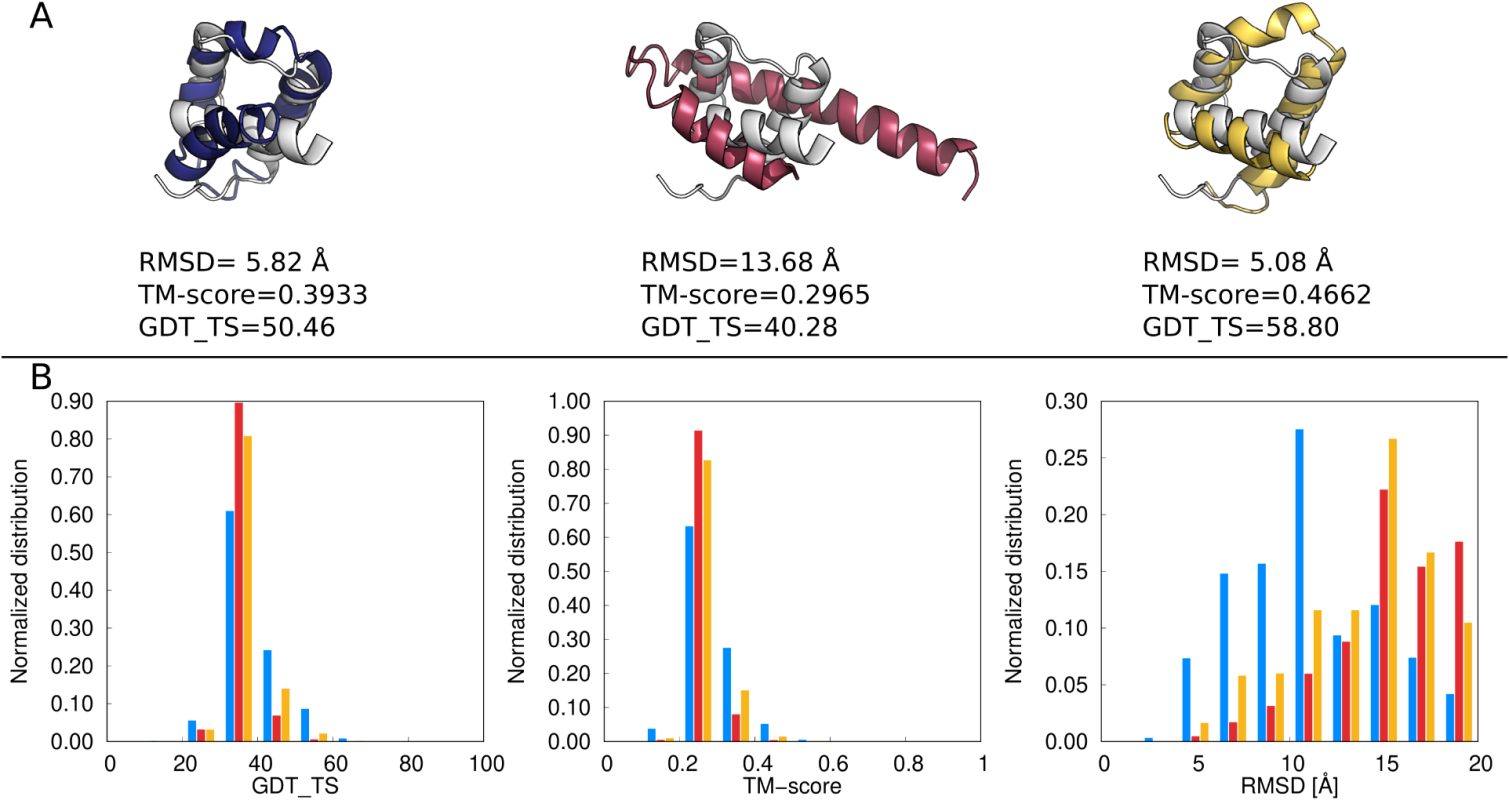
(A) The best models of 1ENH obtained with state-of-the-art UNRES (blue), multitorsional potential with parameter set A, *w*_mtor_ = 0.2 (red),multitorsional potential with parameter set B, *w*_mtor_ = 0.15 (yellow) superposed on the experimental structure (white). The GDT_TS, TM-score, and RMSD values are shown. (B) Distribution of GDT_TS, TM-score, and RMSD obtained from the 3,024 frames of a given MREMD simulation after processing with WHAM and using WHAM-calculated probabilities at *T* = 300 K. Blue bars correspond to state-of-the-art UNRES, red bars correspond to parameter set A, *w*_mtor_ = 0.2, and yellow bars correspond to parameter set B, *w*_mtor_ = 0.15. The structures were drawn with PyMOL^72^ and the plots were made with gnuplot.^51^

## 4 Conclusions

In this work we parametrized and implemented the multitorsional potentials, $U_{mtor}^{f}$ (eq 3), proposed in our earlier work^42^ that describe cooperative interactions extending to larger chain segments in the UNRES coarse grained model of proteins. In our earlier work^42^ we suggested that these potentials could help to model α-helical proteins better. Simulations with the proposed helix-promoting multibody terms do not require prior secondary-structure information. Consistent with handling local interactions in UNRES^22,23^ we developed parameters for nine pairs of three basic types of amino-acid residues, namely glycine, alanine and proline, alanine representing all residue types except for glycine and proline. Three sets of parameters were selected and UNRES augmented with the multitorsional terms with different weights was tested with the set of 28 α-helical proteins that were not used in its parametrization and were used to test the previous versions of the force field.^44^ No parameters of state-of-the-art UNRES were optimized.

The best consensus improvement of the quality of modeled structures was obtained with the multitorsional potentials containing terms with both multiplicity *M* = 1 and *M* = 2 [eq 3]. This set of parameters resulted in the most pronounced improvement on average of the first models (those with the top probability) and remarkable improvement of the best models, in terms of the GDT_TS similarity measure. However, the potentials with *M* = 1 alone resulted in the most remarkable improvement of the best models. The most significant improvement (by more that 20 GDT_TS units) was obtained for the 1K40 protein (Figure 8). The structure of this protein has never been predicted correctly by the state-of-the-art and previous versions of UNRES despite a simple sequential 4-helix-bundle topology. This improvement was caused by both enhancing α-helical segments, which are relatively long and are poorly formed with state-of-the-art UNRES, and improving the geometry of the linking loops, which resulted in proper packing of the α-helices (Figure 9), consistent with the predictions of the features of the multitorsional potentials of the FH segments of our earlier work. It should be stressed that the new multitorsional terms are imposed on all the segments of the chain, without having to input their secondary structure. The multitorsional potentials spontaneously take negative values for folded-helical segments, while turning nearly zero or slightly positive when there is an intervening loop between two helices in the fragment under consideration (Figure 5). In some instances, such as 1ENH, the helix-promoting property of the new multitorsional potential can, however, result in merging α-helices which are separate in the experimental structure, thus deteriorating model quality (Figure 11).

The net positive impact of the $U_{\mathrm{mtor}}^{f}$ potentials on the quality of the models of α-helical proteins obtained with UNRES seems to be due to their capturing the cooperativity of helix formation. This cooperativity is implicitly present when using all-atom treatment (which is, however, not feasible with larger proteins) but apparently lost when coarse-graining the representation.^24^ In the state-of-the-art UNRES, only up to third-order correlations are present, which enables us to achieve a good model quality in general but appears to be insufficient for long α-helical segments or more complex helix-packing topologies. Even though α-helices are relatively simple secondary-structure elements whose formation is governed by local interactions, still the process of helix formation is highly cooperative.^28^

It should be noted that full implementation of the $U_{\mathrm{mtor}}^{f}$ potentials requires optimization of other UNRES parameters, in particular the weights of the energy terms. Moreover, the multitorsional potentials promoting extended strands and directing their ends, $U_{\mathrm{mtor}}^{e}$ proposed in our earlier work^42^ also need to be introduced to handle sections with β-sheet structure. These potentials can also contribute to handling the loop regions, including those between α-helices better and could also prevent from overstretching α-helices, which was observed for 1ENH (Figure 11). This work is now underway in our laboratory. In the future, we plan to extend the work on long-range cooperative terms to the coupling of hydrogen-bonding and local interactions, i.e., beyond the present third-order $U_{\mathrm{corr}}^{\left(3\right)}$ terms of the present UNRES. Following the recent successful example of using machine learning to identify important multibody terms,^73^ we also intend to empower the scale-consistent theory of coarse graining^24^ with machine-learning tools to find the correlation terms that are essential to describe protein free-energy landscapes at the coarse-grained level.

## Supplementary Material



## Notes

The optimized expressions for the multitorsional potentials and their gradients; Figures S1–S3: heat maps of $V_{\mathrm{mtor};n}^{f}$ (eq 6) in the terminal virtual-bond dihedral angles γ_*N*_ and γ_*C*_ of a 20-residue FH segment obtained with parameter sets A, B, C; Figure S4: heat maps of $V_{\mathrm{mtor};20}^{f}$ (eq 6) of a 20-residue FH segment in the terminal γ_N_ and γ_C_ angles and various phase angles Ψ; Figures S5–S32: the first models (top-probability) and the best models (with the highest GDT_TS) obtained with plain UNRES, multitorsional potential with parameter set A, *w*_mtor_ = 0.2, multitorsional potential with parameter set B, *w*_mtor_ = 0.15 superposed on the experimental structure (white), with corresponding GDT_TS, TM-score, and RMSD values, for the structures of the benchmark proteins; Tables S1–S10: characteristics (probability, RMSD, TM-score, and GDT_TS) of the first (top-probability) and measure-best models of the structures of the benchmark proteins obtained using UNRES with multitorsional potential (PDF)
