# Supplementary material for: Recapturing Cooperativity of α‑Helix Formation and Packing in Coarse-Grained Protein Structure Modeling with Multitorsional Potentials
Source: J Phys Chem B. 2025 Jul 3;129(28):7119–33. doi: 10.1021/acs.jpcb.5c03985 (PMC12278218; doi:10.1021/acs.jpcb.5c03985)
Supplement: Supplementary file 1 [file jp5c03985_si_001.pdf]

Supporting Information: Recapturing Cooperativity of α-Helix Formation and Packing in Coarse-Grained Protein Structure Modeling with Multitorsional Potentials

Elizaveta F. Petrusevich and Adam Liwo*

Faculty of Chemistry, University of Gdańsk, Fahrenheit Union of Universities in Gdańsk, Wita Stwosza 63, 80-308 Gdańsk, Poland

E-mail: adam.liwo@ug.edu.pl

Optimized Expressions for the Multitorsional Potentials and Their Gradients

For the sake of completeness, in this section we present the transformation of the basic expressions for multitorsional potentials [Equations (3) and (4) of the main text] and the formulas for the gradients of these terms.

After reorganizing, Equation (3) of the main text , the the multitorsional-energy term corresponding to a m-residue segment starting at the ith residue is expressed by Equation (S1).

$$
\begin{aligned}
U^{f}_{mtor;i;m} &= \sum_{M=1}^{2} w_M (\sin\theta_{i+1}\sin\theta_{i+2})^M \sin[M(\gamma_{i+1}+\Psi_{i+1})] \prod_{k=i+2}^{i+m-4} (\sin\theta_k \sin\theta_{k+1})^M \\
&\quad \times \cos[M(\gamma_k+\Psi_k)](\sin\theta_{i+m-3}\sin\theta_{i+m-2})^M \sin[M(\gamma_{i+m-3}+\Psi_{i+m-3})] \\
&= \sum_{M=1}^{2} w_M \Big[(\sin\theta_{i+1}\sin\theta_{i+2})^M [\sin(M\gamma_{i+1})\cos(M\Psi_{i+1}) \\
&\qquad\qquad + \cos(M\gamma_{i+1})\sin(M\Psi_{i+1})]\Big] \\
&\quad \times \left\{ \prod_{k=i+2}^{i+m-4} (\sin\theta_k\sin\theta_{k+1})^M [\cos(M\gamma_k)\cos(M\Psi_k) - \sin(M\gamma_k)\sin(M\Psi_k)] \right\} \\
&\quad \times (\sin\theta_{i+m-3}\sin\theta_{i+m-2})^M [\sin(M\gamma_{i+m-3})\cos(M\Psi_{i+m-3}) \\
&\qquad\qquad + \cos(M\gamma_{i+m-3})\sin(M\Psi_{i+m-3})]
\end{aligned}
\tag{S1}
$$

where M is the multiplicity of a term, w_M is the weight of the term with a given multiplicity, θs and γs are the backbone-virtual-bond angles and the backbone-virtual-bond dihedral angles, respectively, defined in Figure 1 of the main text, and the Ψs are the phase angles that depend on the kinds of residues.

Following our previous work,[S1] we introduce new variables, encompassing virtual-bond

angles θ and dihedral angles γ [Equation (S2)]. These quantities are defined solely in terms of the scalar and vectorial products of the $C^{\alpha} \cdots C^{\alpha}$ virtual-bond vectors that define the respective dihedral angle and no computation of actual angles is needed, which prevents from singularities that could arise when the respective 4-residue fragment is close to linear.

$$\phi_i^{(M)} = (\sin\theta_i \sin\theta_{i+1})^M \cos(M\gamma_i) \tag{S2}$$

$$\psi_i^{(M)} = (\sin\theta_i \sin\theta_{i+1})^M \sin(M\gamma_i) \tag{S3}$$

With these definitions, the complete expression for the multitorsional energy of a polypeptide chain with n_{res} residues is given by Equation (S4).

$$\begin{aligned} U_{mtor}^f = \sum_{m=7}^{m_{max}} \sum_{i=1}^{n_{res}-m+1} \sum_{M} w_M \left[\cos(M\Psi_{i+1})\psi_{i+1}^{(M)} + \sin(M\Psi_{i+1})\phi_{i+1}^{(M)}\right] \\ \times \prod_{k=i+2}^{i+m-4} \left[\cos(M\Psi_k)\phi_k^{(M)} - \sin(M\Psi_k)\psi_k^{(M)}\right] \\ \times \left[\cos(M\Psi_{i+m-3})\psi_{i+m-3}^{(M)} + \sin(M\Psi_{i+m-3})\phi_{i+m-3}^{(M)}\right] \end{aligned} \tag{S4}$$

The quantities ψ_i^n, ϕ_i^n and their gradients in the C^{α} Cartesian coordinates are computed using the modified Tschebyshev polynomial described previously.[S1]

To optimize the calculations of U_{mtor}^f, we define the quantities $B_{m,i,M}$, $7 \leq m \leq n_{res}-i-4$, in a recursive manner, as given by Equations (S5) and (S6), respectively (we recall that $m = 7$ is the length of the shortest chain section for which the multitorsional contributions are considered).

$$B_{m,i,M} = B_{m-1,i,M}\left[\cos(M\Psi_{i+m-4})\phi_{i+m-4}^{(M)} - \sin(M\Psi_{i+m-4})\psi_{i+m-4}^{(M)}\right] \tag{S5}$$

$$B_{7,i,M} = \left[\cos(M\Psi_{i+1})\psi_{i+1}^{(M)} + \sin(M\Psi_{i+1})\phi_{i+1}^{(M)}\right]\left[\cos(M\Psi_{i+2})\phi_{i+2}^{(M)} - \sin(M\Psi_{i+2})\psi_{i+2}^{(M)}\right]$$
$$\times\left[\cos(M\Psi_{i+3})\phi_{i+3}^{(M)} - \sin(M\Psi_{i+3})\psi_{i+3}^{(M)}\right] \tag{S6}$$

With these definitions, $U^f_{mtor;i;m;F}$ is expressed by Equation (S7).

$$U^f_{mtor;i;m;M} = -w_M \times B_{m,i,M} \times \left[\cos(M\Psi_{i+m-3})\psi_{i+m-3}^{(M)} + \sin(M\Psi_{i+m-3})\phi_{i+m-3}^{(M)}\right] \tag{S7}$$

The gradient of U_{mtor} is also computed using the above recursive scheme. The gradient is defined in virtual-bond vectors defined by Equation (S8).

$$\mathbf{dC}_k = \mathbf{C}_{k+1} - \mathbf{C}_k,\ k = 1, 2, \ldots, n_{res-1} \tag{S8}$$

where $\mathbf{C}_k$ denotes the Cartesian coordinates of the C$^{\alpha}$ atom of the kth residue.

The derivatives of the quantity $B_{7,i,M}$ in virtual-bond vectors $\mathbf{dC}_k$, are expressed by Equations (S9)–(S15)

$$\nabla_{\mathbf{dC}_{i-1}} B_{7,i,M} = 0 \tag{S9}$$

$$
\begin{aligned}
\nabla_{\mathbf{dC}_i} B_{7,i,M} = &\left[\cos[M\Psi_{i+1}]\nabla_{\mathbf{dC}_i}\psi_{i+1}^{(M)} + \sin[M\Psi_{i+1}]\nabla_{\mathbf{dC}_i}\phi_{i+1}^{(M)}\right] \\
&\times \left(\cos[M\Psi_{i+2}]\phi_{i+2}^{(M)} - \sin[M\Psi_{i+2}]\psi_{i+2}^{(M)}\right) \\
&\times \left(\cos[M\Psi_{i+3}]\phi_{i+3}^{(M)} - \sin[M\Psi_{i+3}]\psi_{i+3}^{(M)}\right)
\end{aligned}
\tag{S10}
$$

$$
\begin{aligned}
\nabla_{\mathbf{dC}_{i+1}} B_{7,i,M} = &\left[\cos[M\Psi_{i+1}]\nabla_{\mathbf{dC}_{i+1}}\psi_{i+1}^{(M)} + \sin[M\Psi_{i+1}]\nabla_{\mathbf{dC}_{i+1}}\phi_{i+1}^{(M)}\right] \\
&\times \left(\cos[M\Psi_{i+2}]\phi_{i+2}^{(M)} - \sin[M\Psi_{i+2}]\psi_{i+2}^{(M)}\right) \\
&\times \left(\cos[M\Psi_{i+3}]\phi_{i+3}^{(M)} - \sin[M\Psi_{i+3}]\psi_{i+3}^{(M)}\right) \\
&+ \left[\cos[M\Psi_{i+1}]\psi_{i+1}^{(M)} + \sin[M\Psi_{i+1}]\phi_{i+1}^{(M)}\right] \\
&\times \left(\cos[M\Psi_{i+2}]\nabla_{\mathbf{dC}_{i+1}}\phi_{i+2}^{(M)} - \sin[M\Psi_{i+2}]\nabla_{\mathbf{dC}_{i+1}}\psi_{i+2}^{(M)}\right) \\
&\times \left(\cos[M\Psi_{i+3}]\phi_{i+3}^{(M)} - \sin[M\Psi_{i+3}]\psi_{i+3}^{(M)}\right)
\end{aligned}
\tag{S11}
$$

$$
\begin{aligned}
\nabla_{\mathbf{dC}_{i+2}} B_{7,i,M} = &\left[\cos[M\Psi_{i+1}]\nabla_{\mathbf{dC}_{i+2}}\psi_{i+1}^{(M)} + \sin[M\Psi_{i+1}]\nabla_{\mathbf{dC}_{i+2}}\phi_{i+1}^{(M)}\right] \\
&\times \left(\cos[M\Psi_{i+2}]\phi_{i+2}^{(M)} - \sin[M\Psi_{i+2}]\psi_{i+2}^{(M)}\right) \\
&\times \left(\cos[M\Psi_{i+3}]\phi_{i+3}^{(M)} - \sin[M\Psi_{i+3}]\psi_{i+3}^{(M)}\right) \\
&+ \left[\cos[M\Psi_{i+1}]\psi_{i+1}^{(M)} + \sin[M\Psi_{i+1}]\phi_{i+1}^{(M)}\right] \\
&\times \left(\cos[M\Psi_{i+2}]\nabla_{\mathbf{dC}_{i+2}}\phi_{i+2}^{(M)} - \sin[M\Psi_{i+2}]\nabla_{\mathbf{dC}_{i+2}}\psi_{i+2}^{(M)}\right) \\
&\times \left(\cos[M\Psi_{i+3}]\phi_{i+3}^{(M)} - \sin[M\Psi_{i+3}]\psi_{i+3}^{(M)}\right) \\
&+ \left[\cos[M\Psi_{i+1}]\psi_{i+1}^{(M)} + \sin[M\Psi_{i+1}]\phi_{i+1}^{(M)}\right] \\
&\times \left(\cos[M\Psi_{i+2}]\phi_{i+2}^{(M)} - \sin[M\Psi_{i+2}]\psi_{i+2}^{(M)}\right) \\
&\times \left(\cos[M\Psi_{i+3}]\nabla_{\mathbf{dC}_{i+2}}\phi_{i+3}^{(M)} - \sin[M\Psi_{i+3}]\nabla_{\mathbf{dC}_{i+2}}\psi_{i+3}^{(M)}\right)
\end{aligned}
\tag{S12}
$$

$$
\begin{aligned}
\nabla_{\mathbf{dC}_{i+3}} B_{7,i,M} = & \left[\cos[M\Psi_{i+1}]\psi_{i+1}^{(M)} + \sin[M\Psi_{i+1}]\phi_{i+1}^{(M)}\right] \\
& \times \left(\cos[M\Psi_{i+2}]\nabla_{\mathbf{dC}_{i+3}}\phi_{i+2}^{(M)} - \sin[M\Psi_{i+2}]\nabla_{\mathbf{dC}_{i+3}}\psi_{i+2}^{(M)}\right) \\
& \times \left(\cos[M\Psi_{i+3}]\phi_{i+3}^{(M)} - \sin[M\Psi_{i+3}]\psi_{i+3}^{(M)}\right) \\
& + \left[\cos[M\Psi_{i+1}]\psi_{i+1}^{(M)} + \sin[M\Psi_{i+1}]\phi_{i+1}^{(M)}\right] \\
& \times \left(\cos[M\Psi_{i+2}]\phi_{i+2}^{(M)} - \sin[M\Psi_{i+2}]\psi_{i+2}^{(M)}\right) \\
& \times \left(\cos[M\Psi_{i+3}]\nabla_{\mathbf{dC}_{i+3}}\phi_{i+3}^{(M)} - \sin[M\Psi_{i+3}]\nabla_{\mathbf{dC}_{i+3}}\psi_{i+3}^{(M)}\right)
\end{aligned} \tag{S13}
$$

$$
\begin{aligned}
\nabla_{\mathbf{dC}_{i+4}} B_{7,i,M} = & \left[\cos[M\Psi_{i+1}]\psi_{i+1}^{(M)} + \sin[M\Psi_{i+1}]\phi_{i+1}^{(M)}\right] \\
& \times \left(\cos[M\Psi_{i+2}]\phi_{i+2}^{(M)} - \sin[M\Psi_{i+2}]\psi_{i+2}^{(M)}\right) \\
& \times \left(\cos[M\Psi_{i+3}]\nabla_{\mathbf{dC}_{i+4}}\phi_{i+3}^{(M)} - \sin[M\Psi_{i+3}]\nabla_{\mathbf{dC}_{i+3}}\psi_{i+4}^{(M)}\right)
\end{aligned} \tag{S14}
$$

$$
\nabla_{\mathbf{dC}_{i+5}} B_{7,i,M} = 0 \tag{S15}
$$

For the segments with length $m > 7$, a recursive formula given by Equation (S16) is applied.

$$
\begin{aligned}
\nabla_{\mathbf{dC}_k} B_{m,i,M} = & \nabla_{\mathbf{dC}_k} B_{m-1,i,M} \times \left(\cos[M\Psi_{i+m-4}]\phi_{i+m-4}^{(M)} - \sin[M\Psi_{i+m-4}]\psi_{i+m-4}^{(M)}\right) \\
& + B_{m-1,i,M} \times \left(\cos[M\Psi_{i+m-4}]\nabla_{\mathbf{dC}_k}\phi_{i+m-4}^{(M)} - \sin[M\Psi_{i+m-4}]\nabla_{\mathbf{dC}_k}\psi_{i+m-4}^{(M)}\right)
\end{aligned} \tag{S16}
$$

With the above formulas, the gradient of $U^f_{mtor;i;m;M}$ is expressed by Equation (S17).

$$\nabla_{\mathbf{dC}_k} U^f_{mtor;i;m;M} = -w_M \times \nabla_{\mathbf{dC}_k} B_{m,i,M} \left[\cos[M\Psi_{i+m-3}]\psi^{(M)}_{i+m-3} + \sin[M\Psi_{i+m-3}]\phi^{(M)}_{i+m-3}\right]$$
$$- w_M \times B_{m,i,M} \left[\cos[M\Psi_{i+m-3}]\nabla_{\mathbf{dC}_k}\psi^{(M)}_{i+m-3} + \sin[M\Psi_{i+m-3}]\nabla_{\mathbf{dC}_k}\phi^{(M)}_{i+m-3}\right] \tag{S17}$$

The gradients $\nabla_{\mathbf{dC}_z}\psi_k^{(M)}$ and $\nabla_{\mathbf{dC}_z}\phi_k^{(M)}$ (for $z = k-1$, $z = k$ and $z = k+1$) are expressed by Equations (25) – (42) of ref.[S1]

References

(S1) A. K. Sieradzan, J. Sans-Duñó, E. A. Lubecka, C. Czaplewski, A. G. Lipska, H. Leszczynski, K. M. Ocetkiewicz, J. Proficz, P. Czarnul, H. Krawczyk, A. Liwo, *J. Comput. Chem.* **2023**, *44*, 602.

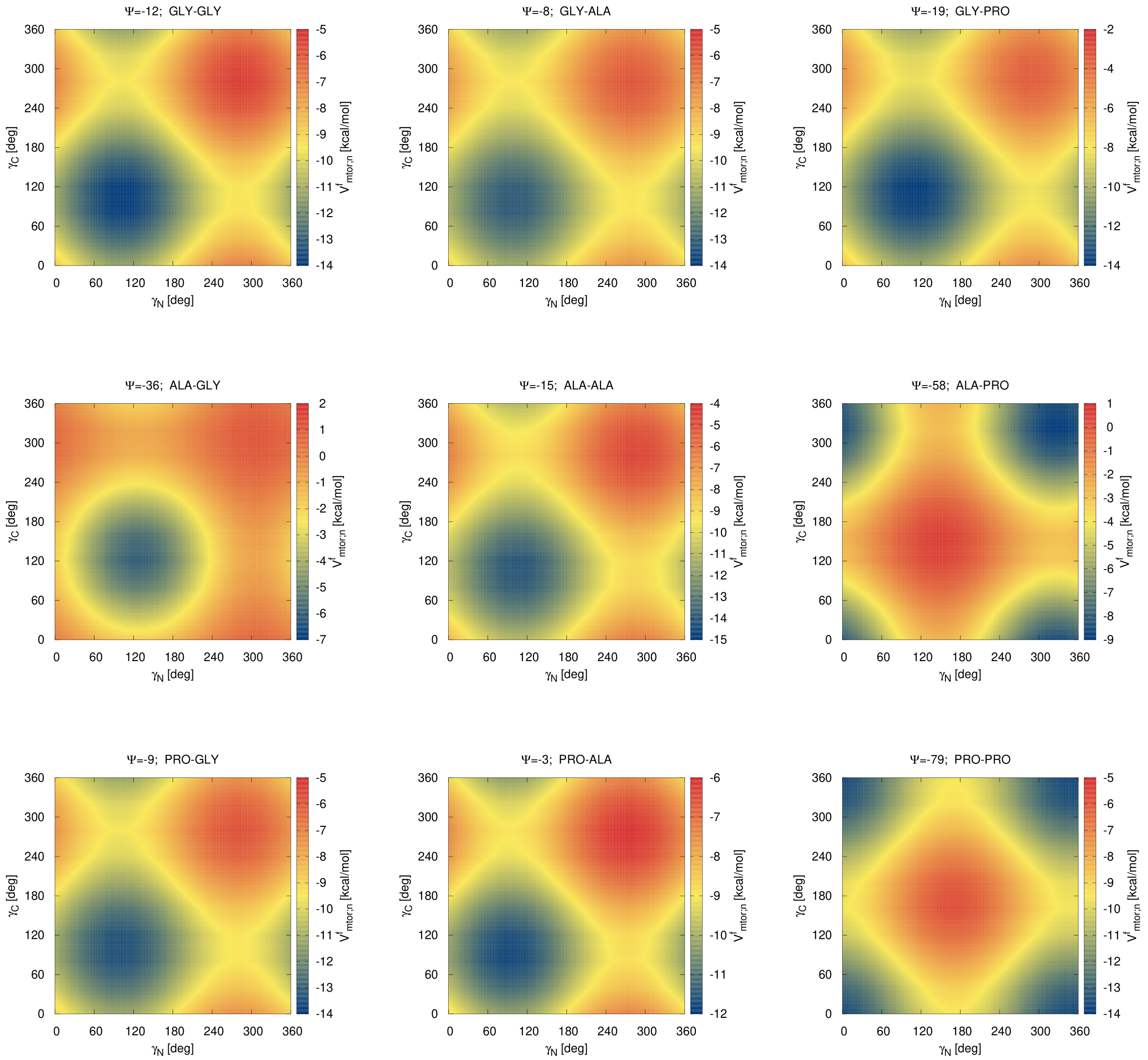

Figure S1: Heat maps of $V^{f}_{mtor;n}$ [Equation (6) of the main text] in the terminal virtual-bond dihedral angles γ_N and γ_C of a 20-residue FH segment obtained with parameter set A

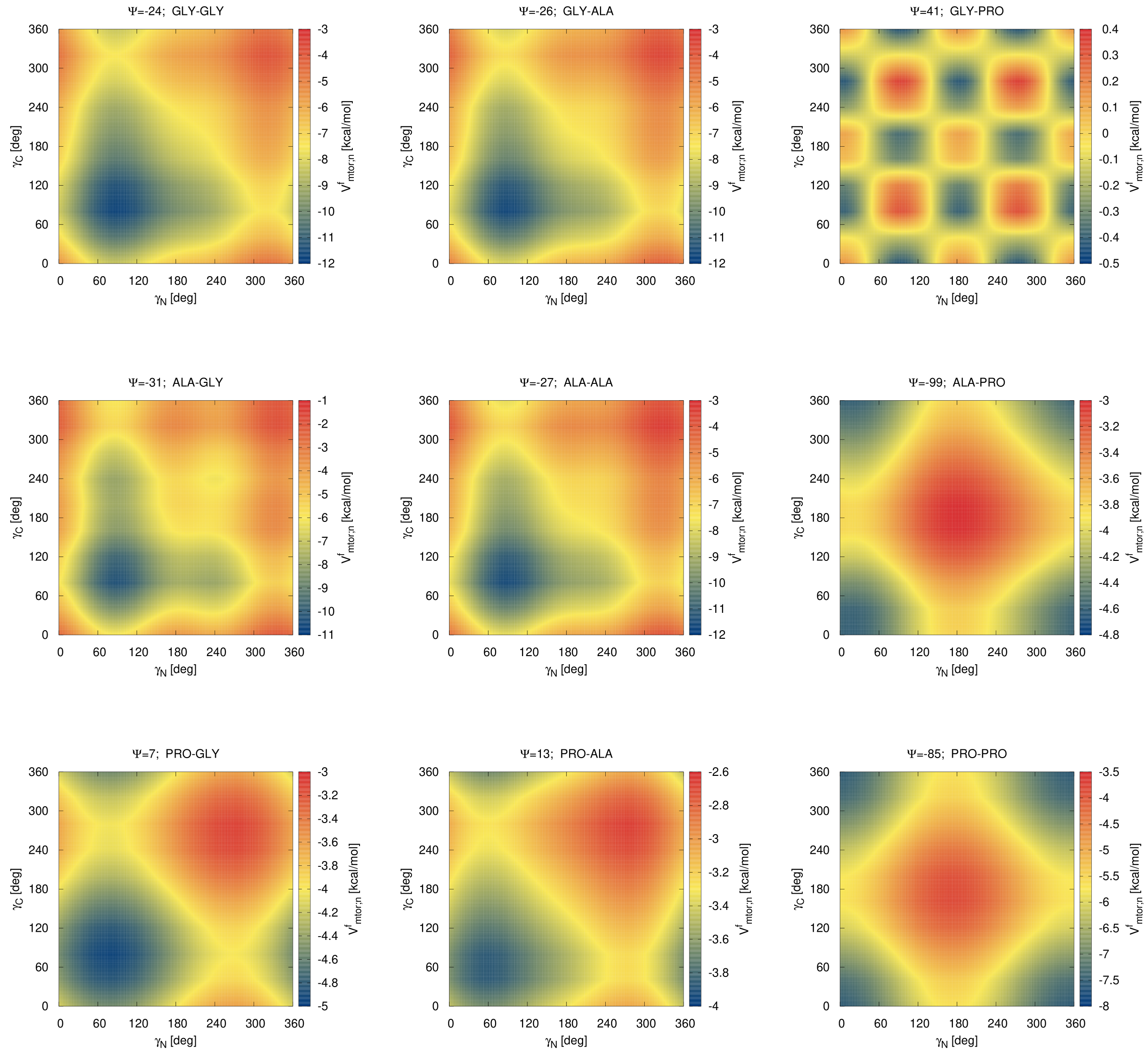

Figure S2: Heat maps of $V^{f}_{mtor;n}$ [Equation (6) of the main text] in the terminal virtual-bond dihedral angles γ_N and γ_C of a 20-residue FH segment obtained with parameter set B

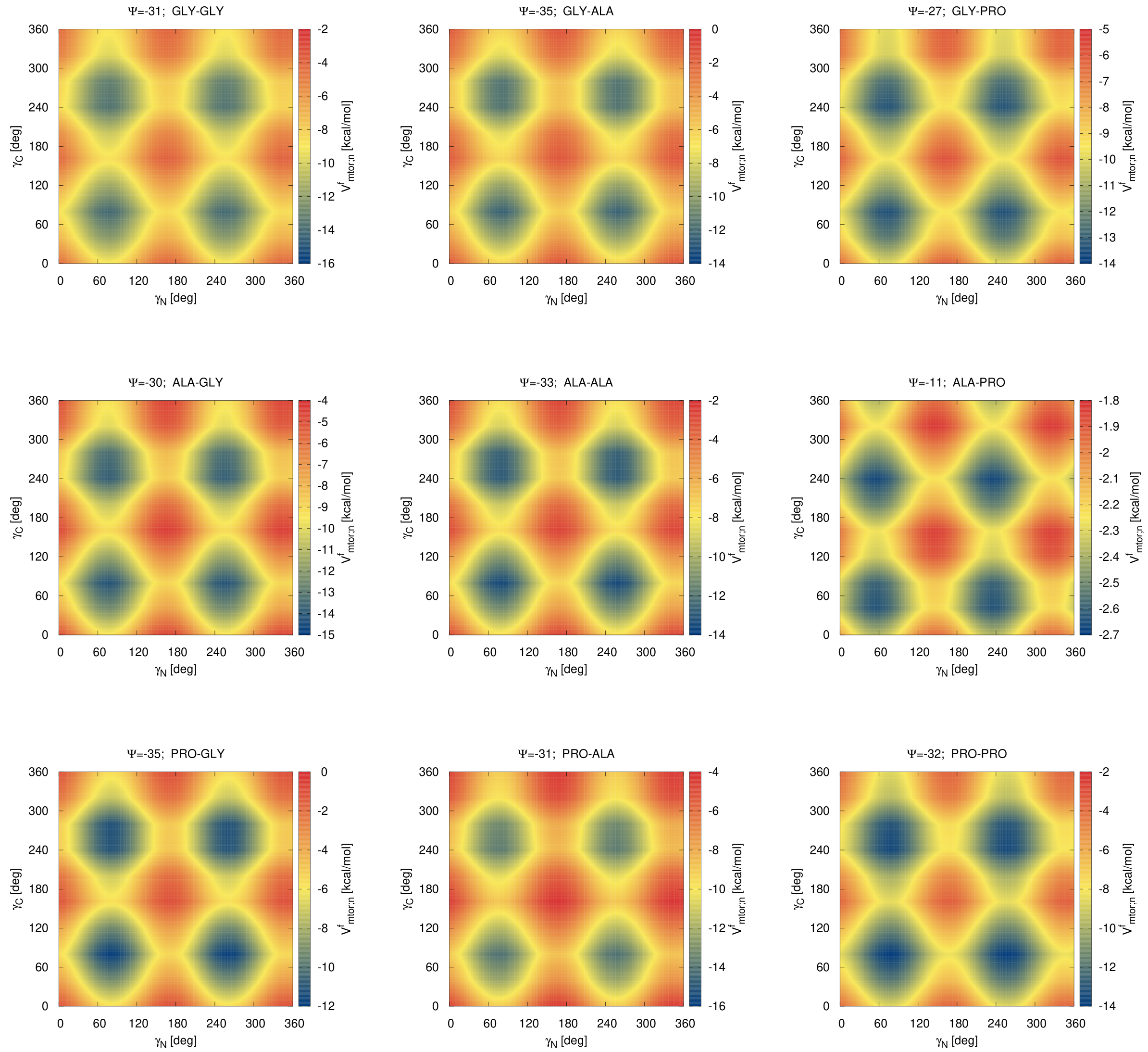

Figure S3: Heat maps of $V^f_{mtor;n}$ [Equation (6) of the main text] in the terminal virtual-bond dihedral angles γ_N and γ_C of a 20-residue FH segment obtained with parameter set C

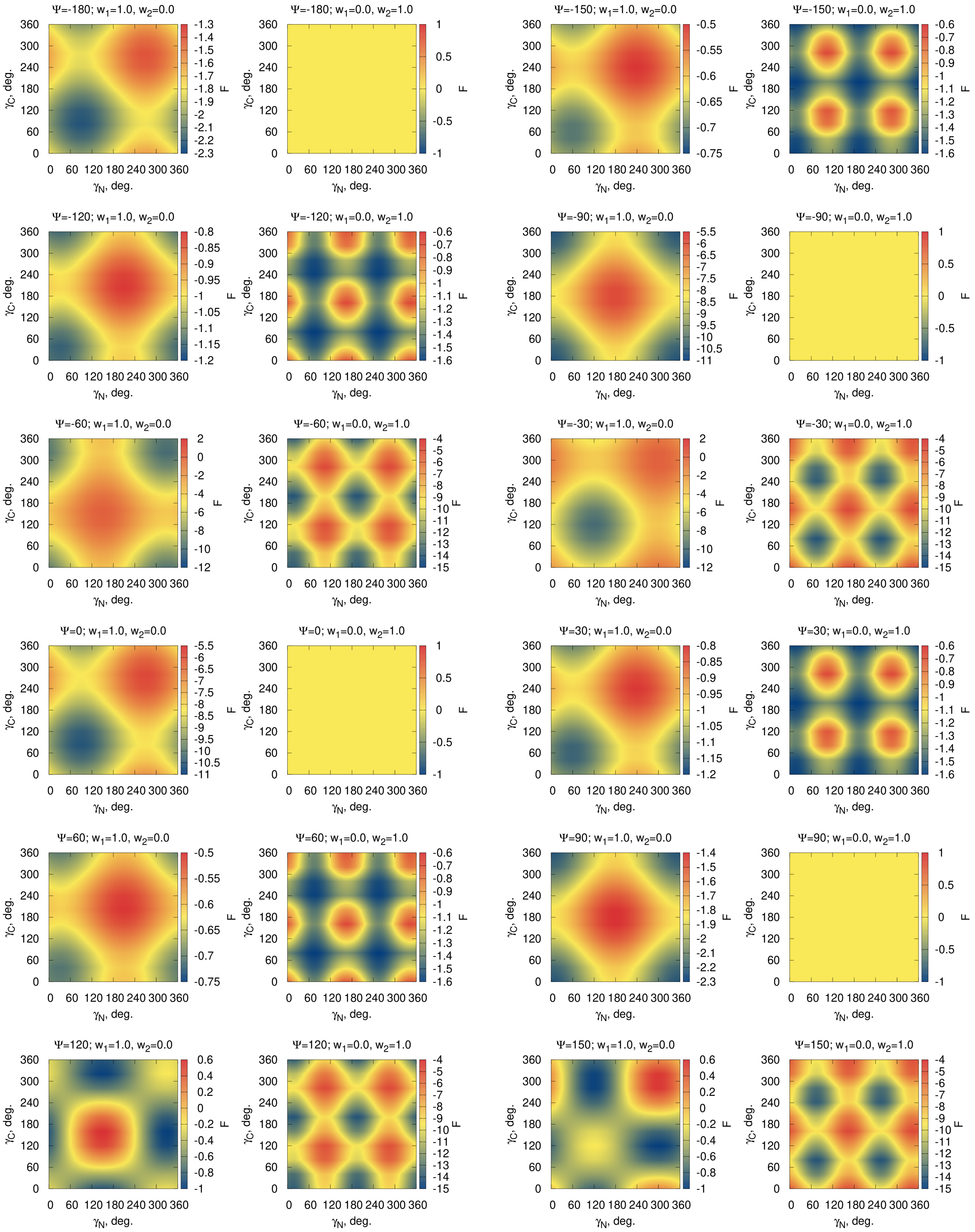

Figure S4: Heat maps of $V^f_{mtor;20}$ [Equation (6) of the main text] of a 20-residue FH segment in the terminal γ_N and γ_C angles and various phase angles Ψ.

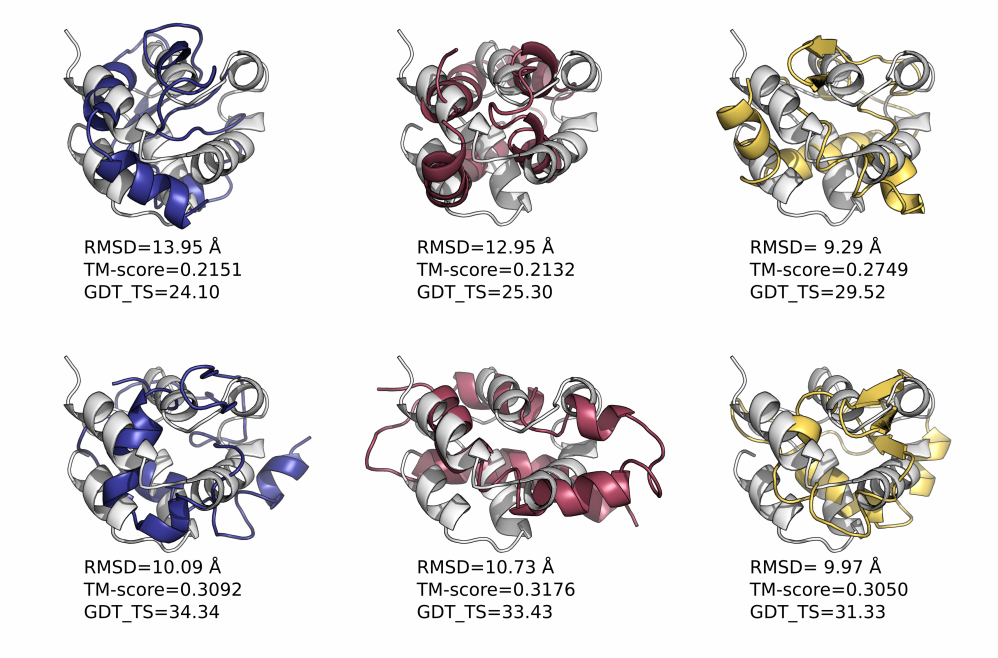

Figure S5: Protein **1A1W**. The first models (top) and the best models (with the highest GDT_TS; bottom) obtained with plain UNRES (blue), multitorsional potential with parameter set A, $w_{mtor} = 0.2$ (red), multitorsional potential with parameter set B, $w_{mtor} = 0.15$ (yellow) superposed on the experimental structure (white). The GDT_TS, TM-score, and RMSD values are shown.

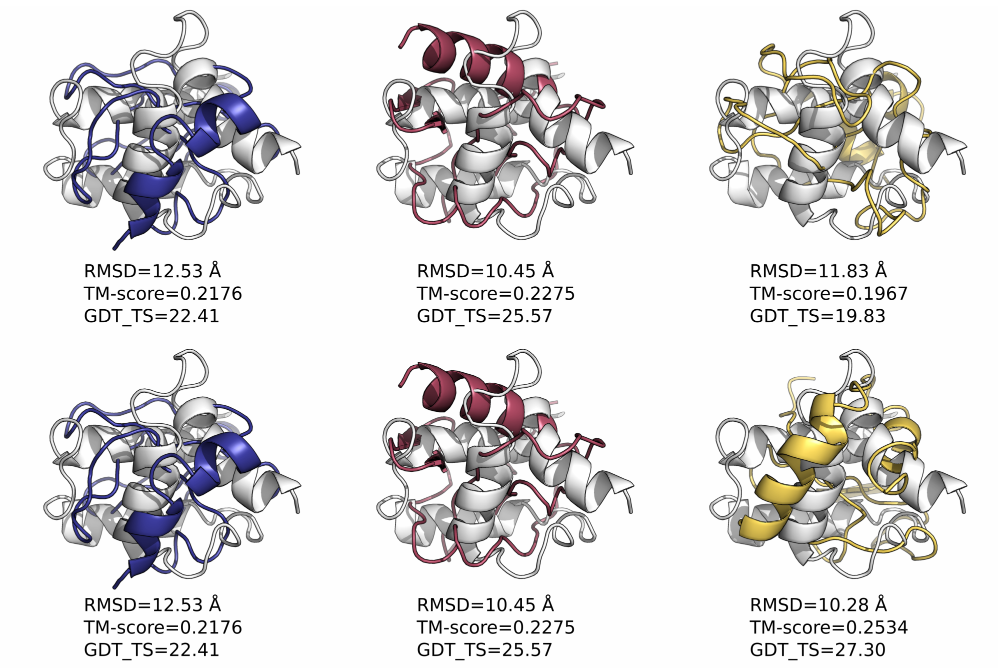

Figure S6: Protein **1A6S**. For the description see the caption of Fig. S5

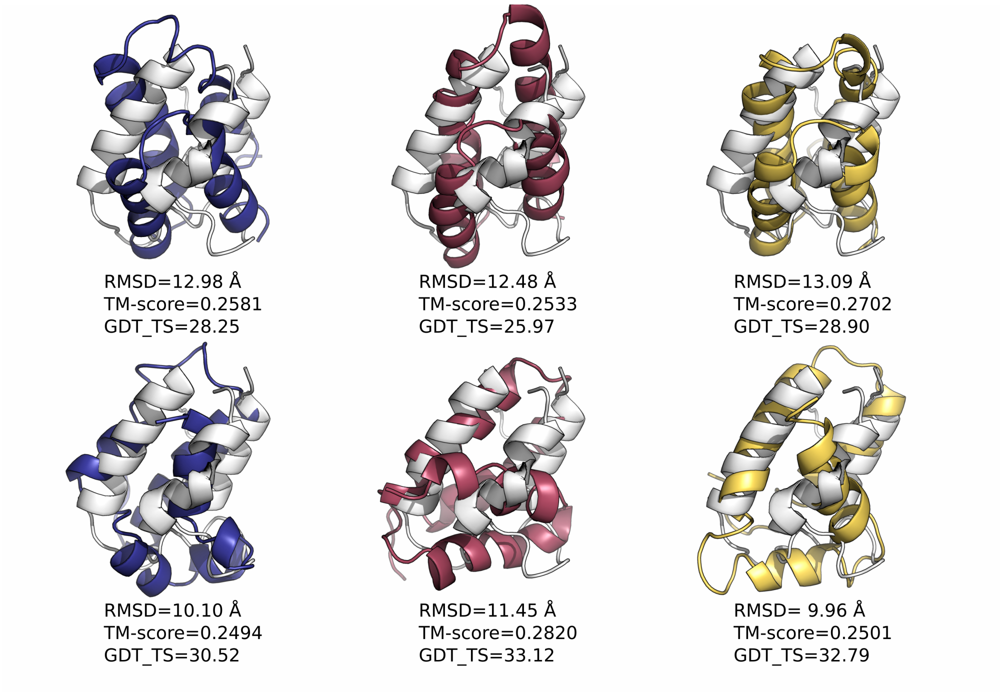

Figure S7: Protein **1ACP**. For the description see the caption of Fig. S5

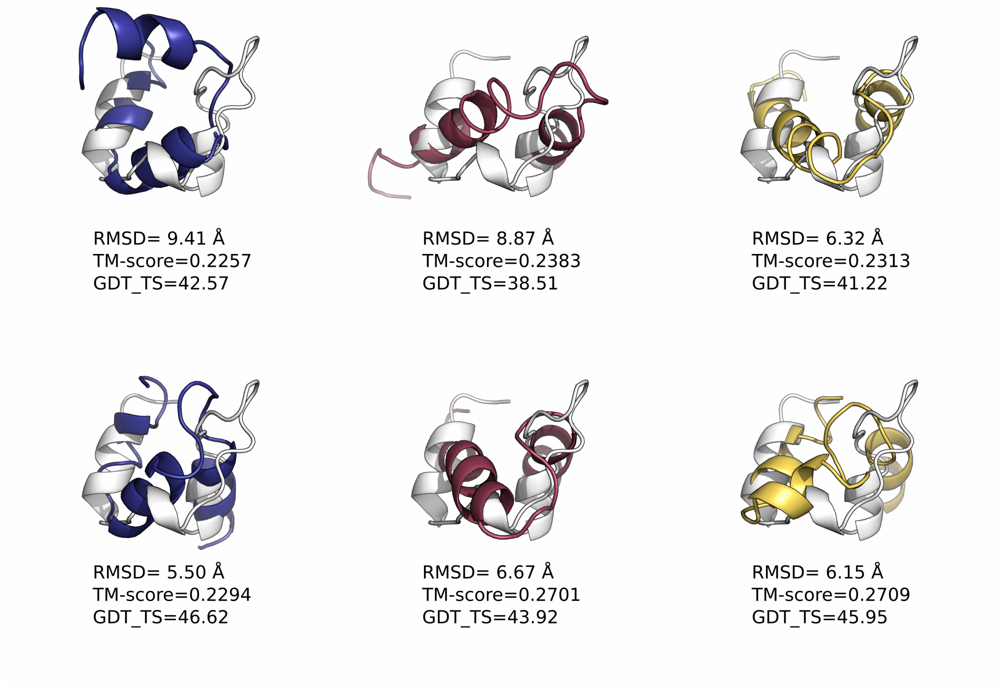

Figure S8: Protein **1BBL**. For the description see the caption of Fig. S5

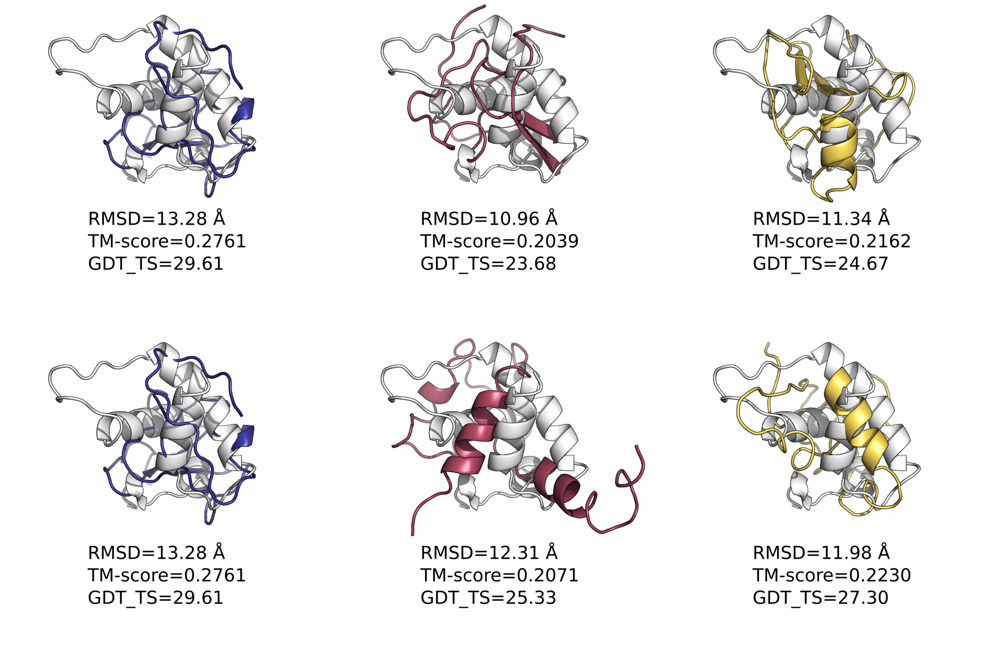

Figure S9: Protein **1BG8**. For the description see the caption of Fig. S5

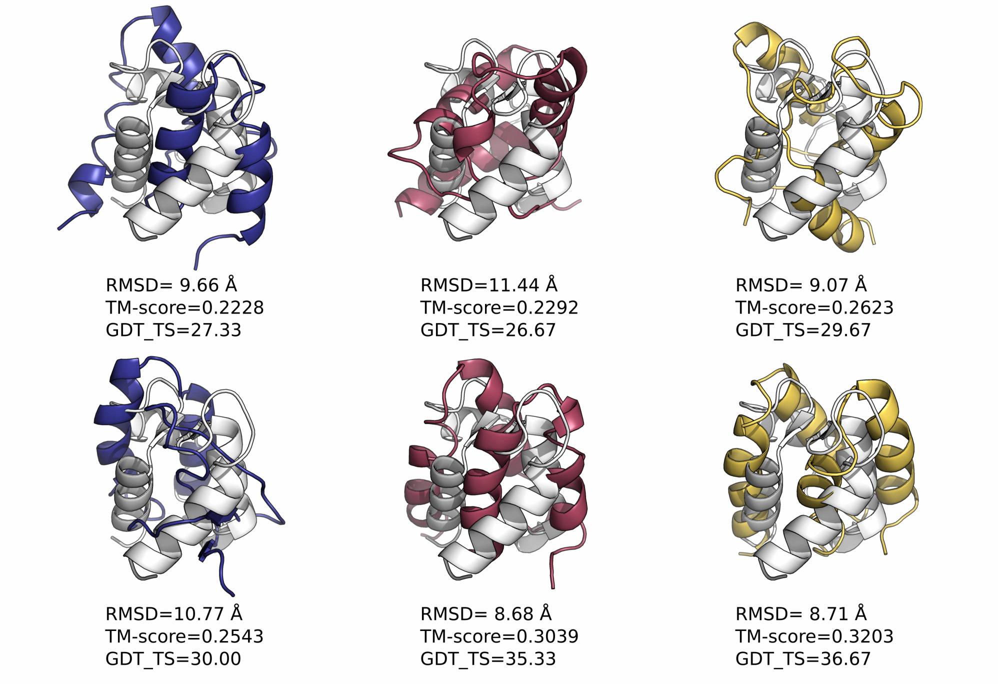

Figure S10: Protein **1CLB**. For the description see the caption of Fig. S5

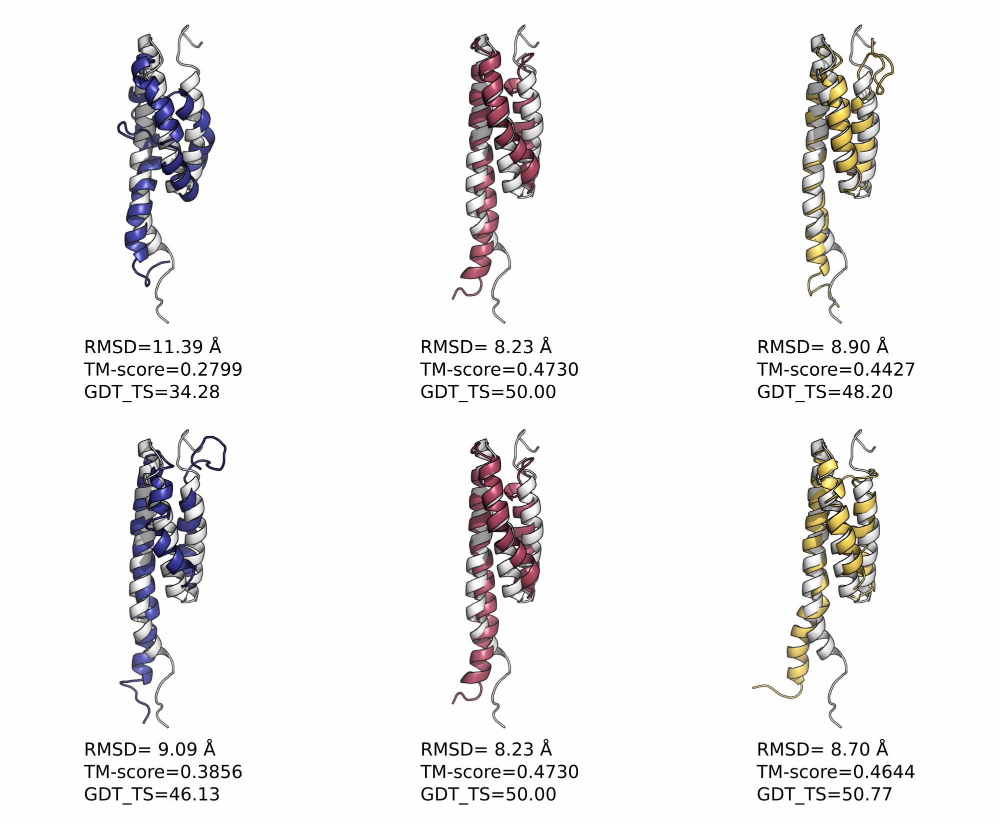

Figure S11: Protein **2CRB**. For the description see the caption of Fig. S5

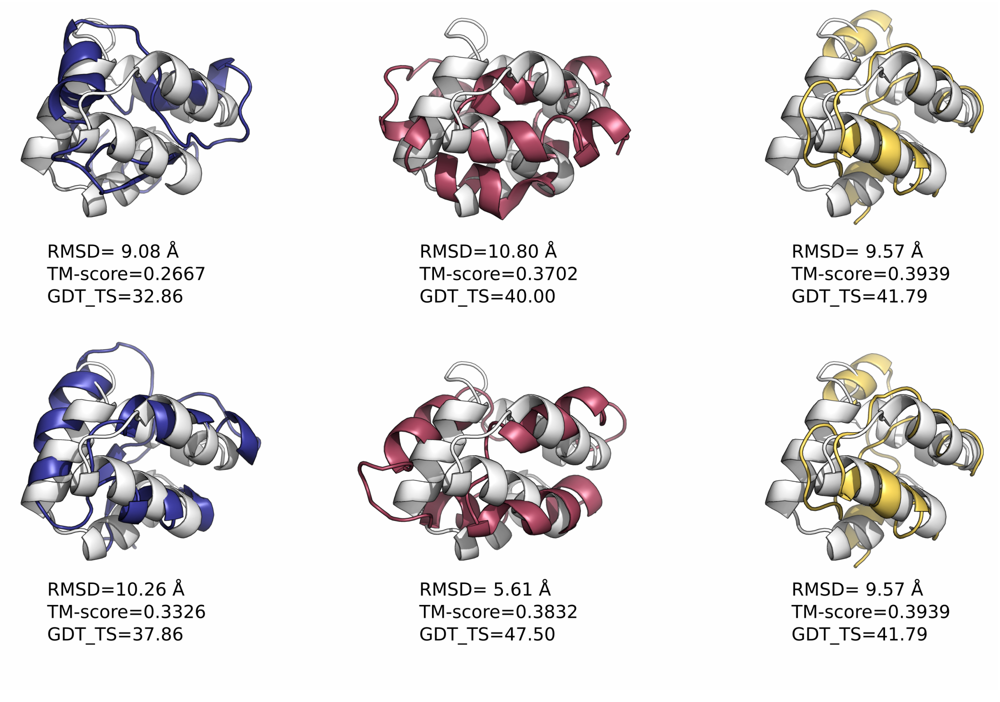

Figure S12: Protein **1E68**. For the description see the caption of Fig. S5

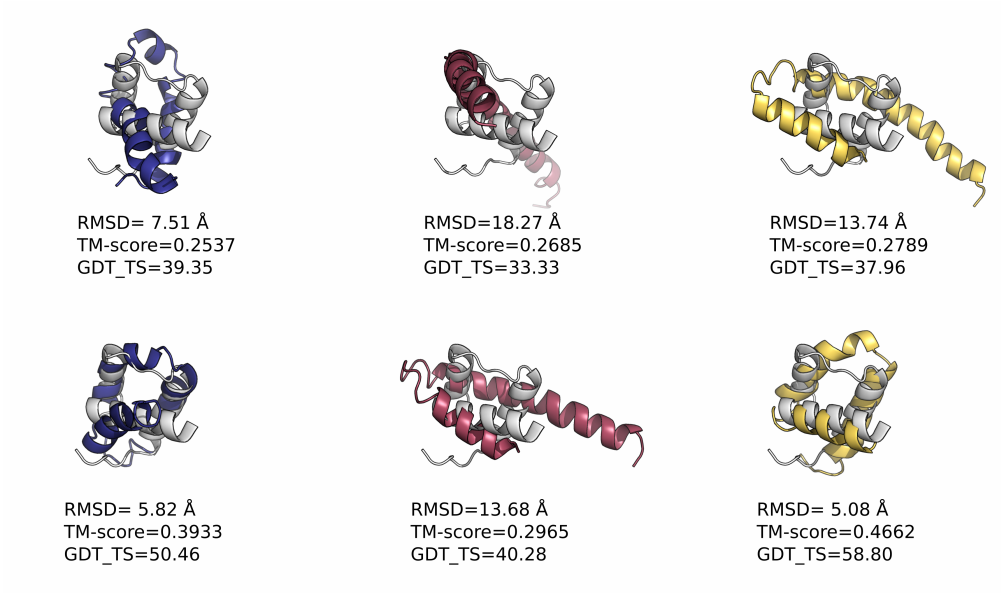

Figure S13: Protein **1ENH**. For the description see the caption of Fig. S5

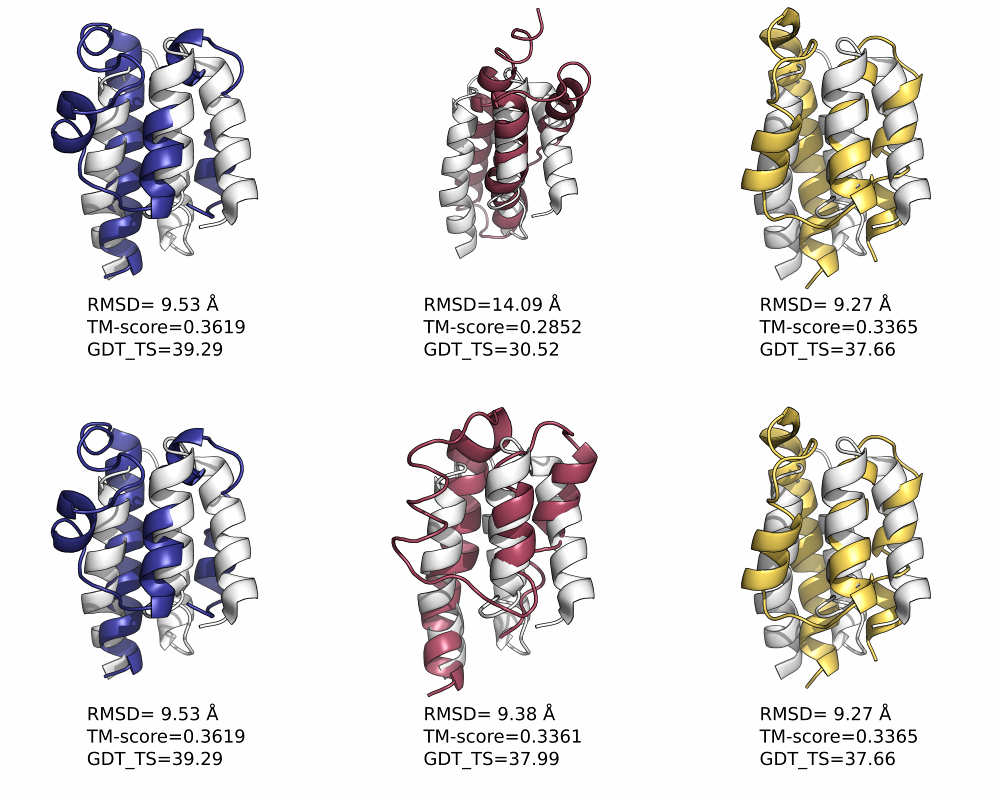

Figure S14: Protein **1EO0**. For the description see the caption of Fig. S5

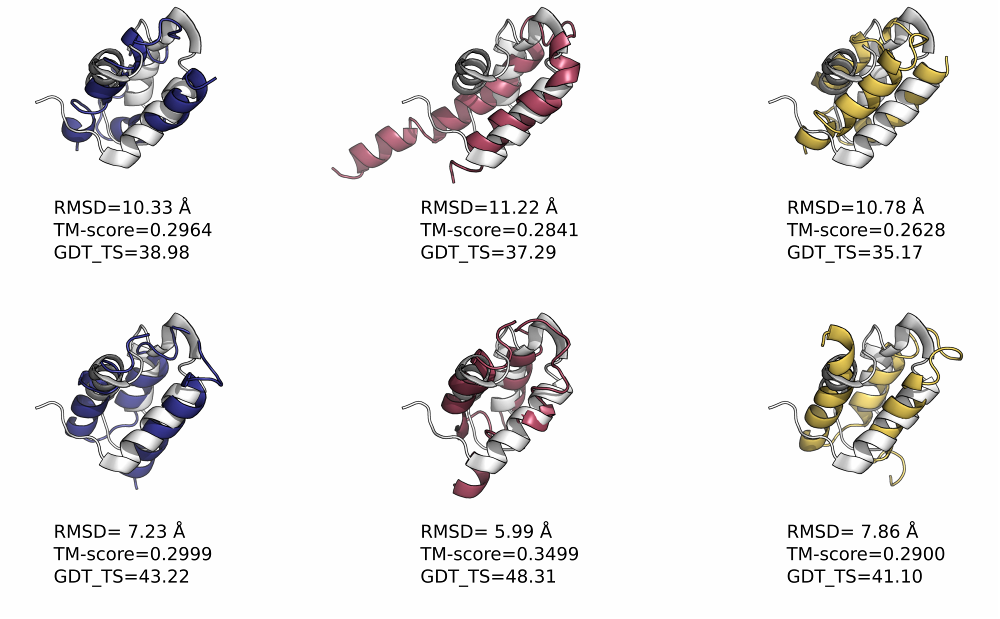

Figure S15: Protein **1FEX**. For the description see the caption of Fig. S5

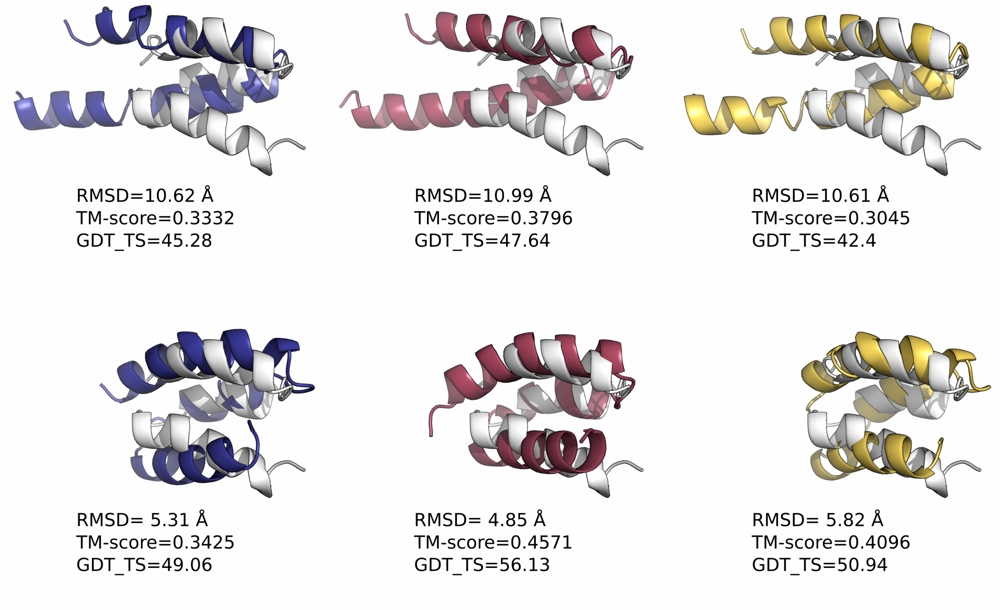

Figure S16: Protein **1GAB**. For the description see the caption of Fig. S5

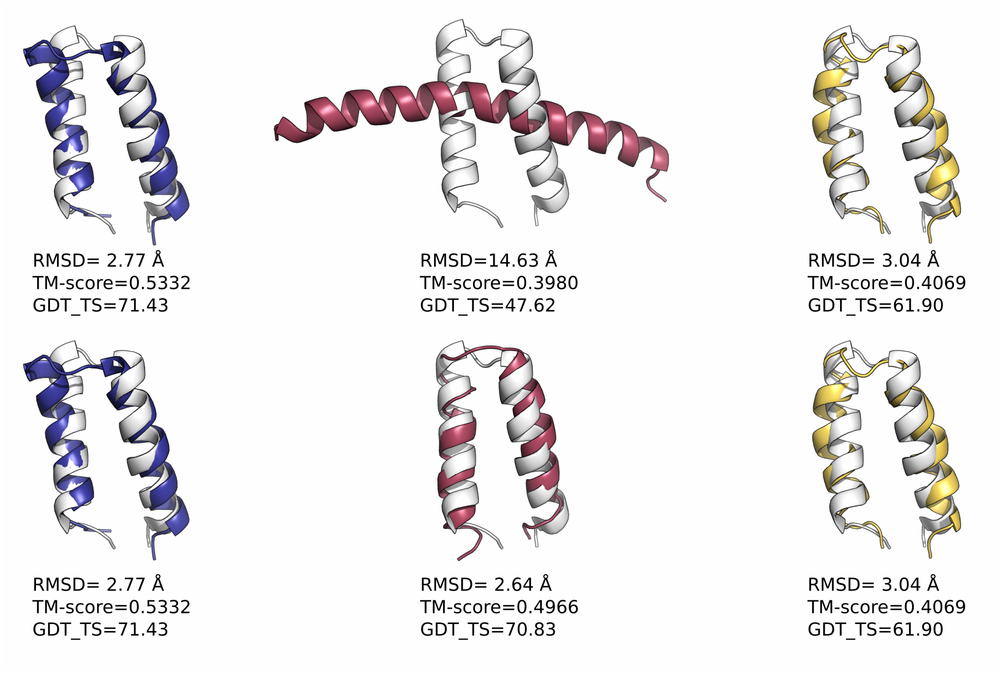

Figure S17: Protein **2HEP**. For the description see the caption of Fig. S5

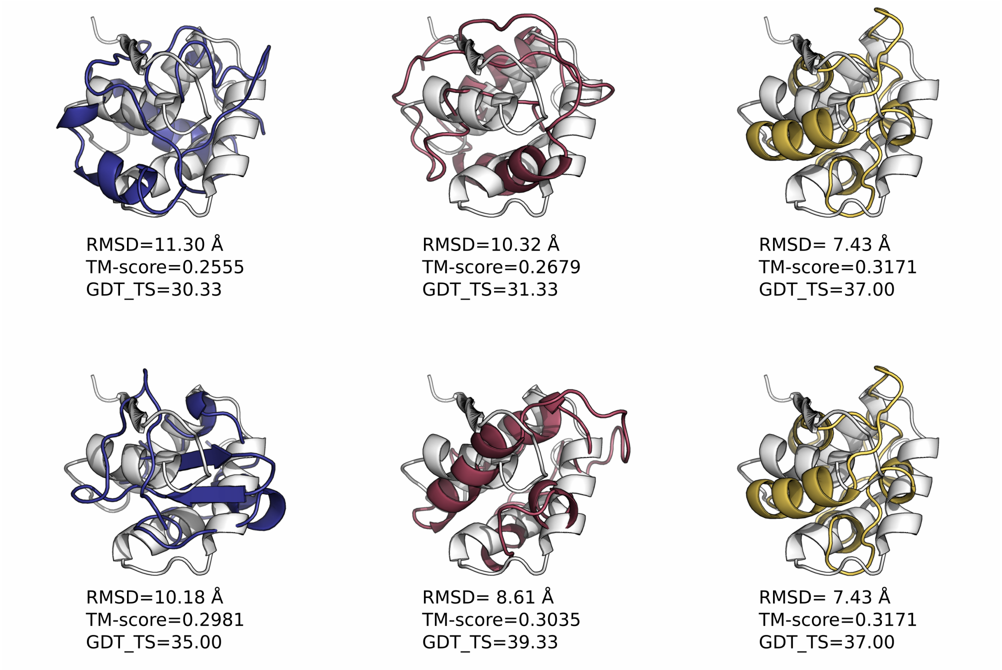

Figure S18: Protein **1HYP**. For the description see the caption of Fig. S5

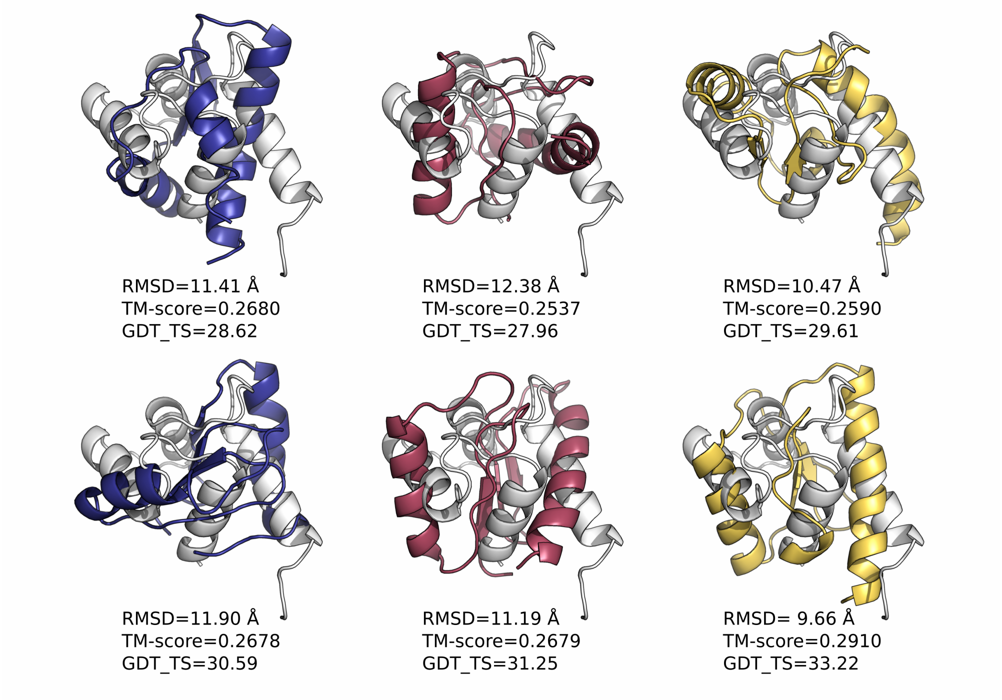

Figure S19: Protein **1J7O**. For the description see the caption of Fig. S5

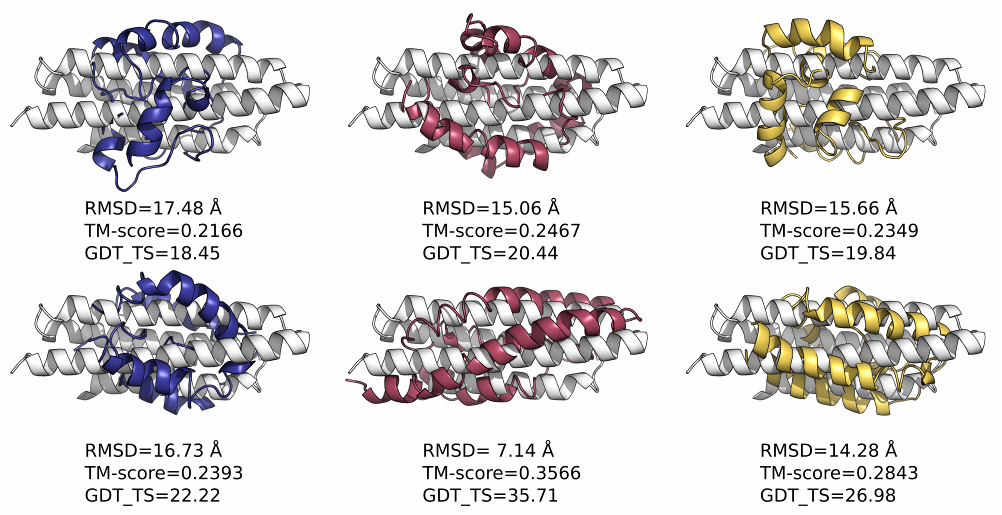

Figure S20: Protein **1K40**. For the description see the caption of Fig. S5

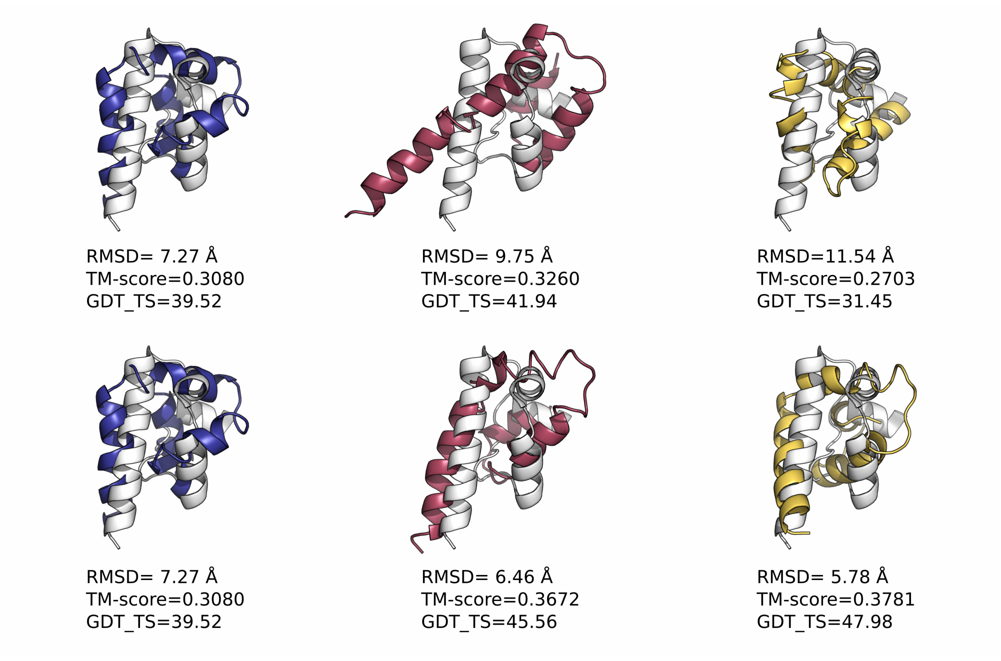

Figure S21: Protein **1KOY**. For the description see the caption of Fig. S5

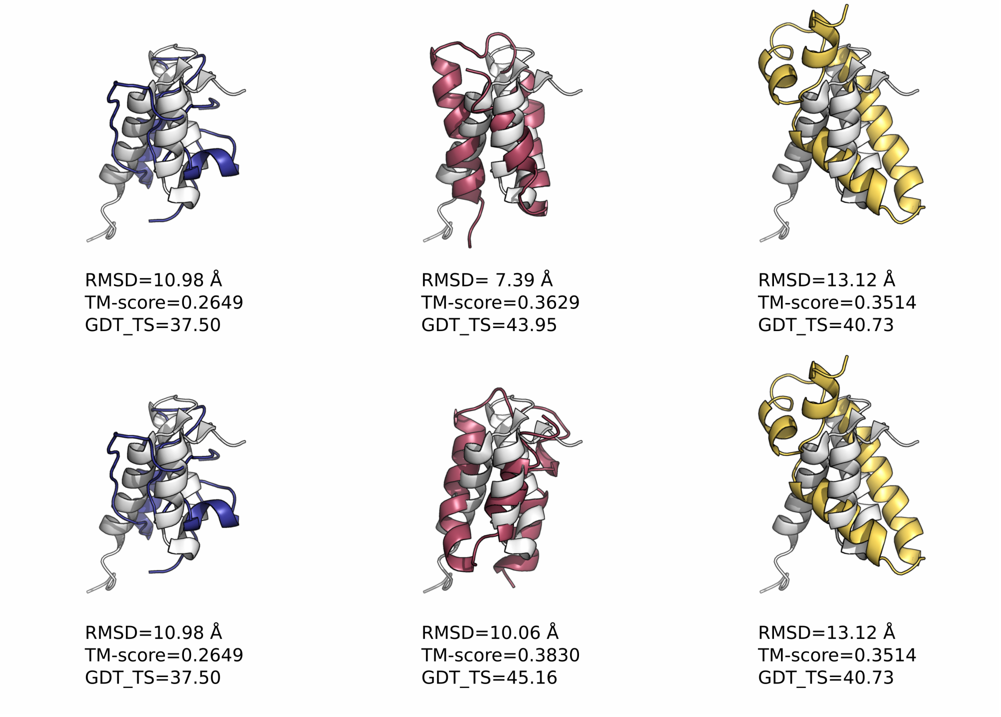

Figure S22: Protein **2L09**. For the description see the caption of Fig. S5

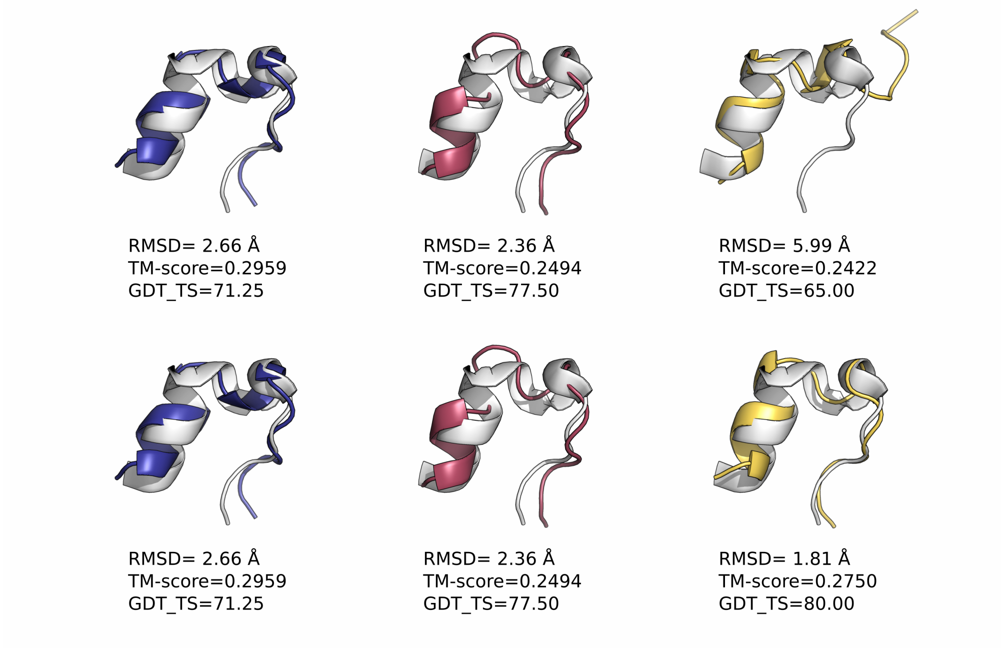

Figure S23: Protein **1L2Y**. For the description see the caption of Fig. S5

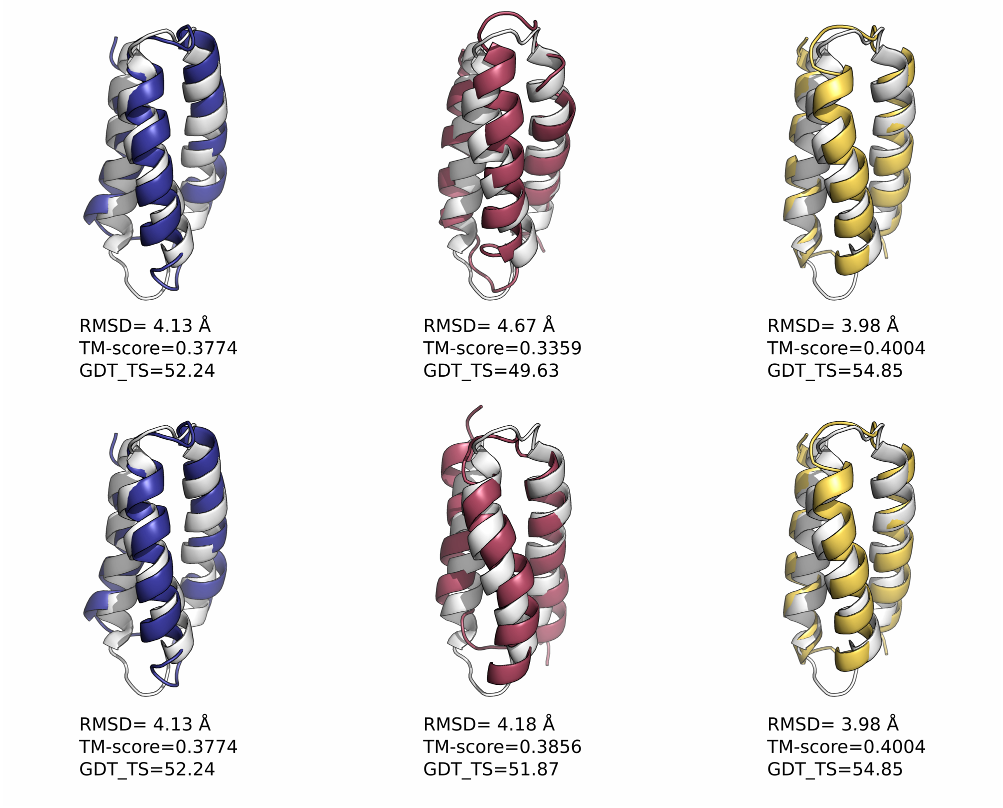

Figure S24: Protein **1LQ7**. For the description see the caption of Fig. S5

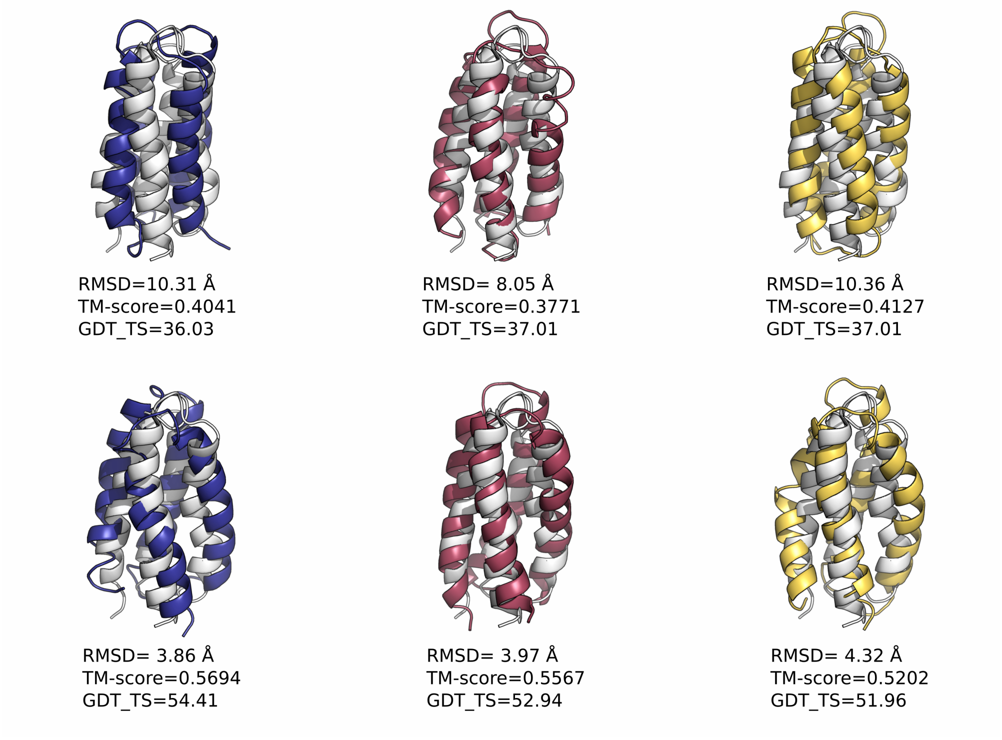

Figure S25: Protein **1P68**. For the description see the caption of Fig. S5

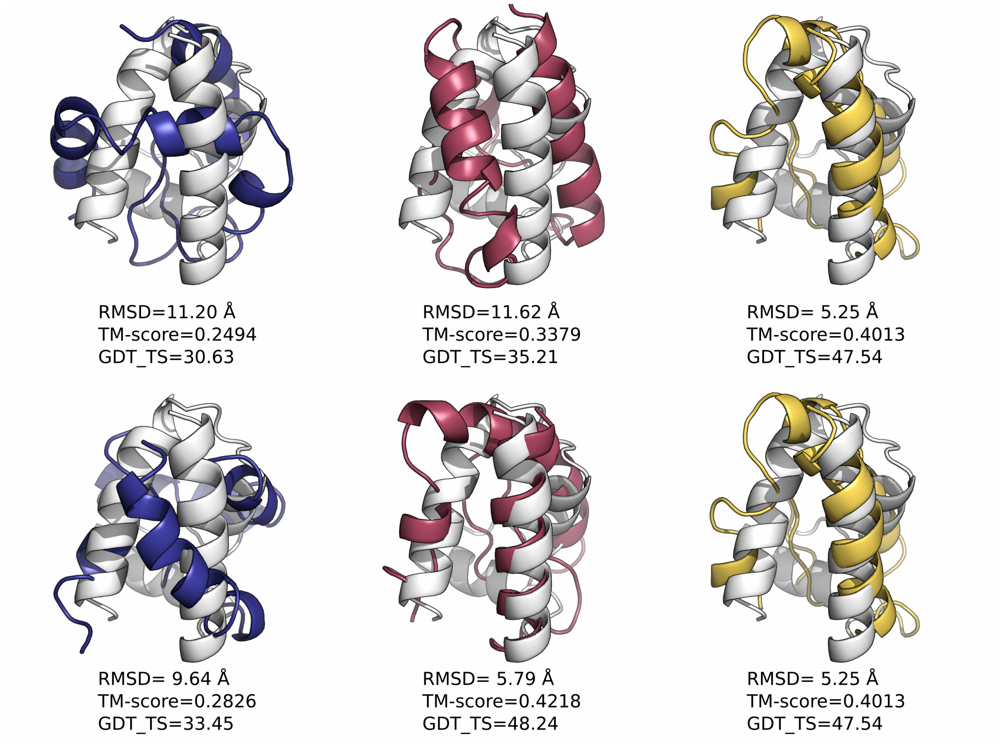

Figure S26: Protein **1POU**. For the description see the caption of Fig. S5

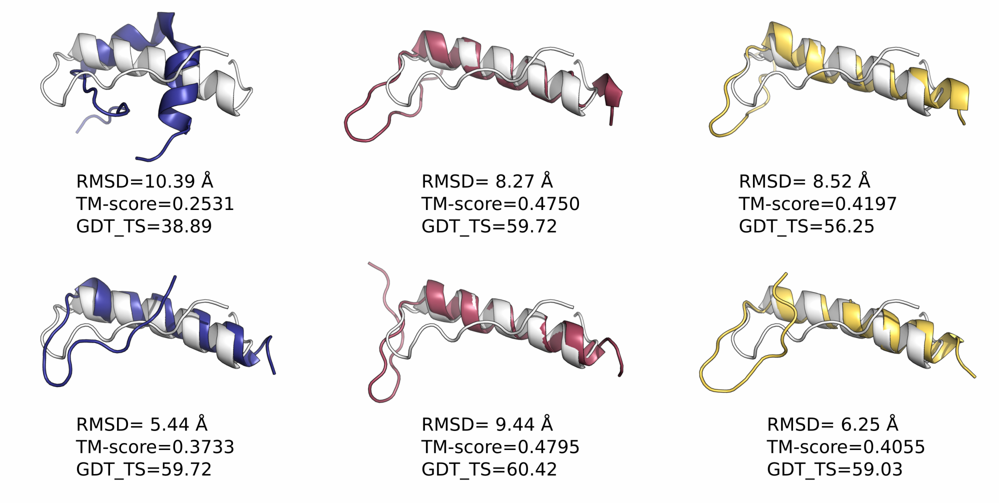

Figure S27: Protein **1PPT**. For the description see the caption of Fig. S5

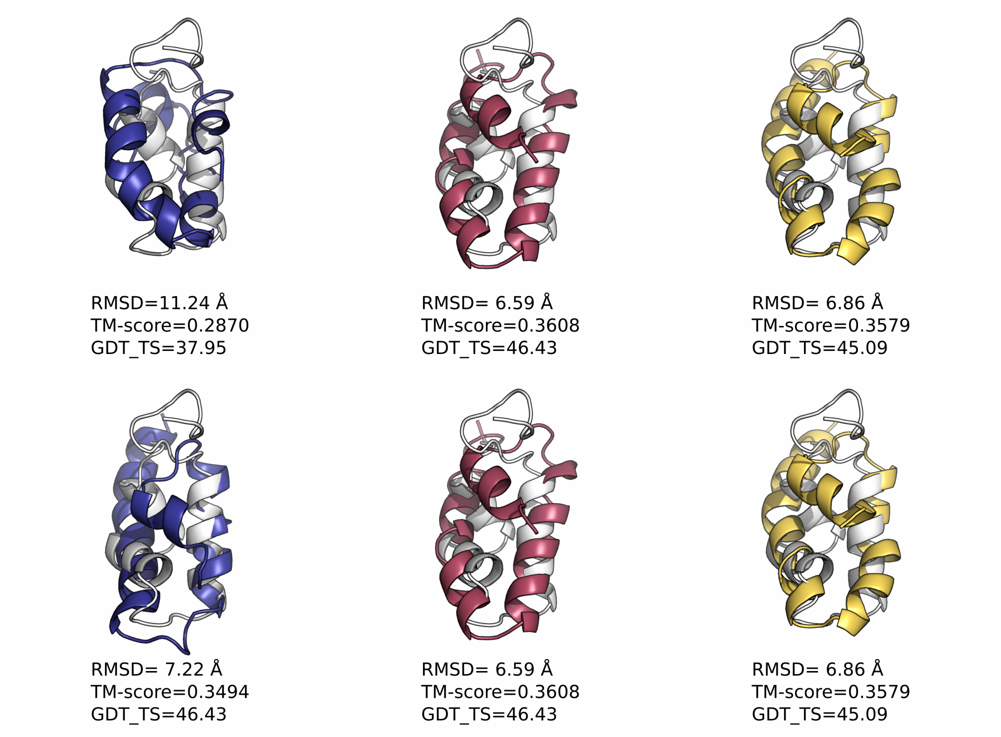

Figure S28: Protein **1PRU**. For the description see the caption of Fig. S5

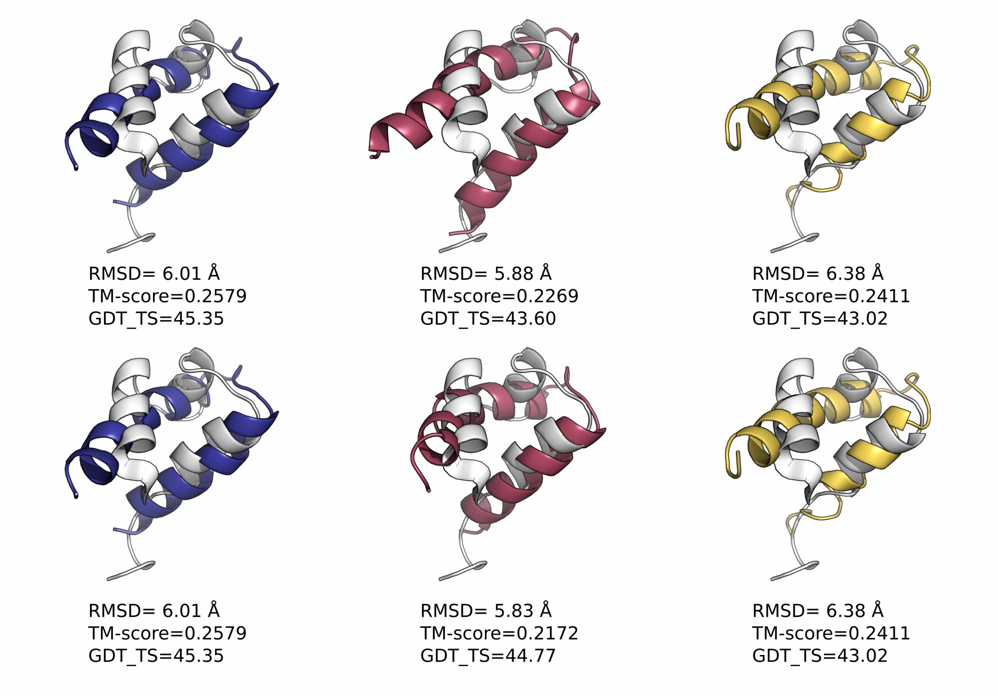

Figure S29: Protein **1RES**. For the description see the caption of Fig. S5

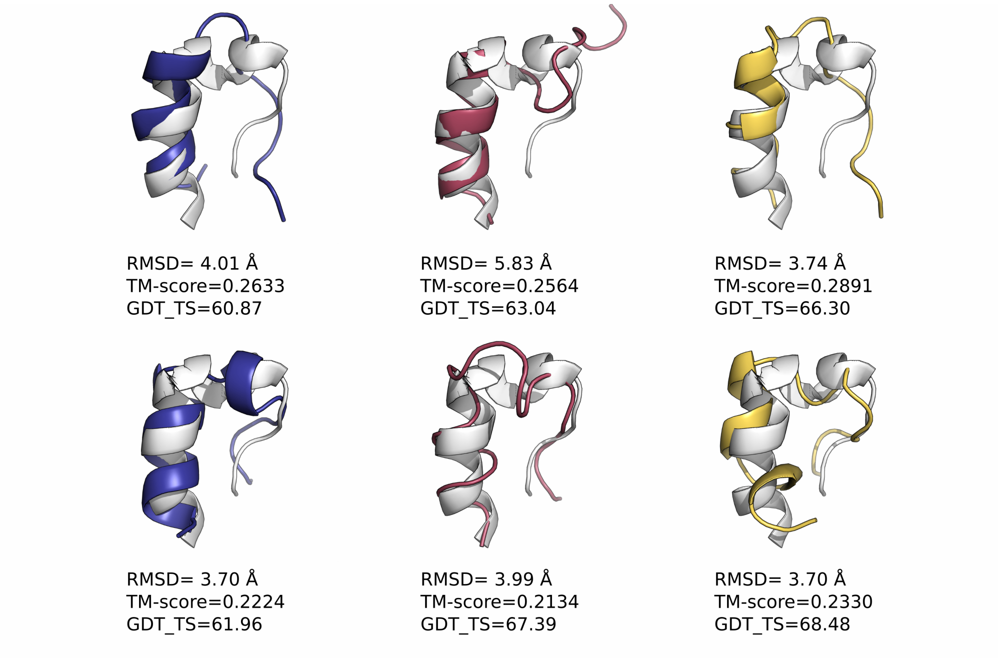

Figure S30: Protein **1RIJ**. For the description see the caption of Fig. S5

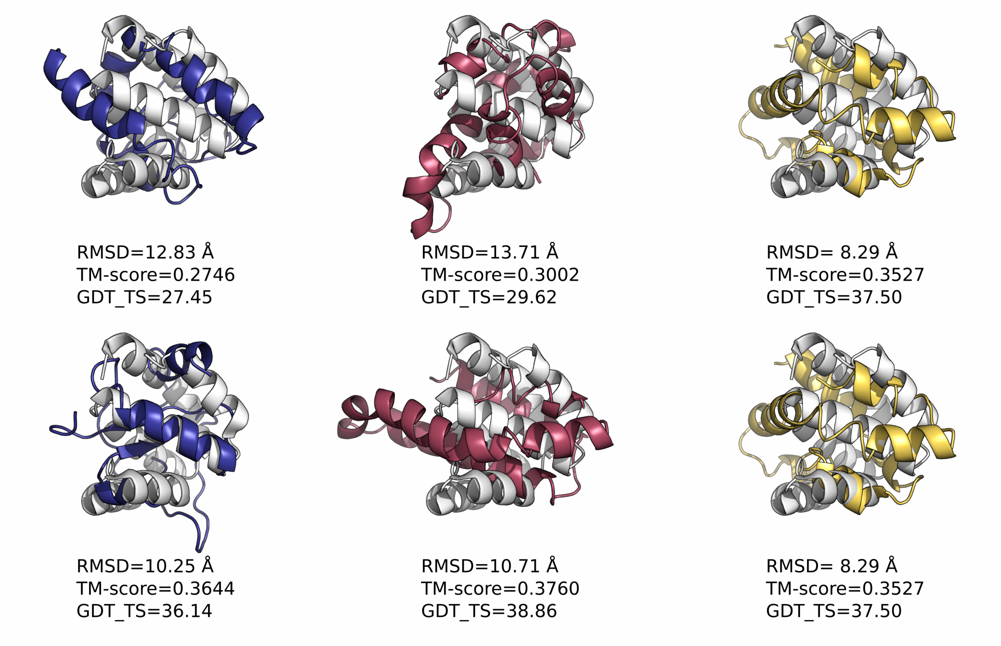

Figure S31: Protein **2YGS**. For the description see the caption of Fig. S5

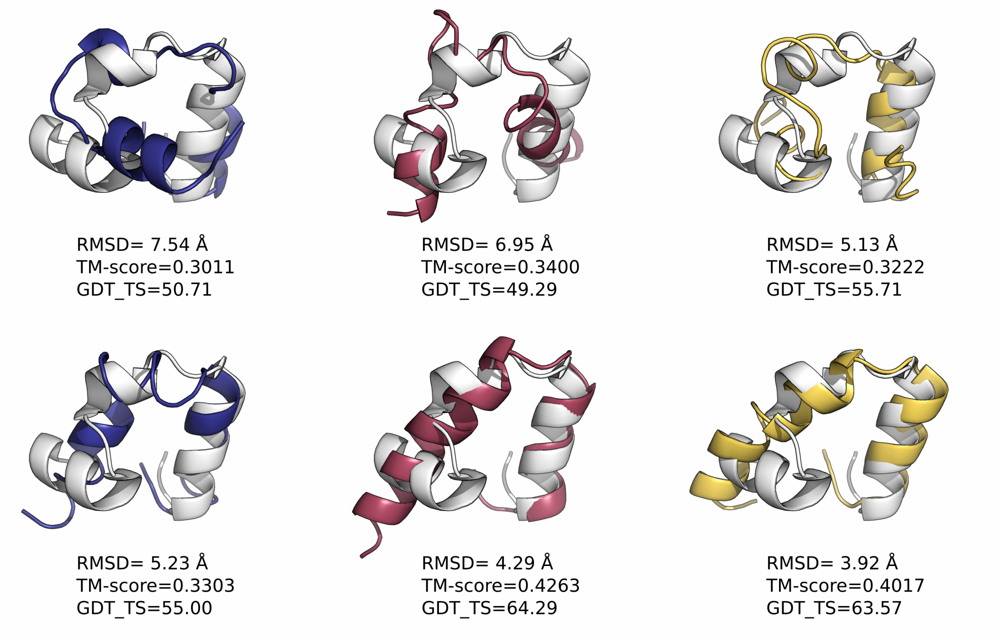

Figure S32: Protein **1YRF**. For the description see the caption of Fig. S5

Table S1: Characteristics of the first (top-probability) and the measure-best models of the structures of the benchmark proteins obtained using state-of-the-art UNRES, without multitorsional potential: $w_{mtor} = 0.0$.

protein	number of residues	First model				Measure-best model								
		P [%][a]	RMSD [Å]	TM-score	GDT-TS	ncl[b]	P [%][a]	RMSD [Å]	ncl[b]	P [%][a]	TM-score	ncl[b]	P [%][a]	GDT-TS
1A1W	83	27	13.9	0.215	24.1	3	22	10.1	3	22	0.309	3	22	34.3
1A6S	87	60	12.5	0.218	22.4	5	5	12.2	1	60	0.218	1	60	22.4
1ACP	77	30	13.0	0.258	28.2	2	26	10.1	1	30	0.258	2	26	30.5
1BBL	37	33	9.4	0.226	42.6	5	9	5.5	2	26	0.259	5	9	46.6
1BG8	76	57	13.3	0.276	29.6	4	6	12.8	1	57	0.276	1	57	29.6
1CLB	75	38	9.7	0.223	27.3	1	38	9.7	5	4	0.254	5	4	30.0
2CRB	97	31	11.4	0.280	34.3	2	25	9.1	2	25	0.386	2	25	46.1
1E68	70	31	9.1	0.267	32.9	3	18	8.1	2	30	0.333	2	30	37.9
1ENH	54	27	7.5	0.254	39.4	3	18	5.8	3	18	0.393	3	18	50.5
1EO0	77	29	9.5	0.362	39.3	3	17	8.0	1	29	0.362	1	29	39.3
1FEX	59	34	10.3	0.296	39.0	4	13	7.2	4	13	0.300	4	13	43.2
1GAB	53	28	10.6	0.333	45.3	2	26	5.3	2	26	0.343	2	26	49.1
2HEP	42	31	2.8	0.533	71.4	1	31	2.8	1	31	0.533	1	31	71.4
1HYP	75	58	11.3	0.256	30.3	2	17	9.9	2	17	0.314	5	1	35.0
1J7O	76	56	11.4	0.268	28.6	3	10	10.9	5	5	0.293	2	17	30.6
1K40	126	53	17.5	0.217	18.4	3	15	14.9	4	9	0.267	2	16	22.2
1KOY	62	24	7.3	0.308	39.5	2	21	7.2	1	24	0.308	1	24	39.5
2L09	62	40	11.0	0.265	37.5	2	28	10.9	4	13	0.270	1	40	37.5
1L2Y	20	32	2.7	0.296	71.2	1	32	2.7	1	32	0.296	1	32	71.2
1LQ7	67	40	4.1	0.377	52.2	1	40	4.1	4	10	0.405	1	40	52.2
1P68	102	40	10.3	0.404	36.0	4	12	3.9	4	12	0.569	4	12	54.4
1POU	71	27	11.2	0.249	30.6	3	18	8.2	4	16	0.283	2	21	33.5
1PPT	36	28	10.4	0.253	38.9	3	19	5.4	2	27	0.413	3	19	59.7
1PRU	56	31	11.2	0.287	38.0	3	17	7.2	3	17	0.349	3	17	46.4
1RES	43	25	6.0	0.258	45.4	1	25	6.0	3	22	0.271	1	25	45.4
1RIJ	23	32	4.0	0.263	60.9	3	22	3.7	1	32	0.263	3	22	62.0
2YGS	92	32	12.8	0.275	27.4	4	11	10.2	4	11	0.364	4	11	36.1
1YRF	35	32	7.5	0.301	50.7	2	25	5.2	2	25	0.330	2	25	55.0

[a]The contribution of the family of the respective model to the conformational ensemble at T=300 K.
[b]The rank of the respective family (according to population).

Table S2: Characteristics of the first (top-probability) and the measure-best models of the structures of the benchmark proteins obtained using UNRES with multitorsional potential: parameter set A, $w_{mtor} = 0.10$.

protein	number of residues	First model				Measure-best model								
		P [%][a]	RMSD [Å]	TM-score	GDT-TS	ncl[b]	P [%][a]	RMSD [Å]	ncl[b]	P [%][a]	TM-score	ncl[b]	P [%][a]	GDT-TS
1A1W	83	34	13.9	0.229	25.6	5	9	10.7	2	30	0.295	2	30	31.9
1A6S	87	49	11.0	0.211	22.1	1	49	11.0	3	16	0.226	3	16	23.9
1ACP	77	32	13.1	0.266	28.9	2	28	10.0	1	32	0.266	2	28	33.1
1BBL	37	26	9.0	0.266	40.5	5	15	6.2	1	26	0.266	5	15	45.3
1BG8	76	46	11.5	0.244	26.6	5	11	9.0	1	46	0.244	5	11	27.3
1CLB	75	29	11.9	0.248	32.3	3	21	9.4	3	21	0.272	3	21	33.3
2CRB	97	37	9.0	0.423	43.0	3	20	8.4	1	37	0.423	1	37	43.0
1E68	70	30	11.6	0.340	34.6	5	4	9.0	1	30	0.340	5	4	38.6
1ENH	54	35	15.8	0.269	35.2	4	14	6.1	4	14	0.415	4	14	54.6
1EO0	77	27	9.0	0.377	41.6	3	16	8.9	1	27	0.377	1	27	41.6
1FEX	59	28	10.4	0.287	37.7	4	18	7.1	4	18	0.304	4	18	44.5
1GAB	53	48	11.1	0.347	44.8	2	23	5.6	2	23	0.376	2	23	50.0
2HEP	42	27	3.3	0.434	66.7	1	27	3.3	1	27	0.434	1	27	66.7
1HYP	75	47	11.8	0.280	32.0	2	20	8.1	2	20	0.284	2	20	35.0
1J7O	76	33	11.1	0.272	31.6	2	25	8.2	2	25	0.331	3	23	32.9
1K40	126	30	12.4	0.297	27.8	1	30	12.4	2	30	0.313	1	30	27.8
1KOY	62	25	6.0	0.316	42.7	1	25	6.0	2	23	0.344	2	23	43.5
2L09	62	34	11.3	0.222	28.6	2	31	8.0	2	31	0.329	2	31	40.3
1L2Y	20	32	5.8	0.229	57.5	4	12	1.8	3	19	0.286	4	12	82.5
1LQ7	67	33	4.8	0.321	48.9	1	33	4.8	2	30	0.339	1	33	48.9
1P68	102	34	10.2	0.378	35.0	3	18	4.1	3	18	0.548	3	18	54.2
1POU	71	29	11.6	0.259	29.6	2	26	6.5	2	26	0.340	2	26	40.5
1PPT	36	31	9.4	0.275	43.1	3	13	5.5	2	30	0.499	4	12	62.5
1PRU	56	46	6.7	0.341	44.2	4	12	6.5	1	46	0.341	1	46	44.2
1RES	43	37	6.0	0.234	41.3	1	37	6.0	5	4	0.276	2	24	41.9
1RIJ	23	30	4.0	0.286	63.0	1	30	4.0	1	30	0.286	1	30	63.0
2YGS	92	37	12.2	0.247	26.6	2	23	11.1	2	23	0.284	2	23	28.0
1YRF	35	39	7.4	0.483	63.6	3	21	3.9	1	39	0.483	3	21	63.6

[a]The contribution of the family of the respective model to the conformational ensemble at T=300 K.

[b]The rank of the respective family (according to population).

Table S3: Characteristics of the first (top-probability) and the measure-best models of the structures of the benchmark proteins obtained using UNRES with multitorsional potential: parameter set A, $w_{mtor} = 0.15$.

protein	number of residues	First model				Measure-best model								
		P [%][a]	RMSD [Å]	TM-score	GDT-TS	ncl[b]	P [%][a]	RMSD [Å]	ncl[b]	P [%][a]	TM-score	ncl[b]	P [%][a]	GDT-TS
1A1W	83	39	13.6	0.224	26.5	5	7	11.3	3	17	0.296	5	7	31.3
1A6S	87	31	11.1	0.282	28.4	3	17	11.1	1	31	0.282	1	31	28.4
1ACP	77	30	13.0	0.252	29.6	3	22	10.5	3	22	0.260	3	22	32.5
1BBL	37	31	6.3	0.213	43.2	1	31	6.3	3	19	0.245	1	31	43.2
1BG8	76	38	11.2	0.232	24.0	3	12	11.1	1	38	0.232	4	11	26.6
1CLB	75	26	9.0	0.268	30.0	3	20	8.6	2	21	0.289	3	20	34.0
2CRB	97	36	12.6	0.355	40.2	2	31	8.3	2	31	0.482	2	31	47.4
1E68	70	32	10.4	0.272	33.2	3	18	8.5	2	19	0.409	2	19	41.8
1ENH	54	29	19.6	0.276	31.9	5	12	7.7	5	12	0.308	5	12	42.6
1EO0	77	40	10.1	0.258	29.6	4	10	10.0	4	10	0.300	4	10	32.5
1FEX	59	30	10.5	0.303	37.7	5	12	6.7	5	12	0.379	5	12	52.1
1GAB	53	59	10.7	0.384	47.2	3	10	5.3	3	10	0.413	3	10	54.7
2HEP	42	34	11.0	0.380	49.4	3	17	3.8	1	34	0.380	3	17	60.7
1HYP	75	33	12.6	0.297	35.0	2	27	7.6	2	27	0.324	2	27	37.3
1J7O	76	39	8.8	0.305	35.9	3	19	8.6	3	19	0.343	3	19	37.8
1K40	126	39	11.1	0.323	27.8	5	9	5.2	5	9	0.507	5	9	44.4
1KOY	62	35	10.0	0.344	39.1	5	13	5.8	1	35	0.344	5	13	42.3
2L09	62	32	6.8	0.368	46.4	1	32	6.8	1	32	0.368	1	32	46.4
1L2Y	20	26	3.7	0.294	70.0	4	15	1.6	5	8	0.296	4	15	83.8
1LQ7	67	48	4.5	0.386	50.4	1	48	4.5	1	48	0.386	1	48	50.4
1P68	102	29	10.4	0.388	35.5	4	12	3.7	5	11	0.586	4	12	57.8
1POU	71	24	11.5	0.261	30.6	3	21	6.8	3	21	0.328	3	21	39.4
1PPT	36	27	8.4	0.447	57.6	4	19	5.9	1	27	0.447	4	19	59.7
1PRU	56	43	6.8	0.365	46.4	1	43	6.8	1	43	0.365	1	43	46.4
1RES	43	30	5.9	0.260	46.5	1	30	5.9	5	6	0.304	1	30	46.5
1RIJ	23	36	4.3	0.223	63.0	2	25	2.6	3	23	0.235	2	25	68.5
2YGS	92	30	9.6	0.363	36.7	1	30	9.6	1	30	0.363	1	30	36.7
1YRF	35	25	4.9	0.290	54.3	5	10	4.0	5	10	0.417	5	10	64.3

[a]The contribution of the family of the respective model to the conformational ensemble at T=300 K.
[b]The rank of the respective family (according to population).

Table S4: Characteristics of the first (top-probability) and measure-best models of the structures of the benchmark proteins obtained using UNRES with multitorsional potential: parameter set A, $w_{mtor} = 0.20$.

protein	number of	First model				Measure-best model								
	residues	P [%][a]	RMSD [Å]	TM-score	GDT-TS	ncl[b]	P [%][a]	RMSD [Å]	ncl[b]	P [%][a]	TM-score	ncl[b]	P [%][a]	GDT-TS
1A1W	83	32	12.9	0.213	25.3	5	14	10.7	5	14	0.318	5	14	33.4
1A6S	87	38	10.4	0.228	25.6	1	38	10.4	4	13	0.232	1	38	25.6
1ACP	77	50	12.5	0.253	26.0	2	17	10.5	3	12	0.282	3	12	33.1
1BBL	37	33	8.9	0.238	38.5	2	24	6.7	2	24	0.270	2	24	43.9
1BG8	76	57	11.0	0.204	23.7	1	57	11.0	4	6	0.235	2	21	25.3
1CLB	75	27	11.4	0.229	26.7	4	17	8.7	5	10	0.317	4	17	35.3
2CRB	97	35	8.2	0.473	50.0	1	35	8.2	1	35	0.473	1	35	50.0
1E68	70	26	10.8	0.370	40.0	4	18	5.6	4	18	0.383	4	18	47.5
1ENH	54	27	18.3	0.269	33.3	5	5	10.8	4	19	0.296	4	19	40.3
1EO0	77	28	14.1	0.285	30.5	3	19	9.4	3	19	0.336	3	19	38.0
1FEX	59	28	11.2	0.284	37.3	4	11	6.0	4	11	0.350	4	11	48.3
1GAB	53	61	11.0	0.380	47.6	5	4	4.8	5	4	0.457	5	4	56.1
2HEP	42	39	14.6	0.398	47.6	4	12	2.6	4	12	0.497	4	12	70.8
1HYP	75	31	10.3	0.268	31.3	2	27	8.6	2	27	0.303	2	27	39.3
1J7O	76	51	12.4	0.254	28.0	3	12	10.8	3	12	0.282	2	18	31.2
1K40	126	33	15.1	0.247	20.4	3	12	7.1	3	12	0.357	3	12	35.7
1KOY	62	37	9.8	0.326	41.9	2	21	6.5	2	21	0.367	2	21	45.6
2L09	62	37	7.4	0.363	44.0	1	37	7.4	3	19	0.383	3	19	45.2
1L2Y	20	31	2.4	0.249	77.5	1	31	2.4	5	9	0.253	1	31	77.5
1LQ7	67	42	4.7	0.336	49.6	3	18	4.2	3	18	0.386	3	18	51.9
1P68	102	33	8.1	0.377	37.0	3	22	4.0	3	22	0.557	3	22	52.9
1POU	71	32	11.6	0.338	35.2	2	28	5.8	2	28	0.422	2	28	48.2
1PPT	36	28	8.3	0.475	59.7	2	23	5.4	4	13	0.479	4	13	60.4
1PRU	56	25	6.6	0.361	46.4	1	25	6.6	1	25	0.361	1	25	46.4
1RES	43	41	5.9	0.227	43.6	3	14	5.8	5	4	0.276	3	14	44.8
1RIJ	23	29	5.8	0.256	63.0	2	29	4.0	3	22	0.262	2	29	67.4
2YGS	92	26	13.7	0.300	29.6	3	17	10.7	3	17	0.376	3	17	38.9
1YRF	35	28	7.0	0.340	49.3	3	24	4.3	2	25	0.430	3	24	64.3

[a]The contribution of the family of the respective model to the conformational ensemble at T=300 K.

[b]The rank of the respective family (according to population).

Table S5: Characteristics of the first (top-probability) and measure-best models of the structures of the benchmark proteins obtained using UNRES with multitorsional potential: parameter set B, $w_{mtor} = 0.10$.

protein	number of residues	First model				Measure-best model								
		P [%][a]	RMSD [Å]	TM-score	GDT-TS	ncl[b]	P [%][a]	RMSD [Å]	ncl[b]	P [%][a]	TM-score	ncl[b]	P [%][a]	GDT-TS
1A1W	83	42	13.3	0.232	24.7	3	12	11.6	2	29	0.255	4	11	28.0
1A6S	87	55	11.9	0.215	24.1	1	55	11.9	1	55	0.215	1	55	24.1
1ACP	77	42	12.8	0.250	28.9	3	19	10.4	1	42	0.250	3	19	30.2
1BBL	37	24	7.0	0.262	46.0	4	19	6.5	3	20	0.273	1	24	46.0
1BG8	76	63	11.6	0.197	23.4	4	7	10.9	5	4	0.246	3	11	27.0
1CLB	75	32	12.0	0.215	24.3	3	17	8.2	3	17	0.301	3	17	36.3
2CRB	97	28	9.6	0.427	46.1	2	24	8.5	1	28	0.427	1	28	46.1
1E68	70	29	8.9	0.280	34.3	5	12	6.6	3	18	0.370	3	18	41.1
1ENH	54	32	12.5	0.281	40.3	2	25	6.5	2	25	0.332	2	25	44.9
1EO0	77	23	14.2	0.252	28.6	4	21	9.3	2	23	0.376	2	23	40.3
1FEX	59	36	10.4	0.299	41.1	3	17	5.9	3	17	0.363	3	17	48.7
1GAB	53	46	11.5	0.359	45.8	3	14	4.4	3	14	0.398	3	14	57.1
2HEP	42	35	3.0	0.426	67.3	1	35	3.0	1	35	0.426	1	35	67.3
1HYP	75	62	10.1	0.243	31.3	3	8	9.5	3	8	0.362	3	8	41.0
1J7O	76	33	12.4	0.231	28.6	2	27	9.8	5	6	0.268	2	27	31.2
1K40	126	61	17.9	0.205	17.7	4	8	6.6	4	8	0.407	4	8	38.9
1KOY	62	26	6.4	0.287	39.1	1	26	6.4	2	25	0.333	2	25	39.9
2L09	62	33	13.1	0.209	29.0	3	21	8.6	3	21	0.335	3	21	41.5
1L2Y	20	32	5.5	0.323	67.5	2	24	2.1	1	32	0.323	2	24	78.8
1LQ7	67	29	9.0	0.319	40.3	2	28	4.3	2	28	0.368	2	28	50.4
1P68	102	39	10.4	0.353	31.4	3	16	3.7	3	16	0.599	3	16	58.3
1POU	71	30	7.3	0.352	41.5	1	30	7.3	1	30	0.352	1	30	41.5
1PPT	36	32	10.2	0.273	39.6	3	21	5.7	3	21	0.443	3	21	62.5
1PRU	56	36	6.7	0.346	44.2	1	36	6.7	1	36	0.346	1	36	44.2
1RES	43	34	5.9	0.226	42.4	1	34	5.9	4	15	0.285	3	16	42.4
1RIJ	23	33	4.9	0.223	66.3	2	25	2.7	2	25	0.228	2	25	70.7
2YGS	92	39	12.7	0.248	26.9	3	15	10.4	2	24	0.282	4	14	28.5
1YRF	35	39	7.4	0.334	52.1	5	5	4.2	5	5	0.404	5	5	61.4

[a]The contribution of the family of the respective model to the conformational ensemble at T=300 K.
[b]The rank of the respective family (according to population).

Table S6: Characteristics of the first (top-probability) and measure-best models of the structures of the benchmark proteins obtained using UNRES with multitorsional potential: parameter set B, $w_{mtor} = 0.15$.

protein	number of residues	First model				Measure-best model								
		P [%][a]	RMSD [Å]	TM-score	GDT-TS	ncl[b]	P [%][a]	RMSD [Å]	ncl[b]	P [%][a]	TM-score	ncl[b]	P [%][a]	GDT-TS
1A1W	83	28	9.3	0.275	29.5	1	28	9.3	3	21	0.305	3	21	31.3
1A6S	87	68	11.8	0.197	19.8	2	10	10.3	2	10	0.270	3	7	27.3
1ACP	77	58	13.1	0.270	28.9	2	14	10.0	1	58	0.270	2	14	32.8
1BBL	37	25	6.3	0.231	41.2	2	23	6.2	2	23	0.271	2	23	46.0
1BG8	76	50	11.3	0.216	24.7	5	2	10.9	2	25	0.223	2	25	27.3
1CLB	75	32	9.1	0.262	29.7	3	20	8.7	3	20	0.320	3	20	36.7
2CRB	97	46	8.9	0.443	48.2	3	18	8.7	3	18	0.464	3	18	50.8
1E68	70	24	9.6	0.394	41.8	2	24	7.3	1	24	0.394	1	24	41.8
1ENH	54	31	13.7	0.279	38.0	4	13	5.1	4	13	0.466	4	13	58.8
1EO0	77	30	9.3	0.337	37.7	4	13	8.3	1	30	0.337	1	30	37.7
1FEX	59	31	10.8	0.263	35.2	3	16	7.9	3	16	0.290	3	16	41.1
1GAB	53	49	10.6	0.304	42.5	3	16	5.8	3	16	0.410	3	16	50.9
2HEP	42	28	3.0	0.407	61.9	1	28	3.0	2	24	0.433	1	28	61.9
1HYP	75	34	7.4	0.317	37.0	1	34	7.4	1	34	0.317	1	34	37.0
1J7O	76	32	10.5	0.259	29.6	2	28	9.7	2	28	0.291	2	28	33.2
1K40	126	30	15.7	0.235	19.8	3	20	14.3	3	20	0.284	3	20	27.0
1KOY	62	24	11.5	0.270	31.4	2	22	5.8	2	22	0.378	2	22	48.0
2L09	62	30	13.1	0.351	40.7	2	29	7.9	1	30	0.351	1	30	40.7
1L2Y	20	27	6.0	0.242	65.0	3	22	1.8	2	23	0.291	3	22	80.0
1LQ7	67	44	4.0	0.400	54.9	1	44	4.0	2	20	0.413	1	44	54.9
1P68	102	28	10.4	0.413	37.0	3	21	4.3	4	14	0.530	3	21	52.0
1POU	71	26	5.2	0.401	47.5	1	26	5.2	1	26	0.401	1	26	47.5
1PPT	36	25	8.5	0.420	56.2	4	20	6.2	2	25	0.446	4	20	59.0
1PRU	56	42	6.9	0.358	45.1	2	22	6.5	1	42	0.358	1	42	45.1
1RES	43	28	6.4	0.241	43.0	2	26	5.9	3	19	0.269	1	28	43.0
1RIJ	23	37	3.7	0.289	66.3	2	25	3.7	1	37	0.289	2	25	68.5
2YGS	92	47	8.3	0.353	37.5	1	47	8.3	1	47	0.353	1	47	37.5
1YRF	35	36	5.1	0.322	55.7	4	9	3.9	4	9	0.402	4	9	63.6

[a]The contribution of the family of the respective model to the conformational ensemble at T=300 K.
[b]The rank of the respective family (according to population).

Table S7: Characteristics of the first (top-probability) and measure-best models of the structures of the benchmark proteins obtained using UNRES with multitorsional potential: parameter set B, $w_{mtor} = 0.20$.

protein	number of residues	First model				Measure-best model								
		P [%][a]	RMSD [Å]	TM-score	GDT-TS	ncl[b]	P [%][a]	RMSD [Å]	ncl[b]	P [%][a]	TM-score	ncl[b]	P [%][a]	GDT-TS
1A1W	83	28	13.5	0.242	25.9	4	14	9.3	4	14	0.401	4	14	39.2
1A6S	87	63	12.3	0.205	21.6	3	9	11.9	3	9	0.225	5	5	23.6
1ACP	77	41	12.8	0.262	29.9	3	15	10.2	1	41	0.262	3	15	33.4
1BBL	37	26	8.5	0.214	39.2	5	16	6.5	2	20	0.243	2	20	43.9
1BG8	76	46	12.8	0.182	21.1	5	3	11.0	5	3	0.247	5	3	27.0
1CLB	75	45	12.1	0.223	26.7	2	20	8.3	4	11	0.311	4	11	34.7
2CRB	97	30	10.1	0.377	40.2	3	17	8.9	3	17	0.406	5	10	45.4
1E68	70	41	9.8	0.338	37.5	1	41	9.8	1	41	0.338	1	41	37.5
1ENH	54	28	15.6	0.283	37.5	5	13	6.9	5	13	0.315	5	13	44.9
1EO0	77	25	14.2	0.261	29.2	2	25	10.0	3	24	0.278	3	24	33.4
1FEX	59	38	11.0	0.263	37.3	4	10	6.9	4	10	0.284	4	10	42.0
1GAB	53	51	11.0	0.341	43.9	3	15	5.9	3	15	0.368	3	15	48.6
2HEP	42	33	12.6	0.375	48.2	2	28	2.6	2	28	0.545	2	28	72.6
1HYP	75	31	12.0	0.304	32.3	2	25	7.8	2	25	0.316	2	25	40.3
1J7O	76	30	12.6	0.263	29.9	5	6	9.6	3	20	0.316	3	20	33.2
1K40	126	36	14.6	0.260	22.8	2	23	10.8	2	23	0.318	2	23	27.4
1KOY	62	35	9.3	0.351	41.5	4	12	5.9	1	35	0.351	4	12	41.5
2L09	62	34	10.4	0.273	37.5	2	30	7.2	5	5	0.361	2	30	43.1
1L2Y	20	24	2.8	0.260	73.8	4	19	1.9	4	19	0.313	4	19	82.5
1LQ7	67	40	3.9	0.421	54.1	1	40	3.9	1	40	0.421	1	40	54.1
1P68	102	24	3.7	0.594	55.6	1	24	3.7	1	24	0.594	1	24	55.6
1POU	71	31	7.8	0.326	39.1	1	31	7.8	5	9	0.345	1	31	39.1
1PPT	36	23	8.7	0.421	55.6	2	22	6.3	5	16	0.421	2	22	58.3
1PRU	56	33	6.8	0.336	44.6	4	16	6.3	1	33	0.336	4	16	44.6
1RES	43	36	6.6	0.247	41.9	2	25	5.7	3	18	0.271	2	25	43.0
1RIJ	23	27	2.2	0.237	75.0	1	27	2.2	3	19	0.255	1	27	75.0
2YGS	92	43	13.0	0.270	28.5	3	14	10.7	3	14	0.369	3	14	36.4
1YRF	35	23	7.0	0.314	54.3	2	23	4.0	5	15	0.365	2	23	63.6

[a]The contribution of the family of the respective model to the conformational ensemble at T=300 K.
[b]The rank of the respective family (according to population).

Table S8: Characteristics of the first (top-probability) and measure-best models of the structures of the benchmark proteins obtained using UNRES with multitorsional potential: parameter set C, $w_{mtor} = 0.10$.

protein	number of residues	First model				Measure-best model								
		P [%][a]	RMSD [Å]	TM-score	GDT-TS	ncl[b]	P [%][a]	RMSD [Å]	ncl[b]	P [%][a]	TM-score	ncl[b]	P [%][a]	GDT-TS
1A1W	83	30	13.3	0.222	24.7	4	11	10.2	4	11	0.267	4	11	29.5
1A6S	87	53	11.5	0.189	19.0	1	53	11.5	3	11	0.226	2	20	23.6
1ACP	77	35	12.9	0.248	29.6	3	16	9.6	3	16	0.297	3	16	33.1
1BBL	37	33	6.6	0.212	41.2	4	16	6.4	5	11	0.245	4	16	42.6
1BG8	76	40	11.5	0.223	24.7	4	8	11.1	4	8	0.229	3	23	26.6
1CLB	75	37	11.9	0.219	26.0	5	8	7.1	5	8	0.337	5	8	38.3
2CRB	97	33	9.4	0.396	44.6	3	20	8.3	3	20	0.473	3	20	47.4
1E68	70	39	10.1	0.334	37.1	5	5	9.9	1	39	0.334	1	39	37.1
1ENH	54	32	13.6	0.266	38.4	3	19	4.7	3	19	0.463	3	19	60.2
1EO0	77	23	14.1	0.297	33.1	5	15	8.6	4	18	0.315	5	15	34.4
1FEX	59	36	10.6	0.285	39.0	3	23	7.0	3	23	0.289	3	23	43.2
1GAB	53	40	10.9	0.318	42.9	3	16	4.1	3	16	0.454	3	16	59.4
2HEP	42	33	4.3	0.381	62.5	1	33	4.3	1	33	0.381	1	33	62.5
1HYP	75	47	9.8	0.258	30.7	1	47	9.8	4	9	0.309	4	9	35.0
1J7O	76	36	10.2	0.249	29.9	2	30	9.1	2	30	0.301	2	30	33.9
1K40	126	40	15.1	0.247	19.8	3	23	8.0	3	23	0.369	3	23	34.3
1KOY	62	27	5.8	0.326	41.9	1	27	5.8	1	27	0.326	3	20	42.7
2L09	62	27	9.1	0.252	32.7	1	27	9.1	3	16	0.283	3	16	34.7
1L2Y	20	25	4.4	0.322	68.8	5	13	2.0	1	25	0.322	5	13	80.0
1LQ7	67	38	4.5	0.383	50.8	1	38	4.5	1	38	0.383	1	38	50.8
1P68	102	32	8.3	0.360	37.0	5	12	4.5	3	17	0.567	3	17	54.7
1POU	71	31	6.7	0.330	39.8	4	12	6.2	4	12	0.374	4	12	44.0
1PPT	36	39	8.4	0.399	55.6	2	19	6.5	1	39	0.399	2	19	56.9
1PRU	56	30	7.2	0.343	43.8	3	19	6.7	1	30	0.343	3	19	44.6
1RES	43	35	6.0	0.242	43.6	1	35	6.0	3	18	0.273	1	35	43.6
1RIJ	23	29	4.4	0.250	65.2	2	27	3.2	2	27	0.266	2	27	66.3
2YGS	92	34	11.5	0.261	27.7	5	13	11.3	2	19	0.294	2	19	28.8
1YRF	35	27	5.7	0.361	54.3	2	23	5.3	2	23	0.428	2	23	60.7

[a]The contribution of the family of the respective model to the conformational ensemble at T=300 K.
[b]The rank of the respective family (according to population).

Table S9: Characteristics of the first (top-probability) and measure-best models of the structures of the benchmark proteins obtained using UNRES with multitorsional potential: parameter set C, $w_{mtor} = 0.15$.

protein	number of residues	First model				Measure-best model								
		P [%][a]	RMSD [Å]	TM-score	GDT-TS	ncl[b]	P [%][a]	RMSD [Å]	ncl[b]	P [%][a]	TM-score	ncl[b]	P [%][a]	GDT-TS
1A1W	83	26	11.7	0.253	27.7	3	22	11.3	5	12	0.320	5	12	32.8
1A6S	87	35	12.3	0.210	22.1	4	13	10.6	3	14	0.223	3	14	23.3
1ACP	77	41	12.2	0.252	27.6	2	21	10.5	4	12	0.277	2	21	32.1
1BBL	37	31	9.1	0.216	38.5	3	14	6.3	2	26	0.245	4	14	43.9
1BG8	76	50	11.0	0.225	23.7	1	50	11.0	2	22	0.234	3	17	25.7
1CLB	75	35	10.0	0.279	32.7	4	16	5.5	4	16	0.390	4	16	45.3
2CRB	97	34	8.2	0.470	51.0	1	34	8.2	1	34	0.470	1	34	51.0
1E68	70	29	10.1	0.315	35.7	5	11	7.5	2	24	0.390	2	24	42.1
1ENH	54	38	14.8	0.281	36.1	2	19	6.2	2	19	0.358	2	19	49.1
1EO0	77	26	14.8	0.241	29.9	3	22	8.3	2	26	0.327	3	22	36.4
1FEX	59	38	10.3	0.276	36.9	2	20	8.0	5	11	0.300	2	20	38.6
1GAB	53	45	10.9	0.364	45.8	2	19	4.2	2	19	0.397	2	19	57.1
2HEP	42	26	2.6	0.503	70.2	1	26	2.6	1	26	0.503	1	26	70.2
1HYP	75	38	11.3	0.259	32.3	2	26	6.7	3	23	0.341	3	23	42.7
1J7O	76	26	11.3	0.268	30.9	2	22	9.5	5	12	0.322	5	12	38.5
1K40	126	54	13.7	0.262	21.6	3	13	13.6	3	13	0.333	3	13	27.6
1KOY	62	29	10.4	0.278	35.1	3	20	6.9	2	22	0.354	2	22	43.1
2L09	62	27	8.4	0.390	46.0	1	27	8.4	1	27	0.390	1	27	46.0
1L2Y	20	28	6.3	0.303	63.8	2	26	2.4	1	28	0.303	2	26	73.8
1LQ7	67	47	4.9	0.332	48.1	1	47	4.9	4	11	0.359	1	47	48.1
1P68	102	29	8.0	0.380	39.2	3	22	4.5	3	22	0.539	3	22	52.9
1POU	71	23	10.1	0.234	29.9	2	23	5.2	2	23	0.385	2	23	45.8
1PPT	36	22	8.6	0.433	57.6	3	22	5.7	3	22	0.462	3	22	61.8
1PRU	56	30	10.6	0.333	42.0	3	23	6.4	4	14	0.352	3	23	44.2
1RES	43	35	6.6	0.257	42.4	2	21	6.2	4	12	0.272	5	10	44.8
1RIJ	23	34	4.8	0.221	60.9	2	29	3.7	3	24	0.298	3	24	64.1
2YGS	92	26	13.2	0.232	25.8	5	13	11.8	2	25	0.298	2	25	31.8
1YRF	35	33	7.1	0.317	52.1	3	20	4.2	4	13	0.375	3	20	62.9

[a]The contribution of the family of the respective model to the conformational ensemble at T=300 K.
[b]The rank of the respective family (according to population).

Table S10: Characteristics of the first (top-probability) and measure-best models of the structures of the benchmark proteins obtained using UNRES with multitorsional potential: parameter set C, $w_{mtor} = 0.20$.

protein	number of residues	First model				Measure-best model								
		P [%][a]	RMSD [Å]	TM-score	GDT-TS	ncl[b]	P [%][a]	RMSD [Å]	ncl[b]	P [%][a]	TM-score	ncl[b]	P [%][a]	GDT-TS
1A1W	83	44	13.4	0.215	24.7	2	21	11.9	5	8	0.256	3	16	27.1
1A6S	87	50	13.4	0.192	20.4	4	11	10.1	4	11	0.225	4	11	23.3
1ACP	77	53	12.8	0.266	28.2	2	15	10.6	3	14	0.270	2	15	30.8
1BBL	37	31	9.6	0.220	35.8	3	17	6.5	3	17	0.237	3	17	41.9
1BG8	76	68	13.0	0.220	24.3	5	2	12.1	5	2	0.228	2	10	26.6
1CLB	75	34	9.7	0.267	31.7	4	13	7.5	4	13	0.303	2	25	35.7
2CRB	97	39	9.5	0.434	47.2	3	24	8.9	1	39	0.434	1	39	47.2
1E68	70	38	9.7	0.386	41.8	4	8	7.8	1	38	0.386	1	38	41.8
1ENH	54	33	15.9	0.286	36.1	4	12	10.7	2	24	0.315	2	24	40.7
1EO0	77	23	14.7	0.252	29.2	3	20	9.6	4	18	0.302	3	20	31.8
1FEX	59	31	10.3	0.291	38.6	3	21	6.3	3	21	0.319	3	21	47.0
1GAB	53	36	11.7	0.359	47.2	3	21	5.2	3	21	0.401	3	21	52.8
2HEP	42	24	3.6	0.404	63.1	1	24	3.6	4	19	0.421	2	22	63.7
1HYP	75	25	11.5	0.263	31.7	2	21	6.5	3	19	0.332	3	19	44.0
1J7O	76	40	10.5	0.333	36.8	4	13	9.1	1	40	0.333	1	40	36.8
1K40	126	37	14.3	0.209	18.1	5	2	5.1	5	2	0.481	5	2	42.9
1KOY	62	33	6.2	0.356	44.8	1	33	6.2	2	18	0.359	1	33	44.8
2L09	62	36	10.2	0.252	35.5	3	16	9.9	4	11	0.300	1	36	35.5
1L2Y	20	28	2.4	0.337	77.5	1	28	2.4	2	24	0.384	1	28	77.5
1LQ7	67	38	4.1	0.386	51.9	1	38	4.1	1	38	0.386	1	38	51.9
1P68	102	30	4.0	0.549	53.7	1	30	4.0	1	30	0.549	1	30	53.7
1POU	71	38	11.6	0.261	30.3	5	9	7.5	4	13	0.309	4	13	38.4
1PPT	36	24	9.1	0.439	56.9	4	20	5.6	1	24	0.439	4	20	59.7
1PRU	56	40	6.7	0.337	42.4	1	40	6.7	1	40	0.337	1	40	42.4
1RES	43	27	6.4	0.243	41.9	2	26	5.9	3	25	0.275	2	26	42.4
1RIJ	23	29	5.2	0.201	56.5	3	21	2.4	3	21	0.243	3	21	71.7
2YGS	92	38	10.4	0.268	28.5	1	38	10.4	4	15	0.301	2	22	30.2
1YRF	35	35	7.0	0.376	57.9	3	18	5.5	3	18	0.413	3	18	58.6

[a]The contribution of the family of the respective model to the conformational ensemble at T=300 K.

[b]The rank of the respective family (according to population).
